# Transcriptome Analyses of Prophage in Mediating Persistent Methicillin-Resistant *Staphylococcus aureus* Endovascular Infection

**DOI:** 10.3390/genes13091527

**Published:** 2022-08-25

**Authors:** Yi Li, Liang Chen, Fengli Zhu, Arnold S. Bayer, Yan Q. Xiong

**Affiliations:** 1 The Lundquist Institute for Biomedical Innovation at Harbor-UCLA Medical Center, Torrance, CA 90502, USA; 2 Hackensack Meridian Health Center for Discovery and Innovation, Nutley, NJ 07110, USA; 3 Hackensack Meridian School of Medicine, Nutley, NJ 07110, USA; 4 David Geffen School of Medicine at UCLA, Los Angeles, CA 90095, USA

**Keywords:** MRSA-persistent infection, prophage, RNA sequencing

## Abstract

Persistent methicillin-resistant *Staphylococcus aureus* (MRSA) endovascular infections represent a significant subset of *S. aureus* infections and correlate with exceptionally high mortality. We have recently demonstrated that the lysogenization of prophage ϕSA169 from a clinical persistent MRSA bacteremia isolate (300-169) into a clinical resolving bacteremia MRSA isolate (301-188) resulted in the acquisition of well-defined in vitro and in vivo phenotypic and genotypic profiles related to persistent outcome. However, the underlying mechanism(s) of this impact is unknown. In the current study, we explored the genetic mechanism that may contribute to the ϕSA169-correlated persistence using RNA sequencing. Transcriptomic analyses revealed that the most significant impacts of ϕSA169 were: (i) the enhancement of fatty acid biosynthesis and purine and pyrimidine metabolic pathways; (ii) the repression of galactose metabolism and phosphotransferase system (PTS); and (iii) the down-regulation of the mutual prophage genes in both 300-169 and 301-188 strains. In addition, the influence of different genetic backgrounds between 300-169 and 301-188 might also be involved in the persistent outcome. These findings may provide targets for future studies on the persistence of MRSA.

## 1. Introduction

Methicillin-resistant *S. aureus* (MRSA) is a major cause of life-threatening endovascular infections, including bacteremia and infective endocarditis (IE) [1,2]. Persistent MRSA bacteremia (PB; defined as ≥5 days of positive blood cultures in the presence of antibiotic therapy) represents ~15 to 30% of such infections [3,4]. In addition, it is very worrisome that most PB isolates appear to be susceptible in vitro to gold-standard anti-MRSA antibiotics (e.g., vancomycin (VAN) and daptomycin (DAP)) by the Clinical and Laboratory Standards Institute (CLSI) breakpoints [4,5,6], yet persistent in vivo. Thus, PB represents a uniquely vital variant of traditional antibiotic resistance mechanisms. This problem underscores an urgent need to understand the mechanism(s) of specific factors driving this syndrome.

Prophages can modify their bacterial host’s lifestyle, fitness, virulence, and pathogenesis in numerous ways [7,8,9,10]. We recently discovered a novel prophage ϕSA169 that exists in a clinical PB isolate (300-169), while is not present in a genetically matched (clonal complex 45 (CC45), *agr* I, and SCC*mec* IV) clinical resolving MRSA bacteremia strain (RB, defined as initial MRSA bacteremia resolved within 2–4 days of antibiotic treatment; 301-188) [4,11,12]. In addition, whole-genome sequencing (WGS) analyses demonstrated that besides the ϕSA169, both PB 300-169 and RB 301-188 strains carry an identical mutual prophage [12]. Importantly, the lysogenization of RB 300-188 by ϕSA169 (301-188::ϕSA169) leads to this latter construct having “PB-like” phenotypes and genotypes similar to PB 300-169 strain both in vitro (e.g., higher growth rate, lower ATP levels, stronger biofilm formation and δ-hemolysin activity, earlier activation of global regulators, and higher expression of purine biosynthesis gene *purF*) and in an experimental IE model [11]. However, the fundamental mechanisms of the ϕSA169-driven PB outcomes remain unknown.

The current study aimed to define the impact of ϕSA169 on genetic factors which may contribute to the PB phenotypes by RNA sequencing (RNA-seq) using PB 300-169 wild type (WT), RB 301-188 WT, and ϕSA169 lysogenized RB 301-188 (301-188::ϕSA169) strains. The transcriptomic analyses emphasized genetic factors that might contribute to the PB outcomes and provided clues for future studies on molecular mechanisms of PB outcomes.

## 2. Materials and Methods

### 2.1. Bacterial Strains, Plasmids, and Growth Medium

Three MRSA strains, including PB 300-169 WT (300-169), RB 301-188 WT (301-188), and 301-188 WT ϕSA169 lysogenization (301-188::ϕSA169), were used in our previous [11] and current studies. The PB 300-169 strain was isolated from a patient with 16 days of persistent MRSA bacteremia, while the RB 301-188 strain was obtained from a patient with 2 days of MRSA bacteremia [4]. In addition, all the three study strains have a minimum inhibitory concentration (MIC) to VAN of 0.5 µg/mL and are susceptible to VAN in vitro based upon the CLSI breakpoints [11]. The strains were routinely grown at 37 °C in tryptic soy broth (TSB; Becton Dickinson and Company, NJ, USA) or on tryptic soy agar (TSA) plates if not otherwise specified.

### 2.2. RNA Isolation

RNA isolation was performed following the method described in previous studies [13,14]. In brief, overnight cultured cells of the study strains were pelleted by centrifugation and resuspended in Buffer RLT from RNeasy kit (Qiagen, Germantown, MD, USA), and then transferred into lysing matrix B (MP Biomedicals, Irvine, CA, USA) containing 0.1 mm silica spheres for mechanical lysis using Fastprep (Thermo Fisher, Waltham, MA, USA). Total RNA was isolated according to the manufacturer’s instructions of the RNeasy kit. DNA in the samples was removed using a TURBO^TM^ DNase kit (Thermo Fisher, Waltham, MA, USA) [11]. Biological duplicates from two different experiments were prepared for each study strain. RNA samples with concentrations ≥ 100 ng/μL and 260/280 ratio between 1.9 and 2.0 were submitted to the Novogene Corporation Inc (Sacramento, CA, USA) for RNA-seq.

### 2.3. RNA-Seq and Data Analyses

RNA degradation, purity, integrity, and quantitation were checked prior to the RNA-seq. RNA-seq libraries were constructed using NEBNext^®^Ultra^TM^ RNA Library Prep Kit for Illumina^®^ (NEB, Ipswich, WA, USA). The index-coded samples were clustered using the PE Cluster Kit cBot-HS (Illumina, San Diego, CA, USA) on a cBot Cluster Generation System. Then, the samples were sequenced, and paired-end reads were obtained. For data analyses, RNA-seq reads were mapped to the genome of the PB 300-169 strain (Accession: JASL00000000) [12] using Bowtie2 [15]. Analyses of differential expressions between any two study strains (two biological replicates per study strain) were performed using DESeq2 R package based on a negative binomial distribution. The resulting *p* values were adjusted using Benjamini and Hochberg’s approach for controlling the false discovery rate. The genes with an adjusted *p* value (*p* adj) ≤ 0.05 and |log_2_(fold change)| > 0 were defined as differentially expressed genes (DEGs), indicating the genes had significantly different expression levels in the two strains comparison. The DEGs list generated from the comparison of transcriptomic profiles between the isogenic strain set (301-188 and 301-188::ϕSA169) indicated the impact of ϕSA169. In addition, comparisons of 300-169 vs. 301-188 and 300-169 vs. 301-188::ϕSA169 were also performed to further investigate the role of the distinct genetic backgrounds on the transcriptional changes. The DEGs were classified using the Kyoto Encyclopedia of Genes and Genomes (KEGG) mapper tool with the ST45 mode strain of MRSA CA-347 [16].

### 2.4. Verification of RNA-Seq Results by qRT-PCR

The expression levels of selected genes from the DEGs listed above were confirmed by qRT-PCR as described previously [11,17,18]. The expression of *gyrB* was used as a well-studied host gene to normalize transcripts levels, and relative expression was calculated by the ΔΔC_T_ method [5]. The relative expression level was then used to calculate the fold changes in the selected genes in strain comparisons.

## 3. Results

### 3.1. Global Analyses of Gene Expression

**Figure 1:**
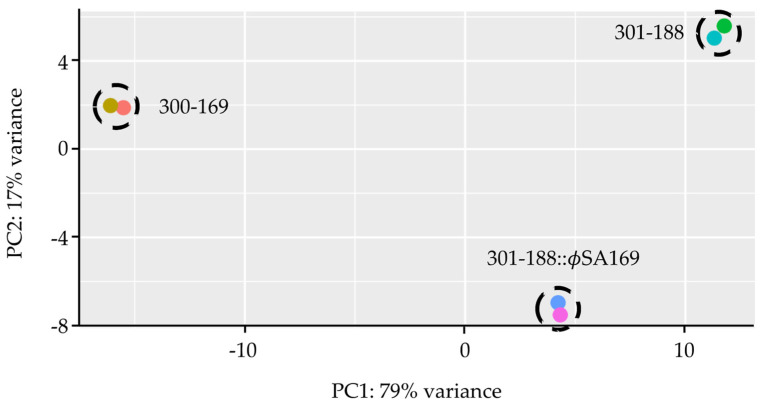
Principle component analysis (PCA) of RNA-seq results of 300-169, 301-188, and 301-188::ϕSA169 strains. The X-axis represents the first principal component (PC1) that displays the maximum variation through the data, while the Y-axis represents the second principal component (PC2) that displays the next highest variation. Each dot represents a biological duplicate of a study strain. The replicates of each study strain were well clustered, indicating the good reproducibility of the samples in each strain. RNA-seq results of the study strains were scattered in the graph, which indicated the significant genetic variations of the study strains.

Each sample yielded a high percentage of exon-mapped reads (85.3–90.1%) that covered over 2000 genes, indicating the abundance of mRNA and low interference from non-coding RNAs. More than 86% of the mapped genes had at least one fragment per kilobase of transcript sequence per million (FPKM), suggesting that the transcriptional profiles covered most of the genes in the study strains. Principal component analysis (PCA) was performed to assess the overall differences in the gene expression of the study strains (Figure 1). Among the study strains, 300-169 had a different genetic background vs. 301-188, while 301-188 and 301-188::ϕSA169 were isogenic strain-set with the only difference in the absence/presence of ϕSA169. The strains 301-169 and 301-188 had the most distant locations on the PCA biplot, indicating the most significant genetic variation, while 301-188 and 301-188::ϕSA169 had the closest locations suggesting minor variation, which might be due to the same genetic background (Figure 1).

**Figure 2:**
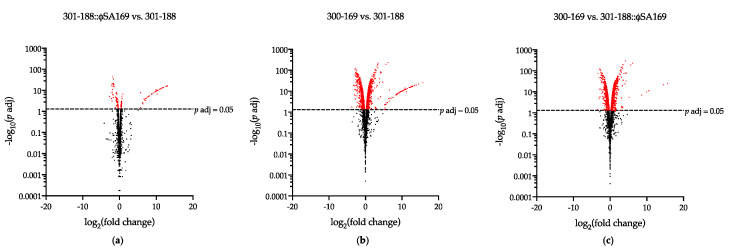
Volcano plots displayed the genes differentially expressed in (**a**) 301-188::ϕSA169 vs. 301-188; (**b**) 300-169 vs. 301-188; (**c**) 300-169 vs. 301-188::ϕSA169. The genes with *p* adj ≤ 0.05 and |log_2_(fold change)| > 0 were defined as differentially expressed genes (DEGs) and were labeled in red.

The transcriptome profiles of each study strain were compared to identify the DEGs (Figure 2, Table 1). There were 153 DEGs in 301-188::ϕSA169 vs. 301-188 (Figure 2a), while over 1200 DEGs were found in 300-169 vs. 301-188 (Figure 2b) and 300-169 vs. 301-188::ϕSA169 (Figure 2c). In the strain 301-188::ϕSA169, 77 and 76 DEGs were significantly up- and down-regulated, respectively, compared to the parental 301-188 (Table 1). Over half of the up-regulated DEGs (49 out of 77) were the genes of ϕSA169 (Table S1), while more than one-third of the down-regulated DEGs (24 out of 76) belonged to the mutual prophage in both 300-169 and 301-188 (Table S2). The high log_2_(fold change) values of the 49 ϕSA169 genes (Table S1) indicated the absence in 301-188. In the 300-169 strain, 666 and 633 DEGs were significantly up- and down-regulated, respectively, compared to 301-188 (Table 1). The detailed up- and down-regulated DEGs in the comparison of 300-169 vs. 301-188 are presented in Tables S3 and S4, respectively. In the comparison of 300-169 vs. 301-188::ϕSA169, a total of 637 and 613 DEGs were significantly up- and down-regulated, respectively (Table 1). The detailed up- and down-regulated DEGs are presented in Tables S5 and S6, respectively.

### 3.2. ϕSA169 Had Similar Transcriptional Profiles in 300-169 and 301-188::ϕSA169 Strains

**Figure 3:**
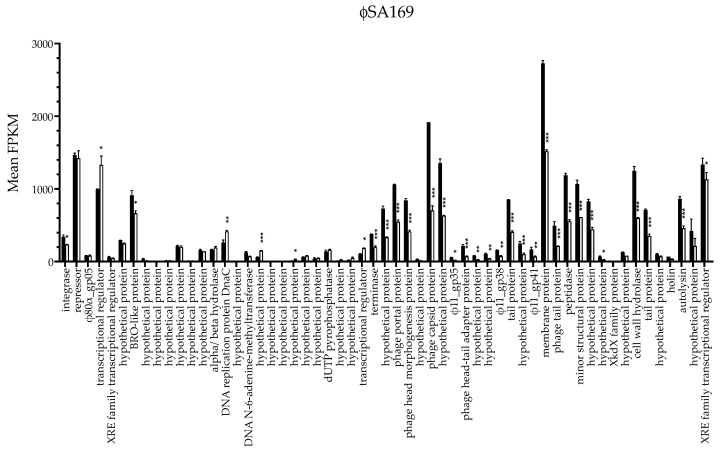
Transcriptional analysis of ϕSA169 genes in 300-169 (black bars) and 301-188::ϕSA169 (white bars). The transcriptional profiles of ϕSA169 in 300-169 and 301-188::ϕSA169 were similar, and genes from the packing and morphogenesis module had higher expression levels than genes from other modules. Expression levels of some ϕSA169 genes, especially the genes from packing and morphogenesis, were significantly higher in 300-169 compared to 301-188::ϕSA169. * *p* adj < 0.05, ** *p* adj < 0.01, *** *p* adj < 0.001.

Prophage ϕSA169 was initially identified in PB 300-169 and transduced into RB 301-188 to construct the 301-188::ϕSA169 strain. Therefore, ϕSA169 was an exogenous genomic element for the 301-188 chromosome despite the similar genetic background between 300-169 and 301-188 (e.g., CC45, *agr* I, and SCC*mec* IV); thus, the gene expression of ϕSA169 may differ in the 300-169 vs. 301-188::ϕSA169. There were 58 out of a total of 67 annotated genes in ϕSA169 detected in the current RNA-seq results (Figure 3). The plotted expression levels of ϕSA169 genes in both 300-169 and 301-188::ϕSA169 are presented in Figure 3. Bacteriophage (phage) genes are highly mosaic and grouped into different modules based on the functions of the gene products (18). In general, ϕSA169 genes in the modules of lysogeny, packing and morphogenesis, and lysis were highly expressed, while genes in the replication module had low expression (Figure 3). In addition, the transcriptional profiles of ϕSA169 were similar in both strains. However, some ϕSA169 genes, especially in the packing and morphogenesis module, had different expression levels in the two strains, which might imply the impact of the distinct genetic backgrounds.

### 3.3. The Impact of ϕSA169 on Transcriptional Profiles

**Figure 4:**
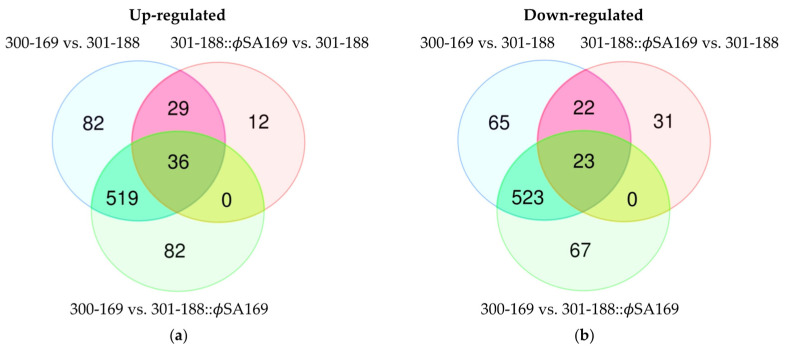
Venn diagram of the DEGs from the comparisons carried out between the study strains. The overlapping DEGs of 300-169 vs. 301-188 and 301-188::ϕSA169 vs. 301-188 might represent the genes specifically affected by ϕSA169, while the overlapping DEGs of 300-169 vs. 301-188 and 300-169 vs. 301-188::ϕSA169 might represent the genes specifically affected by the distinct genetic backgrounds. (**a**) Up-regulated DEGs: 300-169 vs. 301-188 and 301-188::ϕSA169 vs. 301-188 had 65 (29 + 36) overlapping DEGs; 300-169 vs. 301-188 and 300-169 vs. 301-188::ϕSA169 had 555 (519 + 36) overlapping DEGs; all the three comparisons had 36 overlapping DEGs. (**b**) Down-regulated DEGs: 300-169 vs. 301-188 and 301-188::ϕSA169 vs. 301-188 had 45 (22 + 23) overlapping DEGs; 300-169 vs. 301-188 and 300-169 vs. 301-188::ϕSA169 had 546 (523 + 23) overlapping DEGs, all the three comparisons have 23 overlapping DEGs.

The 301-188::ϕSA169 and 301-188 formed an isogenic strain set; thus, the DEGs from the comparison of the two strains were likely caused by ϕSA169. On the other hand, 300-169 and 301-188 strains had distinct genetic backgrounds; thus, the DEGs profile of these two strains might be affected by both ϕSA169 and their genetic backgrounds. Therefore, the overlapping DEGs between the two comparisons (301-188::ϕSA169 vs. 301-188 and 300-169 vs. 301-188) might indicate the specific impact of ϕSA169. There were total of 65 (29 + 36) DEGs up-regulated (Figure 4a) and 45 (22 + 23) DEGs down-regulated (Figure 4b) by the ϕSA169. Most up-regulated DEGs (49 out of 65) belonged to ϕSA169, and the other 16 genes fitted in the MRSA host genes (genes in the chromosome of the study MRSA strains excluding prophage genes) included *purA* and *fatFH* (Table 2). Over half of the down-regulated DEGs (24 out of 45) belonged to the mutual prophage in both 300-169 and 301-188 strains, and the remaining 21 DEGs were the MRSA host genes, including *lacABCDEF*, *treP*, and *pfkB* (Table 3).

### 3.4. The Impact of MRSA Genetic Background on Transcriptional Profiles

The overlapping DEGs of 300-169 vs. 301-188 and 300-169 vs. 301-188::ϕSA169 were analyzed to explore the impact of distinct genetic backgrounds of 300-169 and 301-188 excluding the impact of ϕSA169 (Figure 4). There were 555 (519 + 36) DEGs up-regulated (Figure 4a, Table S7) and 546 (523 + 23) DEGs down-regulated (Figure 4b, Table S8) in these two comparisons. The up-regulated DEGs included 26 genes of ϕSA169 and 6 genes of the mutual prophage in 300-169 and 301-188 (Table S7). The down-regulated DEGs included 3 genes of ϕSA169 and 18 genes of the mutual prophage (Table S8).

### 3.5. DEGs Impacted by Both ϕSA169 and MRSA Genetic Backgrounds

There were 36 (Figure 4a, Table S9) and 23 (Figure 4b, Table S10) DEGs up- and down-regulated in all three comparisons, respectively. It indicated that these DEGs were affected by both ϕSA169 and the genetic backgrounds of 300-169 and 301-188. The up-regulated DEGs included 26 genes in ϕSA169 and 10 other staphylococcal genes (Table S9). The down-regulated DEGs consisted of 10 genes in the mutual prophage and 13 MRSA genes (Table S10).

### 3.6. Global KEGG Analyses of DEG Profiles

**Figure 5:**
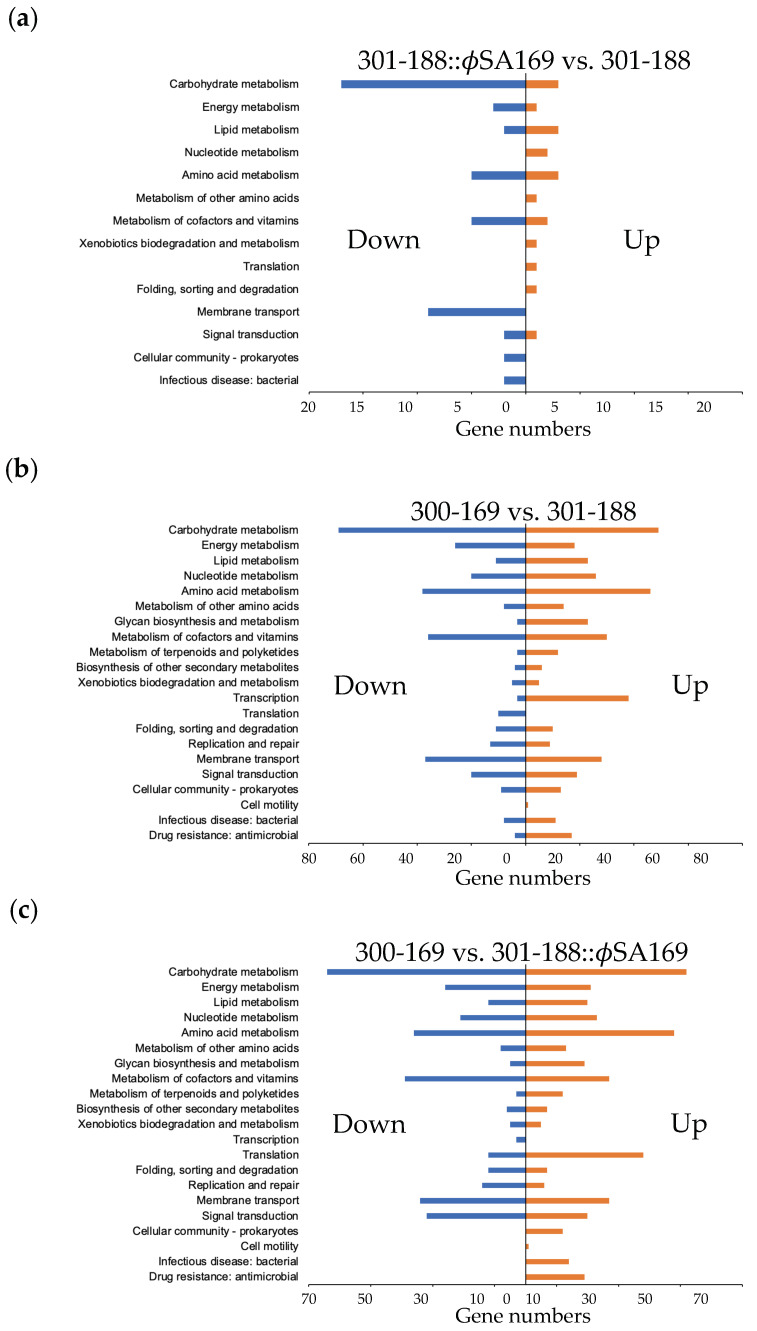
KEGG analysis of the DEGs from (**a**) 301-188::ϕSA169 vs. 301-188; (**b**) 300-169 vs. 301-188; (**c**) 300-169 vs. 301-188::ϕSA169. 301-188::ϕSA169 vs. 301-188 had significantly more DEGs down-regulated than the DEGs up-regulated, and most DEGs were related to metabolic pathways. 300-169 vs. 301-188 and 300-169 vs. 301-188::ϕSA169 had similar KEGG analysis profiles; most of the DEGs were involved in metabolism.

To understand the gene functions and pathways associated with the persistent outcomes, we classified the DEGs using the KEGG pathways mapper tool (Figure 5). In 301-188::ϕSA169, a significant number of genes were down-regulated compared to 301-188 (e.g., carbohydrate metabolism and membrane transport; Figure 5a). In 300-169, genes involved in carbohydrate and amino acids metabolisms, metabolism of cofactors and vitamins, and membrane transport were mainly differentially expressed vs. 301-188 (Figure 5b). Some pathways were found up-regulated in 300-169 vs. 301-188 (e.g., glycan biosynthesis and metabolism, transcription, and drug resistance; Figure 5b). The KEGG analysis profile of 300-169 vs. 301-188::ϕSA169 (Figure 5c) was similar to 300-169 vs. 301-188 (Figure 5b), suggesting the significant differences may be due to the different genetic backgrounds.

### 3.7. ϕSA169-Specific KEGG Analyses

**Figure 6:**
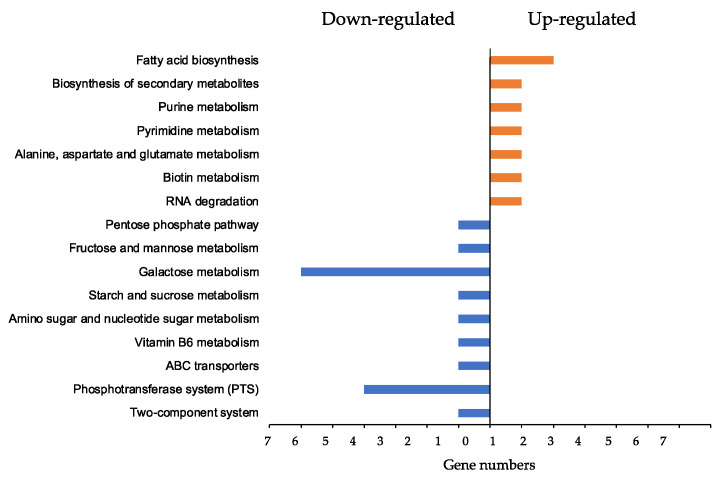
KEGG analysis of the DEGs impacted by ϕSA169. Fatty acid biosynthesis had the most genes up-regulated, compared to the other pathways, while galactose metabolism and phosphotransferase system (PTS) were the pathways that had most genes down-regulated.

The overlapping DEGs of 301-188::ϕSA169 vs. 301-188 and 300-169 vs. 301-188 might represent the genes regulated explicitly by ϕSA169 (Figure 4). The KEGG profile of the overlapping DEGs indicated that most of these genes were involved in metabolic pathways (Figure 6). For instance, the DEGs of fatty acid biosynthesis (*fabFH*), purine metabolism (*purA*), and RNA degradation (AS94_08925) were up-regulated by ϕSA169. Among the down-regulated DEGs by ϕSA169, many of them belonged to galactose metabolism (*lacABCDEF*) and phosphotransferase system (PTS) (*treP*, *pfkB*) (Figure 6).

### 3.8. Verification of the Selected DEGs

**Figure 7:**
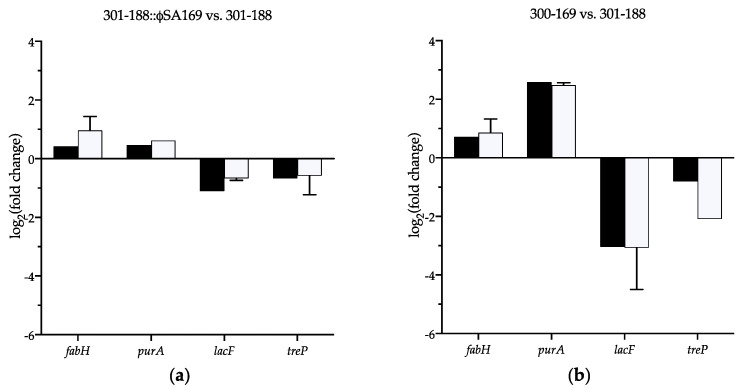
Verification of selected genes *fabH*, *purA*, *lacF,* and *treP* using qRT-PCR. The fold changes of the selected genes determined by qRT-PCR (white bars) were consistent with the values obtained by RNA-seq (black bars) in both comparisons of (**a**) 301-188::ϕSA169 vs. 301-188 and (**b**) 300-169 vs. 301-188.

DEGs that were up-/down-regulated in both comparisons 301-188::ϕSA169 vs. 301-188 and 300-169 vs. 301-188 were thought to be significantly impacted by ϕSA169. The expression of four DEGs (*fabH*, *purA*, *lacF*, and *treP*) involved in different KEGG pathways was selected to verify the RNA-seq results using qRT-PCR. Primers for the selected genes are listed in Table S11. Genes *fabH*/*purA* and *lacF*/*treP* represented significantly up- and down-regulated DEGs by ϕSA169, respectively. The fold changes of the four genes determined by the qRT-PCR were similar to the results obtained in the RNA-seq assays (Figure 7).

## 4. Discussion

Many phages carry virulence factors that significantly contribute to genome variation, pathogenesis, and antibiotic resistance in *S. aureus* [7,19,20]. Despite the obvious importance of phages, studies on the interactions between phage and MRSA persistent outcome are limited. Recently, we demonstrated that the lysogenization of clinical RB 301-188 strain with phage ϕSA169 resulted in persistent phenotypes in vitro and in an experimental endocarditis model [11]. Thus, the current study was designed to determine the impact of ϕSA169 on genetic factors that may contribute to persistent MRSA endovascular infections.

The RNA-seq results revealed that MRSA host genes up-regulated by ϕSA169 were mainly involved in fatty acid biosynthesis (*fabF* and *fabH*), purine (*purA*), pyrimidine (AS94_12220), and RNA degradation (AS94_08925). Both *fabF* and *fabH* encode essential enzymes for fatty acid biosynthesis in many pathogens, including *S. aureus* [21]. Fatty acids are crucial hydrophobic components of membrane lipids and are important metabolic energy sources in bacteria [22]. It has been reported that defected unsaturated fatty acid biosynthesis in *Streptococcus mutans* results in attenuated virulence (e.g., less transmissible, less carious lesions) in a rodent model of dental caries [23]. In addition, fatty acid biosynthesis contributes to virulence in Group B *Streptococcus* (GBS) [24]. Importantly, fatty acid biosynthesis pathway inhibition has been investigated as a possible antimicrobial agent in bacteria [25]. In the current study, significantly higher expressions of *fabF* and *fabH* were observed in the ϕSA169-carrying strains, which may result in survival advantage and consequent persistence.

As a member of *pur* regulon, *purA* encodes the enzyme that catalyzes the conversion of inosine-5-phosphate (IMP) to adenylosuccinate [26]. We and others have previously shown that purine biosynthesis promotes virulence and persistence in *S. aureus* [14,26,27,28]. For instance, the inactivation of *purA* causes the lower expression of a broad spectrum of genes (e.g., energy production and conversion) and attenuates the ability of *S. aureus* to cause kidney infection in mice [27]. Li et al. reported that higher purine biosynthesis production correlates with persistent outcomes in an experimental MRSA endocarditis model [14]. In addition, several studies demonstrated that the inactivation of purine biosynthesis repressor, *purR*, leads to a greater amount of secreted virulence factors and hypervirulence in the murine model of *S. aureus* bacteremia model [26,28]. In the current study, the purine biosynthesis gene, *purA*, was found to be significantly up-regulated by ϕSA169. Therefore, ϕSA169-related higher *purA* expression might contribute to the persistent outcomes we observed in our recent study [11].

It is also interesting that ϕSA169 significantly down-regulated several genes related to the galactose metabolism. Galactose is a common monosaccharide used by organisms [29]. *S. aureus* employs lac operon to import and metabolize galactose [30]. In a previous study, the down-regulation of *lac* operon was observed in a *rpoB* (A621E) mutant *S. aureus* strain that had decreased susceptibility to vancomycin compared to the parental strain [31]. Therefore, down-regulated lac operon in the ϕSA169-carrying strains might contribute to the persistent outcomes with VAN treatment in vivo [11]. However, more research into galactose metabolism and its role in pathogenesis and persistence in *S. aureus* is needed. The RNA-seq displayed down-regulation of the phosphotransferase system (PTS) by ϕSA169. It has been demonstrated that the PTS plays an important role in carbohydrate transport, and the regulation of sugar utilization genes, which further contributes to overall metabolic efficiency in Gram-positive bacteria [32,33]. Gera et al. reported that deleting *ptsI* that encodes cytosolic enzyme I (EI) (Δ*ptsI*) in group A *Streptococcus* (GAS) strains resulted in a hypervirulent phenotype compared to their respective wild-type strains (e.g., significantly increased skin lesion severity and size) in a murine model of disseminating skin and soft tissue infection [33]. Thus, PTS appears to reduce the virulence of GAS skin infection. However, a conflict phenotype of interrupted *ptsI* in *S. aureus* was reported with an attenuated virulence compared to its wild-type strain in a systemic infection model [34]. We suspect this discrepancy is possibly due to (i) the PTS regulation of virulence factors in GAS vs. *S. aureus* and (ii) the animal models used (skin and soft tissue infection vs. systemic infection). Importantly, galactose is one of the carbohydrates that utilizes PTS [35]. Thus, down-regulated PTS in ϕSA169-carrying strains might be correlated with the lower expression of galactose metabolism genes. Detailed studies are needed to define the specific role of PTS, and the interaction between PTS and galactose, in persistent MRSA endovascular infection.

In this study, we also observed that some genes within the mutual prophage in both 300-169 and 301-188 strains were negatively impacted by ϕSA169, which suggested that the mutual prophage genes might be another ϕSA169-derived genetic factor that participated in the PB outcomes. It has been reported that the pathogenesis of *S. aureus* Newman requires the participation of its all four prophages [7]. Thus, multiple prophages might have combined effects on virulence and pathogenesis in *S. aureus*. Therefore, ϕSA169 might contribute to the PB outcomes by mediating the gene expression of the mutual prophage.

Besides the impact of ϕSA169 on genetic factors in the MRSA host genes and the mutual prophage, the different genetic backgrounds between 300-169 and 301-188 strains might also play a role in the persistent outcomes (Figure S1). We have previously demonstrated that key global regulators were differently expressed in 300-169 and 301-188 [11,14]. These differences may impact downstream virulence factors, subsequently contributing to the persistent outcome.

We recognize that there were some significant limitations in the current study. For instance, we only studied a PB 300-169 WT (300-169) containing ϕSA169, RB 301-188 WT (301-188) in the absence of ϕSA169, and 301-188 WT with ϕSA169 lysogenization (301-188::ϕSA169) in the current and previous research [11]. It would be important to verify the genetic impact of ϕSA169 using ϕSA169 deletion in the PB 300-169 strain background. In addition, it would be interesting to study the combinational effect of VAN with ϕSA169 on the MRSA host and ϕSA169 genes, which may demonstrate how ϕSA169 mediates the response to VAN treatment in the IE model [11]. Therefore, further investigations are needed to address these limitations.

## 5. Conclusions

In this study, we explored the impact of prophage ϕSA169 on genetic factors, which might play an essential role in MRSA-persistent endovascular infection. The results highlighted that ϕSA169 contributed to PB outcomes mainly through mediating metabolisms, especially the up-regulation of fatty acid biosynthesis and down-regulation of galactose metabolism and PTS. In addition, the mutual prophage in both 300-169 and 301-188 strains and different genetic backgrounds of these two strains might also be the genetic factors that contribute to the PB outcomes.

## Figures and Tables

**Table 1:** A comparison of differentially expressed genes (DEGs) between the study strains.

	No. of Total DEGs	No. of Up-Regulated DEGs	No. of Down-Regulated DEGs
301-188::ϕSA169 vs. 301-188	153	77	76
300-169 vs. 301-188	1299	666	633
300-169 vs. 301-188::ϕSA169	1250	637	613

**Table 2:** DEGs up-regulated by ϕSA169.

		Log_2_(Fold Change)		
Gene Locus	Group	301-188::ϕSA169 vs. 301-188	300-169 vs. 301-188	Products
AS94_02505	host genes	0.93	0.71	MerR family transcriptional regulator
AS94_04115		0.42	0.72	*fabH*, 3-oxoacyl-ACP synthase
AS94_04120		0.58	1.09	*fabF*, 3-oxoacyl-ACP synthase
AS94_04780		0.51	1.29	amino acid permease
AS94_05160		0.69	0.86	Na/Pi cotransporter
AS94_05540		0.44	1.64	glycine/betaine ABC transporter permease
AS94_05860		0.56	0.68	guanine permease
AS94_06080		0.66	1.52	hypothetical protein
AS94_06090		0.50	0.70	octopine dehydrogenase
AS94_06310		0.48	1.09	sodium:glutamate symporter
AS94_07385		0.71	1.00	transglycosylase
AS94_08925		0.50	0.51	DEAD/DEAH box helicase
AS94_11275		0.47	2.59	*purA*, adenylosuccinate synthetase
AS94_11985		0.42	0.93	multidrug ABC transporter ATP-binding protein
AS94_12030		0.37	0.70	general stress protein
AS94_12410		0.42	1.23	ribonuclease BN
AS94_12040	ϕSA169 genes	7.11	8.46	hypothetical protein
AS94_12045		12.65	12.89	XRE family transcriptional regulator
AS94_12050		10.26	11.22	hypothetical protein
AS94_12055		9.94	10.84	autolysin
AS94_12060		7.65	8.65	holin
AS94_12065		8.70	9.21	hypothetical protein
AS94_12070		10.97	11.98	tail protein
AS94_12075		11.73	12.79	cell wall hydrolase
AS94_12080		8.84	9.51	hypothetical protein
AS94_12090		7.30	8.65	hypothetical protein
AS94_12095		11.29	12.19	hypothetical protein
AS94_12100		11.76	12.57	minor structural protein
AS94_12105		11.63	12.72	peptidase
AS94_12110		10.25	11.46	phage tail protein
AS94_12115		13.07	13.92	membrane protein
AS94_12120		8.67	9.90	ϕ11_gp41
AS94_12125		9.20	10.46	hypothetical protein
AS94_12130		11.18	12.24	tail protein
AS94_12135		8.78	9.81	ϕ11_gp38
AS94_12140		7.89	9.24	hypothetical protein
AS94_12145		7.15	8.87	hypothetical protein
AS94_12150		8.69	10.26	phage head-tail adapter protein
AS94_12155		6.85	8.35	ϕ11_gp35
AS94_12160		11.81	12.91	hypothetical protein
AS94_12165		11.97	13.41	phage capsid protein
AS94_12170		6.29	7.51	hypothetical protein
AS94_12175		11.21	12.22	phage head morphogenesis protein
AS94_12180		11.61	12.56	phage portal protein
AS94_12185		10.89	12.01	hypothetical protein
AS94_12190		10.14	11.07	terminase
AS94_12195		10.04	9.18	transcriptional regulator
AS94_12210		8.22	7.00	hypothetical protein
AS94_12215		7.15	5.65	hypothetical protein
AS94_12220		9.85	9.62	*dut*, dUTP pyrophosphatase
AS94_12230		6.69	6.41	hypothetical protein
AS94_12240		8.84	8.52	hypothetical protein
AS94_12270		5.76	6.56	DNA N-6-adenine-methyltransferase
AS94_12295		9.66	9.80	hypothetical protein
AS94_12320		5.72	5.64	hypothetical protein
AS94_12325		10.13	10.26	hypothetical protein
AS94_12330		6.36	6.05	hypothetical protein
AS94_12340		6.27	7.58	hypothetical protein
AS94_12345		11.88	12.34	BRO-like protein
AS94_12350		10.47	10.70	hypothetical protein
AS94_12355		8.09	8.50	XRE family transcriptional regulator
AS94_12360		12.88	12.46	transcriptional regulator
AS94_12365		8.86	8.90	ϕ80α_gp05
AS94_12370		12.98	13.02	repressor
AS94_12375		10.38	10.92	integrase

**Table 3:** DEGs down-regulated by ϕSA169.

		Log_2_(Fold Change)		
Gene Locus	Group	301-188::ϕSA169 vs. 301-188	300-169 vs. 301-188	Products
AS94_03800	host genes	−0.82	−2.38	cysteine protease
AS94_04675		−0.39	−0.50	*sdrD*, hydrolase
AS94_05575		−0.74	−2.33	*lacE*, PTS lactose transporter subunit IIBC
AS94_05580		−1.11	−3.04	*lacF*, PTS lactose transporter subunit IIA
AS94_05585		−0.81	−2.57	*lacD*, tagatose-bisphosphate aldolase
AS94_05590		−0.85	−2.37	*lacC*, tagatose-6-phosphate kinase
AS94_05595		−1.09	−2.30	*lacB*, galactose-6-phosphate isomerase
AS94_05600		−0.76	−2.60	*lacA*, galactose-6-phosphate isomerase
AS94_06915		−0.54	−0.84	*nikA*, nickel ABC transporter substrate-binding protein
AS94_07070		−0.39	−0.70	*gntk*, gluconokinase
AS94_08235		−0.48	−0.83	*pfkB*, phosphofructokinase
AS94_08280		−0.48	−0.40	hypothetical protein
AS94_09210		−0.58	−1.88	general stress protein
AS94_10090		−0.86	−0.78	murein hydrolase regulator *lrgA*, LrgA
AS94_10365		−0.78	−1.53	sialic acid transporter
AS94_10370		−0.84	−1.75	*nanA*, N-acetylneuraminate lyase
AS94_10375		−0.37	−0.87	N-acetylmannosamine kinase
AS94_11050		−0.67	−0.81	*treP*, PTS ascorbate transporter subunit IIA
AS94_11645		−0.38	−0.35	pyridoxal biosynthesis protein
AS94_12380		−0.90	−0.88	hypothetical protein
AS94_12875		−0.43	−0.72	*hld*, delta-hemolysin
AS94_13070	the mutual prophage in 300-169 and 301-188	−1.47	−2.34	autolysin
AS94_13075		−1.69	−3.25	holin
AS94_13080		−1.68	−2.51	hypothetical protein
AS94_13090		−2.18	−2.40	hypothetical protein
AS94_13095		−1.70	−2.35	hypothetical protein
AS94_13100		−1.53	−2.07	minor structural protein
AS94_13110		−1.72	−2.18	peptidase
AS94_13115		−1.90	−2.25	holin
AS94_13120		−1.62	−2.11	tail protein
AS94_13130		−2.75	−2.80	hypothetical protein
AS94_13135		−1.42	−2.07	tail protein
AS94_13140		−2.01	−2.12	tail protein
AS94_13150		−1.85	−1.72	hypothetical protein
AS94_13160		−2.00	−2.24	hypothetical protein
AS94_13165		−1.93	−2.10	phage capsid protein
AS94_13170		−2.19	−2.07	ATP-dependent Clp protease ClpP
AS94_13175		−1.61	−1.79	portal protein
AS94_13180		−1.71	−1.80	terminase
AS94_13185		−1.74	−1.59	terminase
AS94_13190		−2.02	−1.49	HNH endonuclease
AS94_13195		−0.73	−1.93	transcriptional regulator
AS94_13200		−0.89	−1.56	helicase
AS94_13205		−0.75	−1.44	hypothetical protein
AS94_13355		−0.46	−0.74	antirepressor

## Data Availability

Not applicable.

## Supplementary Materials

The following supporting information can be downloaded at: https://www.mdpi.com/article/10.3390/genes13091527/s1, Table S1: Up-regulated DEGs in 301-188::ϕSA169 vs. 301-188; Table S2: Down-regulated DEGs in 301-188::ϕSA169 vs. 301-188; Table S3: Up-regulated DEGs in 300-169 vs. 301-188; Table S4: Down-regulated DEGs in 300-169 vs. 301-188; Table S5: Up-regulated DEGs in 300-169 vs. 301-188::ϕSA169; Table S6: Down-regulated DEGs in 300-169 vs. 301-188::ϕSA169; Table S7: Up-regulated DEGs in both 300-169 vs. 301-188 and 300-169 vs. 301-188::ϕSA169; Table S8: Down-regulated DEGs in both 300-169 vs. 301-188 and 300-169 vs. 301-188::ϕSA169; Table S9: DEGs up-regulated by both ϕSA169 and MRSA genetic backgrounds; Table S10: DEGs down-regulated by both ϕSA169 and MRSA genetic backgrounds; Table S11: Primers for qRT-PCR confirmation; Figure S1: KEGG analysis of the DEGs impacted by the distinct genetic backgrounds of 300-169 and 301-188.
