# Supplementary material for: Transcriptome Analyses of Prophage in Mediating Persistent Methicillin-Resistant Staphylococcus aureus Endovascular Infection
Source: Genes (Basel). 2022 Aug 25;13(9):1527. doi: 10.3390/genes13091527 (PMC9498598; doi:10.3390/genes13091527)
Supplement: Supplementary file 1 [file genes-13-01527-s001.zip › Table S1.pdf]

Table S1. Up-regulated DEGs in 301-188::ϕSA169 vs. 301-188

locus	group	product	log_2(fold change)	*p* value	*p* adj
AS94_02500	host genes	glutamine synthetase	0.556	0.000	0.002
AS94_02505		MerR family transcriptional regulator	0.933	0.000	0.000
AS94_02590		2-oxoacid ferredoxin oxidoreductase subunit beta	0.507	0.001	0.022
AS94_02930		50S ribosomal protein L28	0.362	0.002	0.035
AS94_04115		3-oxoacyl-ACP synthase	0.423	0.000	0.008
AS94_04120		3-oxoacyl-ACP synthase	0.577	0.000	0.005
AS94_04780		amino acid permease	0.507	0.000	0.001
AS94_05160		Na/Pi cotransporter	0.690	0.000	0.003
AS94_05540		glycine/betaine ABC transporter permease	0.439	0.001	0.020
AS94_05860		guanine permease	0.557	0.001	0.022
AS94_06080		hypothetical protein	0.661	0.000	0.000
AS94_06090		octopine dehydrogenase	0.496	0.001	0.015
AS94_06100		hypothetical protein	0.508	0.001	0.024
AS94_06130		hypothetical protein	0.502	0.001	0.011
AS94_06265		lysostaphin resistance protein A	0.492	0.000	0.004
AS94_06310		sodium:glutamate symporter	0.479	0.002	0.039
AS94_07090		hypothetical protein	0.386	0.002	0.038
AS94_07245		holin	0.604	0.001	0.011
AS94_07385		transglycosylase	0.712	0.000	0.000
AS94_07830		alpha/beta hydrolase	0.557	0.000	0.001
AS94_08370		lipid kinase	0.447	0.000	0.002
AS94_08925		DEAD/DEAH box helicase	0.502	0.000	0.002
AS94_09125		aldehyde dehydrogenase	0.440	0.000	0.008
AS94_10160		glycyl-glycine endopeptidase	0.540	0.000	0.009
AS94_11275		adenylosuccinate synthetase	0.471	0.003	0.047
AS94_11985		multidrug ABC transporter ATP-binding protein	0.417	0.001	0.011
AS94_12030		general stress protein	0.375	0.001	0.012
AS94_12410		ribonuclease BN	0.420	0.003	0.044
AS94_12040	*ϕ*SA169	hypothetical protein	7.107	0.000	0.000
AS94_12045		XRE family transcriptional regulator	12.651	0.000	0.000
AS94_12050		hypothetical protein	10.258	0.000	0.000
AS94_12055		autolysin	9.937	0.000	0.000

AS94_12060	holin	7.646	0.000	0.000
AS94_12065	hypothetical protein	8.700	0.000	0.000
AS94_12070	tail protein	10.966	0.000	0.000
AS94_12075	cell wall hydrolase	11.731	0.000	0.000
AS94_12080	hypothetical protein	8.839	0.000	0.000
AS94_12090	hypothetical protein	7.301	0.000	0.000
AS94_12095	hypothetical protein	11.292	0.000	0.000
AS94_12100	minor structural protein	11.761	0.000	0.000
AS94_12105	peptidase	11.625	0.000	0.000
AS94_12110	phage tail protein	10.251	0.000	0.000
AS94_12115	membrane protein	13.073	0.000	0.000
AS94_12120	phi 11	8.667	0.000	0.000
AS94_12125	hypothetical protein	9.203	0.000	0.000
AS94_12130	tail protein	11.175	0.000	0.000
AS94_12135	phi 11	8.782	0.000	0.000
AS94_12140	hypothetical protein	7.889	0.000	0.000
AS94_12145	hypothetical protein	7.152	0.000	0.000
AS94_12150	phage head-tail adapter protein	8.690	0.000	0.000
AS94_12155	phi 11	6.853	0.000	0.000
AS94_12160	hypothetical protein	11.809	0.000	0.000
AS94_12165	phage capsid protein	11.968	0.000	0.000
AS94_12170	hypothetical protein	6.290	0.000	0.003
AS94_12175	phage head morphogenesis protein	11.205	0.000	0.000
AS94_12180	phage portal protein	11.607	0.000	0.000
AS94_12185	hypothetical protein	10.893	0.000	0.000
AS94_12190	terminase	10.141	0.000	0.000
AS94_12195	transcriptional regulator	10.043	0.000	0.000
AS94_12210	hypothetical protein	8.216	0.000	0.000
AS94_12215	hypothetical protein	7.154	0.000	0.001
AS94_12220	dUTP pyrophosphatase	9.847	0.000	0.000
AS94_12230	hypothetical protein	6.690	0.000	0.000
AS94_12240	hypothetical protein	8.842	0.000	0.000
AS94_12270	DNA N-6-adenine-methyltransferase	5.757	0.000	0.000
AS94_12295	hypothetical protein	9.662	0.000	0.000

AS94_12320	hypothetical protein	5.724	0.002	0.030
AS94_12325	hypothetical protein	10.130	0.000	0.000
AS94_12330	hypothetical protein	6.362	0.000	0.005
AS94_12340	hypothetical protein	6.265	0.000	0.003
AS94_12345	BRO-like protein	11.879	0.000	0.000
AS94_12350	hypothetical protein	10.468	0.000	0.000
AS94_12355	XRE family transcriptional regulator	8.090	0.000	0.000
AS94_12360	transcriptional regulator	12.883	0.000	0.000
AS94_12365	hypothetical protein	8.858	0.000	0.000
AS94_12370	repressor	12.980	0.000	0.000
AS94_12375	integrase	10.382	0.000	0.000
