# Supplementary material for: Transcriptome Analyses of Prophage in Mediating Persistent Methicillin-Resistant Staphylococcus aureus Endovascular Infection
Source: Genes (Basel). 2022 Aug 25;13(9):1527. doi: 10.3390/genes13091527 (PMC9498598; doi:10.3390/genes13091527)
Supplement: Supplementary file 1 [file genes-13-01527-s001.zip › Table S10.pdf]

Table S10. DEGs down-regulated by both ϕSA169 and MRSA genetic backgrounds

locus	gene	group	product	301-188::ϕSA169 vs. 301-188			300-169 vs. 301-188			300-169 vs. 301-188::ϕSA169		
				log_2(fold change)	*p* value	*p* adj	log_2(fold change)	*p* value	*p* adj	log_2(fold change)	*p* value	*p* adj
AS94_03800		host genes	cysteine protease	-0.816	0.001	0.014	-2.378	0.000	0.000	-1.563	0.000	0.000
AS94_05575			PTS lactose transporter subunit IIBC	-0.742	0.000	0.000	-2.334	0.000	0.000	-1.592	0.000	0.000
AS94_05580	*lacF*		PTS lactose transporter subunit IIA	-1.109	0.002	0.029	-3.043	0.000	0.000	-1.933	0.000	0.000
AS94_05585	*lacD*		tagatose-bisphosphate aldolase	-0.807	0.000	0.000	-2.569	0.000	0.000	-1.762	0.000	0.000
AS94_05590	*lacC*		tagatose-6-phosphate kinase	-0.855	0.000	0.000	-2.374	0.000	0.000	-1.519	0.000	0.000
AS94_05595	*lacB*		galactose-6-phosphate isomerase	-1.089	0.000	0.000	-2.300	0.000	0.000	-1.210	0.000	0.000
AS94_05600	*lacA*		galactose-6-phosphate isomerase	-0.759	0.001	0.011	-2.599	0.000	0.000	-1.840	0.000	0.000
AS94_07070	*gntK*		gluconokinase	-0.387	0.001	0.011	-0.704	0.000	0.000	-0.317	0.005	0.012
AS94_09210			general stress protein	-0.585	0.000	0.002	-1.882	0.000	0.000	-1.297	0.000	0.000
AS94_10365			sialic acid transporter	-0.784	0.000	0.000	-1.527	0.000	0.000	-0.743	0.000	0.000
AS94_10370			N-acetylneuraminate lyase	-0.838	0.000	0.000	-1.747	0.000	0.000	-0.910	0.000	0.000
AS94_10375			N-acetylmannosamine kinase	-0.368	0.003	0.047	-0.868	0.000	0.000	-0.500	0.000	0.000
AS94_12875	*hld*		delta-hemolysin	-0.429	0.000	0.001	-0.725	0.000	0.000	-0.296	0.004	0.010
AS94_13070	*nanA*	mutual prophage	autolysin	-1.467	0.000	0.000	-2.341	0.000	0.000	-0.873	0.000	0.000
AS94_13075			holin	-1.693	0.000	0.000	-3.249	0.000	0.000	-1.556	0.000	0.000
AS94_13095			hypothetical protein	-1.697	0.000	0.000	-2.348	0.000	0.000	-0.651	0.002	0.006
AS94_13100			minor structural protein	-1.525	0.000	0.000	-2.067	0.000	0.000	-0.542	0.001	0.003
AS94_13110			peptidase	-1.716	0.000	0.000	-2.176	0.000	0.000	-0.459	0.021	0.044
AS94_13120			tail protein	-1.623	0.000	0.000	-2.112	0.000	0.000	-0.489	0.000	0.000
AS94_13135			tail protein	-1.424	0.000	0.000	-2.071	0.000	0.000	-0.647	0.000	0.001
AS94_13195			transcriptional regulator	-0.735	0.000	0.000	-1.929	0.000	0.000	-1.194	0.000	0.000
AS94_13200			helicase	-0.895	0.000	0.000	-1.557	0.000	0.000	-0.662	0.002	0.006
AS94_13205			hypothetical protein	-0.750	0.000	0.002	-1.435	0.000	0.000	-0.686	0.001	0.002
