# Supplementary material for: Transcriptome Analyses of Prophage in Mediating Persistent Methicillin-Resistant Staphylococcus aureus Endovascular Infection
Source: Genes (Basel). 2022 Aug 25;13(9):1527. doi: 10.3390/genes13091527 (PMC9498598; doi:10.3390/genes13091527)
Supplement: Supplementary file 1 [file genes-13-01527-s001.zip › Table S11.pdf]

Table S11. Primers for qRT-PCR confirmation

Gene	Primer sequence (5′-3′)
*fabH*_F	TCGCTGACGCTGGTATTCAG
*fabH*_R	GCCCGTCCCTAAACGTTCTT
*purA*_F	TATCGCGCGTTTTTCAGGTG
*purA*_R	ACTCCGTTACCGATTACCGC
*lacF*_F	GTTGCATTTGCAGGGGATGC
*lacF*_R	CCAACAGGCTTGTTTGTGCT
*treP*_F	TCAAGGGGCAGTTCAAAGCA
*treP*_R	TTTTTGTGCAGCGGCTTGTT
