# Supplementary material for: Transcriptome Analyses of Prophage in Mediating Persistent Methicillin-Resistant Staphylococcus aureus Endovascular Infection
Source: Genes (Basel). 2022 Aug 25;13(9):1527. doi: 10.3390/genes13091527 (PMC9498598; doi:10.3390/genes13091527)
Supplement: Supplementary file 1 [file genes-13-01527-s001.zip › Table S2.pdf]

Table S2. Down-regulated DEGs in 301-188::ϕSA169 vs. 301-188

locus	group	product	log_2(fold change)	*p* value	*p* adj
AS94_00025	host genes	calcium-binding protein	-0.470	0.000	0.010
AS94_00060		phosphoenolpyruvate carboxykinase	-0.471	0.000	0.002
AS94_00170		proline dehydrogenase	-0.752	0.000	0.000
AS94_00315		histone deacetylase	-0.566	0.000	0.009
AS94_00325		acetyl-CoA synthetase	-0.495	0.000	0.007
AS94_00560		glyceraldehyde-3-phosphate dehydrogenase	-0.469	0.000	0.004
AS94_00630		glutamyl-tRNA reductase	-0.339	0.001	0.014
AS94_01855		2-oxoglutarate dehydrogenase E1	-0.348	0.001	0.020
AS94_03245		leukocidin/Hemolysin toxin family protein	-0.588	0.000	0.000
AS94_03460		membrane protein	-0.466	0.000	0.010
AS94_03465		protoheme IX farnesyltransferase	-0.429	0.000	0.007
AS94_03470		heme A synthase	-0.406	0.001	0.022
AS94_03800		cysteine protease	-0.816	0.001	0.014
AS94_04145		ATP-dependent Clp protease ATP-binding protein	-0.452	0.000	0.004
AS94_04240		ornithine-oxoacid aminotransferase	-0.328	0.002	0.040
AS94_04635		enterotoxin	-0.464	0.000	0.001
AS94_04675		hydrolase	-0.390	0.001	0.027
AS94_05575		PTS lactose transporter subunit IIBC	-0.742	0.000	0.000
AS94_05580		PTS lactose transporter subunit IIA	-1.109	0.002	0.029
AS94_05585		tagatose-bisphosphate aldolase	-0.807	0.000	0.000
AS94_05590		tagatose-6-phosphate kinase	-0.855	0.000	0.000
AS94_05595		galactose-6-phosphate isomerase	-1.089	0.000	0.000
AS94_05600		galactose-6-phosphate isomerase	-0.759	0.001	0.011
AS94_06915		nickel ABC transporter substrate-binding protein	-0.541	0.000	0.010
AS94_07070		gluconokinase	-0.387	0.001	0.011
AS94_07310		1-pyrroline-5-carboxylate dehydrogenase	-0.377	0.001	0.011
AS94_07320		hypothetical protein	-0.692	0.000	0.000
AS94_07900		manganese ABC transporter substrate-binding protein	-0.644	0.000	0.000
AS94_07905		membrane protein	-0.688	0.000	0.000
AS94_07910		phosphonate ABC transporter ATP-binding protein	-0.754	0.000	0.000

AS94_08235		phosphofructokinase	-0.480	0.003	0.047
AS94_08280		hypothetical protein	-0.481	0.001	0.012
AS94_09210		general stress protein	-0.585	0.000	0.002
AS94_09755		cellobiose operon outer surface protein	-0.655	0.000	0.001
AS94_09760		N-acetylmuramic acid-6-phosphate etherase	-0.627	0.000	0.001
AS94_09765		permease	-0.756	0.000	0.000
AS94_09770		RpiR family transcriptional regulator	-0.666	0.000	0.001
AS94_09985		lactate dehydrogenase	-0.464	0.000	0.002
AS94_10090		murein hydrolase regulator LrgA	-0.855	0.000	0.001
AS94_10365		sialic acid transporter	-0.784	0.000	0.000
AS94_10370		N-acetylneuraminate lyase	-0.838	0.000	0.000
AS94_10375		N-acetylmannosamine kinase	-0.368	0.003	0.047
AS94_10395		lipase	-0.441	0.000	0.002
AS94_11050		PTS ascorbate transporter subunit IIA	-0.674	0.000	0.000
AS94_11470		UDP-glucose 4-epimerase	-0.479	0.000	0.003
AS94_11640		glutamine amidotransferase	-0.411	0.001	0.015
AS94_11645		pyridoxal biosynthesis protein	-0.378	0.002	0.030
AS94_11740		lipase	-0.533	0.000	0.001
AS94_12380		hypothetical protein	-0.900	0.000	0.000
AS94_12580		nitric oxide synthase	-0.436	0.001	0.014
AS94_12875		delta-hemolysin	-0.429	0.000	0.001
AS94_13490		peptidyl-prolyl cis-trans isomerase	-0.413	0.001	0.021
AS94_13070	mutual prophage	autolysin	-1.467	0.000	0.000
AS94_13075		holin	-1.693	0.000	0.000
AS94_13080		hypothetical protein	-1.675	0.000	0.000
AS94_13090		hypothetical protein	-2.183	0.000	0.000
AS94_13095		hypothetical protein	-1.697	0.000	0.000
AS94_13100		minor structural protein	-1.525	0.000	0.000
AS94_13110		peptidase	-1.716	0.000	0.000
AS94_13115		holin	-1.895	0.000	0.000
AS94_13120		tail protein	-1.623	0.000	0.000
AS94_13130		hypothetical protein	-2.751	0.000	0.000
AS94_13135		tail protein	-1.424	0.000	0.000
AS94_13140		tail protein	-2.010	0.000	0.000

AS94_13150	hypothetical protein	-1.850	0.000	0.000
AS94_13160	hypothetical protein	-2.001	0.000	0.000
AS94_13165	phage capsid protein	-1.930	0.000	0.000
AS94_13170	ATP-dependent Clp protease ClpP	-2.186	0.000	0.000
AS94_13175	portal protein	-1.607	0.000	0.000
AS94_13180	terminase	-1.715	0.000	0.000
AS94_13185	terminase	-1.743	0.000	0.000
AS94_13190	HNH endonuclease	-2.024	0.000	0.001
AS94_13195	transcriptional regulator	-0.735	0.000	0.000
AS94_13200	helicase	-0.895	0.000	0.000
AS94_13205	hypothetical protein	-0.750	0.000	0.002
AS94_13355	antirepressor	-0.457	0.001	0.017
