# Supplementary material for: Transcriptome Analyses of Prophage in Mediating Persistent Methicillin-Resistant Staphylococcus aureus Endovascular Infection
Source: Genes (Basel). 2022 Aug 25;13(9):1527. doi: 10.3390/genes13091527 (PMC9498598; doi:10.3390/genes13091527)
Supplement: Supplementary file 1 [file genes-13-01527-s001.zip › Table S3.pdf]

Table S3. Up-regulated DEGs in 300-169 vs. 301-188

locus	gene	group	product	log_2(fold change)	*p* value	*p* adj
AS94_00015			hypothetical protein	0.979	0.000	0.000
AS94_00025			calcium-binding protein	1.265	0.000	0.000
AS94_00035			2-succinylbenzoate--CoA ligase	0.382	0.008	0.017
AS94_00060			phosphoenolpyruvate carboxykinase	0.596	0.000	0.000
AS94_00145			pyridine nucleotide-disulfide oxidoreductase	0.504	0.025	0.049
AS94_00150			diaminohydroxyphosphoribosylamino pyrimidine deaminase	1.209	0.000	0.000
AS94_00155			riboflavin synthase subunit alpha	0.838	0.000	0.000
AS94_00160			GTP cyclohydrolase	0.831	0.000	0.000
AS94_00165			6_7-dimethyl-8-ribityllumazine synthase	0.469	0.016	0.034
AS94_00170			proline dehydrogenase	0.863	0.000	0.000
AS94_00175			lysophospholipase	0.575	0.000	0.000
AS94_00180			MarR family transcriptional regulator	0.666	0.000	0.000
AS94_00185		host genes	rRNA methyltransferase	0.497	0.005	0.011
AS94_00190			hypothetical protein	0.382	0.013	0.028
AS94_00275			hypothetical protein	1.099	0.000	0.000
AS94_00280			tRNA-binding protein	1.274	0.000	0.000
AS94_00285			cell division protein FtsK	1.122	0.000	0.000
AS94_00290			UDP-N-acetylmuramate--alanine ligase	0.808	0.000	0.000
AS94_00295			hypothetical protein	0.999	0.000	0.000
AS94_00300			smooth muscle caldesmon	1.170	0.000	0.000
AS94_00325			acetyl-CoA synthetase	0.516	0.000	0.000
AS94_00330			formate--tetrahydrofolate ligase	0.281	0.017	0.036
AS94_00350			serine protease	1.256	0.000	0.000
AS94_00390			30S ribosomal protein S4	0.617	0.000	0.000
AS94_00410			thiamine biosynthesis protein ThiI	0.416	0.014	0.030
AS94_00430			acetate kinase	0.460	0.000	0.000
AS94_00440			alanine dehydrogenase	0.595	0.000	0.000
AS94_00530			PhoP family transcriptional regulator	0.349	0.024	0.049

AS94_00560	glyceraldehyde-3-phosphate dehydrogenase	0.674	0.000	0.000
AS94_00590	translation initiation factor IF-3	1.200	0.000	0.000
AS94_00595	50S ribosomal protein L35	1.376	0.000	0.000
AS94_00600	50S ribosomal protein L20	1.353	0.000	0.000
AS94_00605	DNA mismatch repair protein MutT	0.476	0.015	0.031
AS94_00615	trigger factor	1.057	0.000	0.000
AS94_00620	ATP-dependent protease	0.740	0.000	0.000
AS94_00625	GTP-binding protein YsxC	0.620	0.002	0.004
AS94_00630	glutamyl-tRNA reductase	0.366	0.000	0.001
AS94_00635	cytochrome C assembly protein	0.707	0.000	0.000
AS94_00640	porphobilinogen deaminase	0.816	0.000	0.000
AS94_00660	aminopeptidase	0.345	0.009	0.021
AS94_00670	valyl-tRNA synthase	0.361	0.001	0.002
AS94_00700	membrane protein	3.845	0.000	0.000
AS94_00705	hypothetical protein	4.743	0.000	0.000
AS94_00710	rod shape-determining protein MreC	0.865	0.000	0.000
AS94_00715	rod shape-determining protein MreD	0.839	0.000	0.000
AS94_00760	queuine tRNA-ribosyltransferase	0.546	0.000	0.000
AS94_00775	single-stranded-DNA exonuclease	0.297	0.011	0.024
AS94_00815	hypothetical protein	0.338	0.007	0.016
AS94_00830	hypothetical protein	1.817	0.000	0.000
AS94_00835	hypothetical protein	0.851	0.012	0.026
AS94_00910	allophanate hydrolase subunit 1	2.559	0.000	0.000
AS94_00915	allophanate hydrolase subunit 2	2.558	0.000	0.000
AS94_00920	acetyl-CoA carboxylase	2.132	0.000	0.000
AS94_00925	acetyl-CoA carboxylase biotin carboxylase subunit	2.006	0.000	0.000
AS94_00930	hypothetical protein	1.816	0.000	0.000
AS94_00935	iron transporter	1.412	0.000	0.000
AS94_00955	S-adenosylhomocysteine nucleosidase	1.082	0.000	0.000
AS94_00960	hypothetical protein	1.271	0.000	0.000
AS94_00965	GTPase	1.316	0.000	0.000

AS94_00970	shikimate 5-dehydrogenase	1.238	0.000	0.000
AS94_00975	RNA-binding protein	1.600	0.016	0.033
AS94_00980	nicotinic acid mononucleotide adenylyltransferase	1.237	0.000	0.000
AS94_00985	HAD family hydrolase	1.203	0.000	0.000
AS94_00990	Iojap family protein	1.158	0.000	0.000
AS94_00995	methyltransferase	1.162	0.000	0.000
AS94_01020	30S ribosomal protein S20	0.813	0.000	0.000
AS94_01035	HrcA family transcriptional regulator	0.382	0.003	0.007
AS94_01040	heat shock protein GrpE	0.332	0.007	0.017
AS94_01050	molecular chaperone DnaJ	0.880	0.000	0.000
AS94_01055	ribosomal protein L11 methyltransferase	1.069	0.000	0.000
AS94_01060	16S rRNA methyltransferase	0.938	0.000	0.000
AS94_01070	30S ribosomal protein S21	0.750	0.000	0.000
AS94_01075	serine protease	0.726	0.000	0.000
AS94_01080	hypothetical protein	0.434	0.000	0.001
AS94_01085	iron transporter	0.476	0.000	0.000
AS94_01120	glycyl-tRNA synthetase	1.455	0.000	0.000
AS94_01145	SAM-dependent methyltransferase	0.715	0.000	0.000
AS94_01150	hypothetical protein	0.445	0.003	0.008
AS94_01190	penicillin-binding protein 3	0.412	0.001	0.002
AS94_01195	50S ribosomal protein L33	0.880	0.000	0.001
AS94_01200	5-formyltetrahydrofolate cyclo-ligase	0.544	0.009	0.019
AS94_01205	membrane protein	0.761	0.000	0.000
AS94_01215	glucokinase	0.731	0.000	0.000
AS94_01220	hypothetical protein	0.830	0.001	0.004
AS94_01225	hydroxyacylglutathione hydrolase	0.715	0.000	0.000
AS94_01305	elongation factor P	0.635	0.000	0.000
AS94_01310	acetyl-CoA carboxylase	0.851	0.000	0.000
AS94_01315	acetyl-CoA carboxylase biotin carboxylase subunit	0.847	0.000	0.000
AS94_01320	hypothetical protein	0.790	0.000	0.000

AS94_01325	transcription antitermination protein NusB	0.587	0.001	0.002
AS94_01330	exodeoxyribonuclease VII large subunit	0.691	0.000	0.000
AS94_01340	geranyltranstransferase	0.603	0.000	0.000
AS94_01345	arginine repressor ArgR	1.174	0.000	0.000
AS94_01350	DNA repair protein RecN	1.368	0.000	0.000
AS94_01355	dihydrolipoamide dehydrogenase	0.664	0.000	0.000
AS94_01375	hypothetical protein	0.830	0.000	0.000
AS94_01380	membrane protein	0.676	0.000	0.000
AS94_01485	PhoP family transcriptional regulator	0.497	0.000	0.000
AS94_01495	hypothetical protein	1.190	0.000	0.000
AS94_01500	hypothetical protein	1.027	0.000	0.000
AS94_01505	hypothetical protein	0.570	0.007	0.016
AS94_01510	Riboflavin transporter RibU	0.378	0.022	0.045
AS94_01520	hypothetical protein	0.418	0.022	0.046
AS94_01525	ATP-dependent DNA helicase RecQ	0.465	0.003	0.007
AS94_01530	peptidoglycan-binding protein LysM	0.259	0.015	0.031
AS94_01535	thioredoxin reductase	0.324	0.010	0.022
AS94_01550	30S ribosomal protein S1	0.414	0.000	0.000
AS94_01585	nucleoside diphosphate kinase	0.787	0.000	0.000
AS94_01655	asparaginyl-tRNA synthase	0.561	0.000	0.000
AS94_01675	transglycosylase	1.190	0.000	0.000
AS94_01680	recombinase RecU	1.159	0.000	0.000
AS94_01685	hypothetical protein	0.913	0.000	0.000
AS94_01690	hypothetical protein	1.081	0.000	0.000
AS94_01695	cell cycle protein GpsB	1.211	0.000	0.000
AS94_01705	RNA methyltransferase	1.002	0.000	0.000
AS94_01710	hypothetical protein	0.892	0.000	0.001
AS94_01715	sulfite reductase subunit alpha	1.860	0.000	0.000
AS94_01720	dynamin family protein	0.696	0.000	0.000
AS94_01725	5'-3' exonuclease	0.386	0.016	0.034
AS94_01785	thymidylate synthase	0.675	0.000	0.000

AS94_01830	UDP-diphospho-muramoylpentapeptide beta-N-acetylglucosaminyltransferase	0.617	0.000	0.000
AS94_01835	phosphatidic acid phosphatase	0.783	0.000	0.000
AS94_01855	2-oxoglutarate dehydrogenase E1	0.400	0.000	0.000
AS94_01860	dihydrolipoamide succinyltransferase	0.266	0.012	0.026
AS94_01890	hypothetical protein	0.760	0.000	0.000
AS94_01895	nitric oxide reductase activation protein NorD	0.444	0.001	0.002
AS94_01900	branched-chain amino acid ABC transporter substrate-binding protein	0.431	0.014	0.030
AS94_01905	tellurite resistance protein TelA	0.509	0.000	0.000
AS94_01910	5-bromo-4-chloroindolyl phosphate hydrolysis protein	0.560	0.001	0.004
AS94_01915	acylphosphatase	0.727	0.000	0.001
AS94_01970	aspartate-semialdehyde dehydrogenase	0.689	0.007	0.017
AS94_01975	aspartate kinase	0.920	0.001	0.003
AS94_01980	ABC transporter ATP-binding protein	0.324	0.009	0.020
AS94_01990	thioredoxine reductase	1.098	0.007	0.016
AS94_02120	LytR family transcriptional regulator	1.111	0.000	0.000
AS94_02130	phosphatidylglycerol lysyltransferase	0.502	0.000	0.000
AS94_02150	sodium:alanine symporter	0.434	0.001	0.003
AS94_02155	DNA topoisomerase IV subunit A	0.486	0.000	0.000
AS94_02160	DNA topoisomerase IV subunit B	0.661	0.000	0.000
AS94_02175	4-hydroxybenzoyl-CoA thioesterase	0.549	0.000	0.000
AS94_02180	aconitate hydratase	0.769	0.000	0.000
AS94_02185	choline transporter	0.898	0.000	0.000
AS94_02205	membrane protein	0.737	0.000	0.000
AS94_02240	secretion protein	0.922	0.000	0.000
AS94_02245	guanosine 5'-monophosphate oxidoreductase	1.619	0.000	0.000
AS94_02275	HAD family hydrolase	0.310	0.014	0.029
AS94_02280	homoserine kinase	0.840	0.000	0.000

AS94_02285	threonine synthase	0.453	0.001	0.004
AS94_02290	homoserine dehydrogenase	0.629	0.000	0.000
AS94_02295	aspartate kinase	1.302	0.000	0.000
AS94_02300	hypothetical protein	0.713	0.000	0.001
AS94_02505	MerR family transcriptional regulator	0.714	0.000	0.000
AS94_02560	DNA mismatch repair protein MutL	0.448	0.000	0.001
AS94_02565	DNA mismatch repair protein MutS	0.538	0.000	0.000
AS94_02620	competence protein CinA	0.452	0.002	0.005
AS94_02635	hypothetical protein	0.311	0.023	0.046
AS94_02645	zinc protease	0.461	0.003	0.007
AS94_02675	30S ribosomal protein S15	0.598	0.000	0.000
AS94_02685	tRNA pseudouridine synthase B	0.498	0.006	0.014
AS94_02700	50S ribosomal protein L7	0.404	0.021	0.043
AS94_02710	transcription elongation factor NusA	0.535	0.000	0.000
AS94_02715	ribosome maturation protein RimP	0.588	0.000	0.000
AS94_02725	prolyl-tRNA synthetase	0.658	0.000	0.000
AS94_02730	zinc metalloprotease	0.744	0.000	0.000
AS94_02735	phosphatidate cytidylyltransferase	0.544	0.000	0.000
AS94_02740	UDP pyrophosphate synthase	0.939	0.000	0.000
AS94_02755	elongation factor Ts	1.044	0.000	0.000
AS94_02760	30S ribosomal protein S2	1.301	0.000	0.000
AS94_02765	transcriptional regulator	0.812	0.000	0.000
AS94_02770	ATP-dependent protease	0.999	0.000	0.000
AS94_02775	ATP-dependent protease	1.002	0.000	0.000
AS94_02780	tyrosine recombinase XerC	1.153	0.000	0.000
AS94_02785	tRNA (uracil-5-)-methyltransferase	0.658	0.000	0.000
AS94_02790	DNA topoisomerase I	0.853	0.000	0.000
AS94_02815	succinyl-CoA synthetase subsunit alpha	0.794	0.000	0.000
AS94_02820	malate--CoA ligase subunit beta	1.079	0.000	0.000
AS94_02845	50S ribosomal protein L19	0.873	0.000	0.000
AS94_02860	30S ribosomal protein S16	0.434	0.005	0.011
AS94_02880	chromosome segregation protein SMC	0.313	0.008	0.017

AS94_02890	acyl carrier protein	0.624	0.000	0.000
AS94_02895	3-ketoacyl-ACP reductase	0.304	0.014	0.029
AS94_02900	malonyl CoA-ACP transacylase	0.332	0.005	0.012
AS94_02905	phosphate acyltransferase	0.676	0.000	0.000
AS94_02910	transcription factor	0.920	0.000	0.000
AS94_02920	hypothetical protein	0.820	0.000	0.000
AS94_02925	hypothetical protein	0.454	0.001	0.004
AS94_02945	GTPase	0.416	0.005	0.013
AS94_02965	16S rRNA methyltransferase	0.343	0.010	0.022
AS94_03015	3-demethylubiquinone-9 3-methyltransferase	0.446	0.004	0.009
AS94_03025	orotate phosphoribosyltransferase	1.225	0.000	0.000
AS94_03030	orotidine 5'-phosphate decarboxylase	1.430	0.000	0.000
AS94_03035	carbamoyl phosphate synthase large subunit	1.017	0.000	0.000
AS94_03040	carbamoyl phosphate synthase small subunit	0.724	0.001	0.002
AS94_03045	dihydroorotase	0.553	0.002	0.006
AS94_03055	uracil transporter	0.422	0.025	0.050
AS94_03065	ribosomal large subunit pseudouridine synthase D	0.311	0.019	0.039
AS94_03115	cell division protein FtsZ	0.639	0.000	0.000
AS94_03120	cell division protein FtsA	0.745	0.000	0.000
AS94_03125	cell division protein FtsQ	0.878	0.000	0.000
AS94_03130	UDP-N-acetylmuramoyl-L-alanyl-D-glutamate synthetase	0.874	0.000	0.000
AS94_03135	phospho-N-acetylmuramoyl-pentapeptide- transferase	0.862	0.000	0.000
AS94_03140	penicillin-binding protein 1	1.055	0.000	0.000
AS94_03145	cell division protein FtsL	0.851	0.000	0.000
AS94_03150	16S rRNA methyltransferase	1.112	0.000	0.000
AS94_03155	cell division protein MraZ	1.223	0.000	0.000
AS94_03165	hypothetical protein	0.912	0.000	0.000

AS94_03175	hypothetical protein	1.653	0.000	0.001
AS94_03180	hypothetical protein	1.556	0.001	0.003
AS94_03195	ethanolamine utilization protein EutQ	0.891	0.000	0.000
AS94_03245	leukocidin/Hemolysin toxin family protein	2.663	0.000	0.000
AS94_03310	succinate dehydrogenase flavoprotein subunit	0.483	0.000	0.001
AS94_03315	succinate dehydrogenase cytochrome B558	0.552	0.000	0.000
AS94_03320	excinuclease ABC subunit C	0.295	0.020	0.041
AS94_03330	DNA mismatch repair protein MutS	0.661	0.000	0.000
AS94_03335	DNA polymerase	0.684	0.000	0.000
AS94_03415	DNA-binding protein	0.334	0.011	0.025
AS94_03445	glycerophosphoryl diester phosphodiesterase	0.404	0.011	0.025
AS94_03460	membrane protein	0.330	0.012	0.025
AS94_03465	protoheme IX farnesyltransferase	0.489	0.000	0.000
AS94_03470	heme A synthase	0.634	0.000	0.000
AS94_03495	GTP-binding protein	0.801	0.000	0.000
AS94_03530	spermidine/putrescine ABC transporter substrate-binding protein	0.732	0.000	0.000
AS94_03535	spermidine/purescine ABC transporter permease	0.712	0.020	0.042
AS94_03550	Cro/Cl family transcriptional regulator	0.777	0.021	0.042
AS94_03600	potassium transporter Trk	0.917	0.000	0.000
AS94_03605	cytochrome D ubiquinol oxidase subunit II	2.267	0.000	0.000
AS94_03610	cytochrome D ubiquinol oxidase subunit I	2.164	0.000	0.000
AS94_03620	phosphoenolpyruvate-protein phosphotransferase	0.790	0.000	0.000
AS94_03625	phosphocarrier protein HPr	0.650	0.000	0.000
AS94_03630	hypothetical protein	0.791	0.000	0.000

AS94_03640	membrane protein	0.810	0.000	0.000
AS94_03665	phosphoribosylamine--glycine ligase	0.763	0.000	0.000
AS94_03670	purine biosynthesis protein purH	1.316	0.000	0.000
AS94_03675	phosphoribosylglycinamide formyltransferase	1.580	0.000	0.000
AS94_03680	phosphoribosylaminoimidazole synthetase	1.747	0.000	0.000
AS94_03685	amidophosphoribosyltransferase	1.476	0.000	0.000
AS94_03690	phosphoribosylformylglycinamidine synthase	1.275	0.000	0.000
AS94_03695	phosphoribosylformylglycinamidine synthase	1.063	0.000	0.001
AS94_03705	phosphoribosylaminoimidazole-succinocarboxamide synthase	0.961	0.007	0.015
AS94_03710	phosphoribosylaminoimidazole carboxylase	1.272	0.000	0.000
AS94_03720	tetrahydrofolate dehydrogenase	0.734	0.000	0.000
AS94_03730	chitinase	0.998	0.000	0.000
AS94_03755	methicillin resistance protein FmtA	1.096	0.000	0.000
AS94_03785	acyltransferase	0.849	0.005	0.012
AS94_03850	ABC transporter substrate-binding protein	1.107	0.011	0.024
AS94_03865	membrane protein	1.708	0.001	0.002
AS94_03885	CAAX amino terminal protease	0.972	0.000	0.000
AS94_03960	MFS transporter	0.330	0.006	0.014
AS94_03965	hypothetical protein	0.692	0.000	0.000
AS94_04055	tryptophanyl-tRNA synthetase	0.433	0.001	0.002
AS94_04085	peptide ABC transporter substrate-binding protein	0.579	0.000	0.000
AS94_04090	peptide ABC transporter ATP-binding protein	0.747	0.000	0.001
AS94_04095	peptide ABC transporter ATP-binding protein	0.695	0.002	0.006

AS94_04100	peptide ABC transporter permease	0.794	0.000	0.000
AS94_04105	peptide ABC transporter permease	0.991	0.000	0.000
AS94_04115	3-oxoacyl-ACP synthase	0.719	0.000	0.000
AS94_04120	3-oxoacyl-ACP synthase	1.095	0.000	0.000
AS94_04170	hypothetical protein	0.867	0.000	0.000
AS94_04190	signal peptidase IB	1.090	0.000	0.000
AS94_04195	signal peptidase I	1.056	0.000	0.000
AS94_04200	hypothetical protein	0.632	0.000	0.000
AS94_04220	argininosuccinate synthase	6.222	0.000	0.000
AS94_04225	argininosuccinate lyase	5.619	0.000	0.000
AS94_04240	ornithine-oxoacid aminotransferase	0.351	0.001	0.003
AS94_04320	NADH dehydrogenase	0.899	0.000	0.000
AS94_04350	D-alanyl-lipoteichoic acid biosynthesis protein DltD	0.797	0.000	0.000
AS94_04360	D-alanyl transfer protein DltB	0.780	0.000	0.000
AS94_04365	D-alanine--poly(phosphoribitol) ligase	0.872	0.000	0.000
AS94_04395	hypothetical protein	0.454	0.012	0.026
AS94_04405	5'-nucleotidase	0.552	0.000	0.000
AS94_04410	membrane protein	0.580	0.002	0.005
AS94_04415	hypothetical protein	0.536	0.001	0.002
AS94_04445	cysteine desulfurase	0.264	0.017	0.035
AS94_04450	Fe-S cluster assembly protein SufD	0.594	0.000	0.000
AS94_04455	iron ABC transporter ATP-binding protein	0.651	0.000	0.000
AS94_04460	hypothetical protein	0.945	0.000	0.000
AS94_04485	thioredoxin	0.846	0.000	0.000
AS94_04490	topiosmerase	0.640	0.002	0.005
AS94_04525	peroxiredoxin	0.828	0.000	0.000
AS94_04540	phosphoglycerate mutase	1.008	0.000	0.000
AS94_04550	hypothetical protein	1.038	0.000	0.000
AS94_04575	hypothetical protein	0.714	0.002	0.005
AS94_04605	clumping factor A	0.877	0.000	0.000
AS94_04635	enterotoxin	0.311	0.004	0.010

AS94_04655	hypothetical protein	0.459	0.000	0.001
AS94_04670	hydrolase	1.617	0.000	0.000
AS94_04725	haloacid dehalogenase	1.509	0.000	0.000
AS94_04730	glycine/betaine MFS transporter	1.488	0.000	0.000
AS94_04735	long-chain fatty acid--CoA ligase	4.180	0.000	0.000
AS94_04740	acetyl-CoA acetyltransferase	2.242	0.000	0.000
AS94_04745	vraC	1.973	0.001	0.002
AS94_04750	hypothetical protein	3.222	0.000	0.000
AS94_04755	vraX	3.049	0.000	0.000
AS94_04780	amino acid permease	1.289	0.000	0.000
AS94_04810	mevalonate kinase	1.594	0.000	0.000
AS94_04815	diphosphomevalonate decarboxylase	1.814	0.000	0.000
AS94_04820	phosphomevalonate kinase	1.673	0.000	0.000
AS94_04840	transposase	0.710	0.000	0.000
AS94_04850	hypothetical protein	0.755	0.022	0.045
AS94_04865	oxidoreductase ion channel protein IolS	0.386	0.008	0.018
AS94_04950	lysyl-tRNA synthetase	0.452	0.000	0.000
AS94_04955	2-amino-4-hydroxy-6-hydroxymethyldihydropteridine pyrophosphokinase	1.104	0.000	0.000
AS94_04960	dihydroneopterin aldolase	1.683	0.000	0.000
AS94_04965	dihydropteroate synthase	2.204	0.000	0.000
AS94_04980	zinc metalloprotease	0.610	0.000	0.000
AS94_04985	hypoxanthine phosphoribosyltransferase	0.524	0.000	0.001
AS94_05140	hypothetical protein	0.718	0.002	0.005
AS94_05160	Na/Pi cotransporter	0.860	0.000	0.000
AS94_05330	membrane protein	5.305	0.002	0.005
AS94_05340	replication initiation factor family protein	2.706	0.013	0.028
AS94_05345	hypothetical protein	2.940	0.016	0.033
AS94_05350	hypothetical protein	3.020	0.014	0.031
AS94_05355	hypothetical protein	3.042	0.016	0.034

AS94_05435		integrase	1.176	0.009	0.021
AS94_05465		hemolysin III	0.562	0.000	0.000
AS94_05470		uridylyltransferase	0.526	0.000	0.000
AS94_05475		membrane protein	0.402	0.006	0.015
AS94_05480		hypothetical protein	0.399	0.001	0.002
AS94_05485		membrane protein	2.663	0.000	0.000
AS94_05525	*asp23*	alkaline shock protein 23	1.326	0.000	0.000
AS94_05530		membrane protein	0.988	0.000	0.000
AS94_05535		hypothetical protein	0.950	0.000	0.000
AS94_05540		glycine/betaine ABC transporter permease	1.635	0.000	0.000
AS94_05625		hyaluronate lyase	0.555	0.002	0.004
AS94_05650		toxin	1.123	0.000	0.000
AS94_05670		membrane protein	1.135	0.012	0.025
AS94_05680		30S ribosomal protein S9	1.190	0.000	0.000
AS94_05685		50S ribosomal protein L13	1.212	0.000	0.000
AS94_05785		50S ribosomal protein L5	0.333	0.013	0.027
AS94_05790		50S ribosomal protein L24	0.436	0.002	0.005
AS94_05800		30S ribosomal protein S17	0.567	0.003	0.007
AS94_05810		50S ribosomal protein L16	0.513	0.000	0.001
AS94_05815		30S ribosomal protein S3	0.501	0.000	0.001
AS94_05820		50S ribosomal protein L22	0.396	0.005	0.013
AS94_05825		30S ribosomal protein S19	0.439	0.004	0.009
AS94_05830		50S ribosomal protein L2	0.464	0.000	0.001
AS94_05840		50S ribosomal protein L4	0.427	0.001	0.003
AS94_05845		50S ribosomal protein L3	0.615	0.000	0.000
AS94_05850		30S ribosomal protein S10	0.939	0.000	0.000
AS94_05860		guanine permease	0.678	0.000	0.000
AS94_05900		aminoacyltransferase	0.292	0.011	0.024
AS94_06080		hypothetical protein	1.519	0.000	0.000
AS94_06085		sodium:proton antiporter	0.882	0.000	0.000
AS94_06090		octopine dehydrogenase	0.705	0.000	0.000
AS94_06145		LytR family transcriptional regulator	0.714	0.000	0.000

AS94_06170	RpiR family transcriptional regulator	0.363	0.006	0.014
AS94_06205	hypothetical protein	2.047	0.000	0.000
AS94_06245	imidazolonepropionase	2.049	0.000	0.000
AS94_06250	urocanate hydratase	1.987	0.000	0.000
AS94_06300	hypothetical protein	0.877	0.000	0.000
AS94_06310	sodium:glutamate symporter	1.092	0.000	0.000
AS94_06325	3-hydroxyacyl-CoA dehydrogenase	0.555	0.014	0.030
AS94_06365	membrane protein	1.854	0.000	0.000
AS94_06375	membrane protein	0.520	0.003	0.008
AS94_06425	malate:quinone oxidoreductase	0.620	0.000	0.000
AS94_06450	quinone oxidoreductase	0.523	0.000	0.001
AS94_06655	amino acid ABC transporter substrate-binding protein	0.566	0.000	0.000
AS94_06670	phosphoglyceromutase	0.512	0.000	0.000
AS94_06680	hypothetical protein	1.422	0.000	0.000
AS94_06685	gamma-hemolysin subunit A	0.618	0.005	0.011
AS94_06755	glycerate kinase	1.650	0.000	0.000
AS94_06760	membrane protein	1.748	0.000	0.000
AS94_06770	transcriptional regulator	1.261	0.000	0.000
AS94_06855	chloramphenicol resistance protein DHA1	0.710	0.000	0.001
AS94_06940	short-chain dehydrogenase	0.676	0.000	0.000
AS94_06945	aminobenzoyl-glutamate transporter	1.029	0.000	0.000
AS94_06980	hypothetical protein	0.494	0.004	0.010
AS94_07095	membrane protein	0.710	0.000	0.000
AS94_07100	glucarate transporter	0.367	0.019	0.040
AS94_07160	D-lactate dehydrogenase	0.537	0.001	0.003
AS94_07190	serine dehydratase subunit alpha	1.350	0.000	0.000
AS94_07195	serine dehydratase	1.304	0.000	0.000
AS94_07200	transcriptional regulator	1.123	0.000	0.000
AS94_07270	hydroxymethylglutaryl-CoA synthase	1.456	0.000	0.000
AS94_07280	Clp protease ATP-binding protein	1.513	0.000	0.000

AS94_07310		1-pyrroline-5-carboxylate dehydrogenase	0.884	0.000	0.000
AS94_07320		hypothetical protein	3.453	0.000	0.000
AS94_07345		dehydrosqualene desaturase	0.341	0.004	0.010
AS94_07350		dehydrosqualene synthase	1.071	0.000	0.000
AS94_07355		4_4'-diaponeurosporenoate glycosyltransferase	0.774	0.000	0.000
AS94_07360		diapolycopene oxygenase	0.709	0.000	0.000
AS94_07385		transglycosylase	0.996	0.000	0.000
AS94_07480		dihydroorotate dehydrogenase	0.910	0.000	0.000
AS94_07505		acyl esterase	0.367	0.015	0.032
AS94_07540		amino acid permease	1.179	0.000	0.000
AS94_07545		4-aminobutyrate aminotransferase	1.004	0.003	0.007
AS94_07550		membrane protein	1.650	0.002	0.006
AS94_07745		Replication and maintenance protein	14.571	0.000	0.000
AS94_07750		SAM-dependent methlyltransferase	15.751	0.000	0.000
AS94_07825		membrane protein	0.632	0.019	0.040
AS94_07835	*sarA*	transcriptional regulator	0.785	0.000	0.000
AS94_07855		recombinase	0.844	0.000	0.001
AS94_07860		cation:proton antiporter	1.176	0.000	0.000
AS94_07865		cation:proton antiporter	1.088	0.000	0.001
AS94_07870		cation:proton antiporter	1.431	0.000	0.000
AS94_07875		cation:proton antiporter	1.387	0.000	0.000
AS94_07880		cation:proton antiporter	1.154	0.002	0.006
AS94_07890		cation:proton antiporter	1.142	0.000	0.000
AS94_07900		manganese ABC transporter substrate-binding protein	0.683	0.000	0.000
AS94_07905		membrane protein	0.545	0.000	0.000
AS94_07910		phosphonate ABC transporter ATP-binding protein	0.862	0.000	0.000
AS94_07920		membrane protein	1.800	0.000	0.000
AS94_07955		D-alanyl-D-alanine carboxypeptidase	0.563	0.000	0.000
AS94_08060		inorganic phosphate transporter	0.434	0.001	0.002

AS94_08140	lysine decarboxylase	0.514	0.001	0.003
AS94_08150	hypothetical protein	0.818	0.000	0.000
AS94_08155	UDP pyrophosphate phosphatase	1.261	0.000	0.000
AS94_08160	cysteine ABC transporter ATP-binding protein	0.405	0.001	0.001
AS94_08165	cysteine ABC transporter ATP-binding protein	0.549	0.000	0.000
AS94_08190	membrane protein	0.539	0.000	0.000
AS94_08210	hypothetical protein	0.510	0.004	0.009
AS94_08215	multidrug MFS transporter	0.685	0.000	0.000
AS94_08250	hypothetical protein	0.340	0.002	0.006
AS94_08255	glyoxal reductase	0.753	0.000	0.000
AS94_08260	bactoprenol glucosyl transferase	0.614	0.000	0.000
AS94_08330	glycerol phosphate lipoteichoic acid synthase	1.264	0.000	0.000
AS94_08345	sulfonate ABC transporter ATP-binding protein	0.404	0.015	0.033
AS94_08350	ABC transporter permease	0.530	0.000	0.000
AS94_08375	peptide ABC transporter permease	0.279	0.020	0.042
AS94_08470	membrane protein	0.738	0.000	0.000
AS94_08480	hypothetical protein	1.192	0.000	0.000
AS94_08505	preprotein translocase subunit SecA	0.384	0.000	0.001
AS94_08575	hypothetical protein	0.803	0.000	0.000
AS94_08580	sporulation protein	0.500	0.000	0.001
AS94_08590	ATP-dependent Clp protease proteolytic subunit	0.457	0.000	0.000
AS94_08610	hypothetical protein	0.466	0.000	0.000
AS94_08615	transcriptional regulator	0.486	0.000	0.000
AS94_08660	ribonuclease R	0.397	0.000	0.001
AS94_08675	integrase	0.448	0.003	0.007
AS94_08685	hypothetical protein	3.481	0.000	0.000
AS94_08690	DNA-binding protein	6.649	0.000	0.000
AS94_08705	pathogenicity island protein	2.733	0.008	0.018

AS94_08810	NA	1.493	0.000	0.000
AS94_08925	DEAD/DEAH box helicase	0.507	0.000	0.000
AS94_08960	phospholipase D	0.656	0.000	0.000
AS94_08965	phosphohydrolase	0.530	0.000	0.001
AS94_08970	membrane protein	0.613	0.000	0.000
AS94_09010	3-hydroxyacyl-ACP dehydratase	0.341	0.024	0.048
AS94_09015	UDP-N-acetylglucosamine 1-carboxyvinyltransferase	0.562	0.000	0.000
AS94_09020	membrane protein	1.061	0.013	0.028
AS94_09040	F0F1 ATP synthase subunit alpha	0.694	0.000	0.000
AS94_09045	F0F1 ATP synthase subunit delta	0.533	0.000	0.000
AS94_09050	F0F1 ATP synthase subunit B	0.776	0.000	0.000
AS94_09055	F0F1 ATP synthase subunit C	0.318	0.021	0.042
AS94_09060	F0F1 ATP synthase subunit A	0.435	0.000	0.000
AS94_09065	ATP synthase	0.419	0.006	0.013
AS94_09070	UDP-N-acetylglucosamine 2-epimerase	0.710	0.000	0.000
AS94_09075	uracil phosphoribosyltransferase	0.716	0.000	0.000
AS94_09080	serine hydroxymethyltransferase	0.589	0.000	0.000
AS94_09085	hypothetical protein	0.661	0.000	0.000
AS94_09100	N5-glutamine S-adenosyl-L-methionine-dependent methyltransferase	0.552	0.000	0.000
AS94_09105	peptide chain release factor 1	0.684	0.000	0.000
AS94_09110	thymidine kinase	0.545	0.000	0.001
AS94_09115	50S ribosomal protein L31	0.378	0.002	0.006
AS94_09120	transcription termination factor Rho	0.286	0.010	0.022
AS94_09150	CTP synthetase	1.261	0.000	0.000
AS94_09160	acetyltransferase	1.323	0.000	0.000
AS94_09165	pantothenate kinase	0.898	0.000	0.000
AS94_09230	mannose-6-phosphate isomerase	0.526	0.001	0.002
AS94_09240	ArsR family transcriptional regulator	1.854	0.000	0.000
AS94_09245	cation transporter	1.422	0.000	0.000
AS94_09250	lytic regulatory protein	0.641	0.000	0.000

AS94_09275	PTS mannitol transporter subunit IIB	1.502	0.000	0.001
AS94_09280	PTS lactose transporter subunit IIB	1.979	0.000	0.000
AS94_09285	PTS mannitol transporter subunit IIA	1.613	0.000	0.000
AS94_09290	mannitol-1-phosphate 5-dehydrogenase	1.814	0.000	0.000
AS94_09315	arginase	0.803	0.000	0.000
AS94_09380	iron ABC transporter permease	0.490	0.000	0.001
AS94_09450	membrane protein	0.766	0.008	0.018
AS94_09490	surface protein	0.567	0.000	0.000
AS94_09560	capsular polysaccharide biosynthesis protein CapA	1.554	0.000	0.000
AS94_09565	capsular polysaccharide biosynthesis protein Cap5B	1.706	0.000	0.000
AS94_09570	capsular polysaccharide biosynthesis protein Cap8C	1.635	0.000	0.000
AS94_09575	polysaccharide biosynthesis protein EpsC	1.580	0.000	0.000
AS94_09580	UDP-glucose 4-epimerase	1.728	0.000	0.000
AS94_09585	capsular polysaccharide biosynthesis protein Cap8F	1.526	0.000	0.000
AS94_09590	UDP-N-acetylglucosamine 2-epimerase	1.646	0.000	0.000
AS94_09595	capsular polysaccharide biosynthesis protein	1.591	0.000	0.000
AS94_09600	capsular polysaccharide biosynthesis protein	1.343	0.000	0.000
AS94_09605	capsular polysaccharide biosynthesis protein	1.211	0.000	0.000
AS94_09610	capsular polysaccharide biosynthesis protein	0.896	0.001	0.001
AS94_09615	glycosyltransferase family 1	0.556	0.000	0.001
AS94_09630	UDP-N-acetyl-D-mannosamine dehydrogenase	0.311	0.025	0.049
AS94_09690	multidrug MFS transporter	0.882	0.000	0.000

AS94_09755	cellobiose operon outer surface protein	0.638	0.000	0.000
AS94_09760	N-acetylmuramic acid-6-phosphate etherase	0.578	0.000	0.000
AS94_09765	permease	0.676	0.000	0.000
AS94_09770	RpiR family transcriptional regulator	0.881	0.000	0.000
AS94_09820	peptidase M23	1.093	0.000	0.000
AS94_09865	antiporter	2.494	0.000	0.000
AS94_09920	3-hydroxyacyl-CoA dehydrogenase	0.879	0.001	0.001
AS94_09925	glutaryl-CoA dehydrogenase	1.155	0.003	0.006
AS94_09930	long-chain fatty acid--CoA ligase	1.533	0.000	0.000
AS94_09935	coenzyme A transferase	1.762	0.000	0.000
AS94_09955	NmrA family protein	2.177	0.000	0.000
AS94_09960	DeoR family transcriptional regulator	1.806	0.000	0.000
AS94_09985	lactate dehydrogenase	0.788	0.000	0.000
AS94_09995	inosine-uridine preferring nucleoside hydrolase	0.623	0.002	0.004
AS94_10035	2-C-methyl-D-erythritol 4-phosphate cytidylyltransferase	0.982	0.000	0.000
AS94_10040	ribitol-5-phosphate dehydrogenase	0.721	0.001	0.002
AS94_10045	teichoic acid biosynthesis protein	0.748	0.000	0.000
AS94_10050	CDP-glycerol:glycerophosphate glycerophosphotransferase	0.775	0.000	0.000
AS94_10125	ribokinase	2.808	0.000	0.000
AS94_10130	ribose pyranase	2.726	0.000	0.000
AS94_10135	ribose transporter RbsU	2.853	0.000	0.000
AS94_10200	virulence factor EsxA	0.390	0.001	0.002
AS94_10205	type VII secretion protein EsaA	0.914	0.000	0.000
AS94_10220	type VII secretion protein EssB	0.596	0.024	0.049
AS94_10315	hypothetical protein	0.745	0.000	0.001
AS94_10325	branched-chain amino acid transporter II carrierprotein	0.968	0.000	0.000
AS94_10390	histidine transporter	2.081	0.000	0.000
AS94_10395	lipase	0.498	0.000	0.000

AS94_10470	sn-glycerol-3-phosphate transporter	1.821	0.000	0.000
AS94_10555	acetyl-CoA acetyltransferase	0.837	0.000	0.000
AS94_10565	5-methyltetrahydropteroyltriglutamate--homocysteine methyltransferase	0.852	0.000	0.000
AS94_10570	5_10-methylenetetrahydrofolate reductase	0.983	0.000	0.000
AS94_10575	cystathionine beta-lyase	0.840	0.013	0.028
AS94_10580	cystathionine gamma-synthase	0.782	0.015	0.032
AS94_10600	GTP-binding protein YchF	0.305	0.024	0.049
AS94_10660	peptidase	1.829	0.000	0.000
AS94_10670	membrane protein	0.941	0.000	0.000
AS94_10725	hypothetical protein	0.541	0.000	0.000
AS94_10785	growth inhibitor PemK	0.719	0.000	0.000
AS94_10795	hypothetical protein	0.861	0.002	0.006
AS94_10800	hypothetical protein	0.786	0.000	0.000
AS94_10935	cobalamin synthesis protein CobW	2.380	0.000	0.000
AS94_10985	methionine ABC transporter ATP-binding protein	1.535	0.000	0.000
AS94_11070	acetyltransferase	0.620	0.009	0.019
AS94_11170	16S rRNA methyltransferase	0.540	0.000	0.001
AS94_11175	tRNA uridine 5-carboxymethylaminomethyl modification protein	0.569	0.000	0.000
AS94_11180	tRNA modification GTPase	0.481	0.000	0.001
AS94_11185	ribonuclease P	0.873	0.000	0.001
AS94_11190	50S ribosomal protein L34	0.924	0.000	0.000
AS94_11195	chromosome replication protein DnaA	0.548	0.000	0.000
AS94_11200	DNA polymerase III subunit beta	0.635	0.000	0.000
AS94_11210	recombinase F	0.481	0.000	0.000
AS94_11215	DNA gyrase subunit B	0.557	0.000	0.000
AS94_11220	DNA topoisomerase IV subunit A	0.725	0.000	0.000
AS94_11230	histidine ammonia-lyase	0.628	0.000	0.000

AS94_11235	seryl-tRNA synthetase	1.022	0.000	0.000
AS94_11275	adenylosuccinate synthetase	2.591	0.000	0.000
AS94_11320	50S rRNA methyltransferase	0.509	0.001	0.002
AS94_11355	penicillin-binding protein	0.635	0.000	0.000
AS94_11360	methicillin resistance protein	0.415	0.006	0.014
AS94_11365	hypothetical protein	0.851	0.009	0.019
AS94_11430	hydrolase	2.561	0.000	0.000
AS94_11470	UDP-glucose 4-epimerase	0.795	0.000	0.000
AS94_11485	2-amino-3-ketobutyrate CoA ligase	0.544	0.000	0.000
AS94_11515	50S ribosomal protein L7	0.294	0.016	0.034
AS94_11530	16S rRNA methyltransferase	0.767	0.000	0.000
AS94_11535	50S ribosomal protein L7/L12	0.976	0.000	0.000
AS94_11540	50S ribosomal protein L10	1.082	0.000	0.000
AS94_11545	50S ribosomal protein L1	1.176	0.000	0.000
AS94_11550	50S ribosomal protein L11	0.711	0.000	0.000
AS94_11575	hypothetical protein	0.417	0.015	0.031
AS94_11610	DNA repair protein RadA	0.355	0.005	0.012
AS94_11615	ATP-dependent Clp protease ATP-binding protein	0.396	0.000	0.000
AS94_11665	DNA damage-inducible protein DinB	0.614	0.000	0.001
AS94_11680	lactonase	0.993	0.000	0.000
AS94_11740	lipase	1.297	0.000	0.000
AS94_11765	transcriptional regulator	0.602	0.000	0.000
AS94_11815	hypothetical protein	0.695	0.004	0.009
AS94_11825	flavin reductase	0.795	0.001	0.002
AS94_11855	preprotein translocase subunit SecA	0.320	0.012	0.027
AS94_11870	surface anchored protein	0.933	0.000	0.000
AS94_11875	isochorismatase hydrolase	0.341	0.005	0.012
AS94_11880	N-acetylmuramoyl-L-alanine amidase	0.521	0.000	0.000
AS94_11940	carbamate kinase	0.510	0.000	0.001
AS94_11945	Crp/Fnr family transcriptional regulator	0.580	0.000	0.000

AS94_11985	multidrug ABC transporter ATP-binding protein	0.925	0.000	0.000
AS94_11990	hypothetical protein	0.695	0.000	0.000
AS94_12020	RecX family transcriptional regulator	0.925	0.000	0.000
AS94_12025	glycosyltransferase	2.944	0.000	0.000
AS94_12030	general stress protein	0.703	0.000	0.000
AS94_12035	hypothetical protein	0.749	0.015	0.032
AS94_12400	protein tyrosine phosphatase	0.394	0.001	0.004
AS94_12410	ribonuclease BN	1.228	0.000	0.000
AS94_12415	LuxR family transcriptional regulator	3.187	0.000	0.000
AS94_12420	sensor histidine kinase	3.380	0.000	0.000
AS94_12425	transporter	3.431	0.000	0.000
AS94_12430	hypothetical protein	3.841	0.000	0.000
AS94_12460	glutamine amidotransferase	0.539	0.000	0.000
AS94_12465	UDP-N-acetylmuramate--alanine ligase	0.705	0.000	0.000
AS94_12475	DNA polymerase III subunit epsilon	0.380	0.009	0.021
AS94_12490	RNA methyltransferase	0.594	0.000	0.000
AS94_12540	hypothetical protein	0.527	0.002	0.005
AS94_12545	adenylosuccinate lyase	0.810	0.000	0.000
AS94_12550	cysteine protease	1.797	0.000	0.000
AS94_12555	staphostatin A	1.108	0.001	0.004
AS94_12590	C4-dicarboxylate ABC transporter	0.379	0.006	0.014
AS94_12630	beta-lactamase	0.440	0.017	0.036
AS94_12700	membrane protein	1.446	0.000	0.000
AS94_12705	antibiotic ABC transporter ATP-binding protein	1.061	0.000	0.000
AS94_12710	membrane protein	1.386	0.000	0.001
AS94_12715	sodium ABC transporter ATP-binding protein	1.491	0.000	0.000
AS94_12720	GntR family transcriptional regulator	1.459	0.000	0.000
AS94_12755	complement inhibitor	0.994	0.000	0.000
AS94_12765	peptidoglycan hydrolase	0.700	0.000	0.000
AS94_12850	molecular chaperone GroES	0.653	0.000	0.000

AS94_12870		hydrolase	0.334	0.016	0.035
AS94_12935		ABC transporter ATP-binding protein	0.476	0.002	0.006
AS94_12970		dihydroxy-acid dehydratase	1.657	0.000	0.000
AS94_12975		acetolactate synthase	1.006	0.000	0.000
AS94_13490		peptidyl-prolyl cis-trans isomerase	2.346	0.000	0.000
AS94_13500		DNA double-strand break repair Rad50 ATPase	0.307	0.003	0.007
AS94_13515		membrane protein	0.579	0.002	0.005
AS94_13520		Cro/Cl family transcriptional regulator	0.658	0.000	0.000
AS94_13575		amino acid ABC transporter ATP-binding protein	4.177	0.000	0.000
AS94_13580		glutamate ABC transporter permease	4.391	0.000	0.000
AS94_12040	ϕSA169	hypothetical protein	8.459	0.000	0.000
AS94_12045		XRE family transcriptional regulator	12.889	0.000	0.000
AS94_12050		hypothetical protein	11.224	0.000	0.000
AS94_12055		autolysin	10.844	0.000	0.000
AS94_12060		holin	8.647	0.000	0.000
AS94_12065		hypothetical protein	9.207	0.000	0.000
AS94_12070		tail protein	11.976	0.000	0.000
AS94_12075		cell wall hydrolase	12.788	0.000	0.000
AS94_12080		hypothetical protein	9.512	0.000	0.000
AS94_12090		hypothetical protein	8.652	0.000	0.000
AS94_12095		hypothetical protein	12.193	0.000	0.000
AS94_12100		minor structural protein	12.567	0.000	0.000
AS94_12105		peptidase	12.720	0.000	0.000
AS94_12110		phage tail protein	11.456	0.000	0.000
AS94_12115		membrane protein	13.916	0.000	0.000
AS94_12120		phi 11	9.902	0.000	0.000
AS94_12125		hypothetical protein	10.462	0.000	0.000
AS94_12130		tail protein	12.239	0.000	0.000
AS94_12135		phi 11	9.806	0.000	0.000
AS94_12140		hypothetical protein	9.238	0.000	0.000
AS94_12145		hypothetical protein	8.869	0.000	0.000
AS94_12150		phage head-tail adapter protein	10.259	0.000	0.000

AS94_12155		phi 11	8.348	0.000	0.000
AS94_12160		hypothetical protein	12.909	0.000	0.000
AS94_12165		phage capsid protein	13.409	0.000	0.000
AS94_12170		hypothetical protein	7.512	0.000	0.000
AS94_12175		phage head morphogenesis protein	12.222	0.000	0.000
AS94_12180		phage portal protein	12.555	0.000	0.000
AS94_12185		hypothetical protein	12.014	0.000	0.000
AS94_12190		terminase	11.070	0.000	0.000
AS94_12195		transcriptional regulator	9.179	0.000	0.000
AS94_12210		hypothetical protein	7.003	0.000	0.000
AS94_12215		hypothetical protein	5.651	0.001	0.002
AS94_12220		dUTP pyrophosphatase	9.617	0.000	0.000
AS94_12230		hypothetical protein	6.411	0.000	0.000
AS94_12240		hypothetical protein	8.519	0.000	0.000
AS94_12250		hypothetical protein	5.258	0.022	0.045
AS94_12270		DNA N-6-adenine-methyltransferase	6.555	0.000	0.000
AS94_12295		hypothetical protein	9.802	0.000	0.000
AS94_12320		hypothetical protein	5.636	0.002	0.005
AS94_12325		hypothetical protein	10.255	0.000	0.000
AS94_12330		hypothetical protein	6.051	0.000	0.001
AS94_12340		hypothetical protein	7.580	0.000	0.000
AS94_12345		BRO-like protein	12.337	0.000	0.000
AS94_12350		hypothetical protein	10.705	0.000	0.000
AS94_12355		XRE family transcriptional regulator	8.504	0.000	0.000
AS94_12360		transcriptional regulator	12.464	0.000	0.000
AS94_12365		hypothetical protein	8.895	0.000	0.000
AS94_12370		repressor	13.022	0.000	0.000
AS94_12375		integrase	10.916	0.000	0.000
AS94_13265	mutual prophage	hypothetical protein	4.162	0.000	0.000
AS94_13290		hypothetical protein	8.740	0.000	0.000
AS94_13295		hypothetical protein	5.378	0.011	0.025
AS94_13305		DNA replication protein DnaC	0.938	0.000	0.001
AS94_13310		replication protein	10.627	0.000	0.000
AS94_13315		hypothetical protein	10.070	0.000	0.000

AS94_13395	integrase	0.983	0.000	0.000
