# Supplementary material for: Transcriptome Analyses of Prophage in Mediating Persistent Methicillin-Resistant Staphylococcus aureus Endovascular Infection
Source: Genes (Basel). 2022 Aug 25;13(9):1527. doi: 10.3390/genes13091527 (PMC9498598; doi:10.3390/genes13091527)
Supplement: Supplementary file 1 [file genes-13-01527-s001.zip › Table S4.pdf]

Table S4. Down-regulated DEGs in 300-169 vs. 301-188

locus	gene	group	product	log_2(fold change)	*p* value	*p* adj
AS94_00045			membrane protein	-0.688	0.001	0.002
AS94_00055			dipeptidyl aminopeptidase	-0.628	0.000	0.000
AS94_00195			multidrug MFS transporter	-0.319	0.021	0.044
AS94_00205			hypothetical protein	-0.923	0.000	0.000
AS94_00210			peptidase	-0.416	0.005	0.012
AS94_00245			phosphotransferase	-0.858	0.000	0.000
AS94_00250			tRNA (guanine-N(7)-)-methyltransferase	-0.494	0.000	0.000
AS94_00255			hypothetical protein	-0.984	0.000	0.000
AS94_00265			glutamyl aminopeptidase	-0.359	0.006	0.014
AS94_00270			thioredoxin	-0.376	0.007	0.017
AS94_00305			3-deoxy-7-phosphoheptulonate synthase	-1.464	0.000	0.000
AS94_00370			3-phosphoglycerate dehydrogenase	-0.473	0.001	0.002
AS94_00395			hypothetical protein	-0.414	0.017	0.035
AS94_00435			universal stress protein UspA	-2.146	0.000	0.000
AS94_00445			dipeptidase	-0.631	0.000	0.000
AS94_00450		host genes	beta-lactamase	-1.246	0.000	0.000
AS94_00460			universal stress protein UspA	-1.098	0.000	0.000
AS94_00505			6-phosphofructokinase	-0.997	0.000	0.000
AS94_00510			pyruvate kinase	-0.667	0.000	0.000
AS94_00525			isocitrate dehydrogenase	-0.612	0.000	0.000
AS94_00545			DNA polymerase I	-0.519	0.000	0.000
AS94_00550			formamidopyrimidine-DNA glycosylase	-0.486	0.000	0.000
AS94_00555			dephospho-CoA kinase	-1.148	0.000	0.000
AS94_00565			NrdR family transcriptional regulator	-0.339	0.021	0.042
AS94_00570			chromosome replication protein DnaB	-0.268	0.019	0.040
AS94_00575			primosomal protein DnaI	-0.406	0.000	0.001
AS94_00580			threonyl-tRNA synthase	-1.711	0.000	0.000
AS94_00585			gamma-aminobutyrate permease	-1.052	0.000	0.000
AS94_00645			uroporphyrinogen III synthase	-0.444	0.005	0.011
AS94_00650			delta-aminolevulinic acid dehydratase	-0.273	0.022	0.044

AS94_00655	glutamate-1-semialdehyde aminotransferase	-0.376	0.001	0.002
AS94_00665	DNA-3-methyladenine glycosylase	-1.452	0.000	0.000
AS94_00730	50S ribosomal protein L27	-1.294	0.000	0.000
AS94_00735	GTPase CgtA	-0.331	0.006	0.014
AS94_00785	GTP pyrophosphokinase	-0.367	0.000	0.001
AS94_00795	cell wall amidase	-0.459	0.000	0.001
AS94_00800	hypothetical protein	-0.369	0.014	0.029
AS94_00810	aspartyl-tRNA synthase	-0.666	0.000	0.000
AS94_00840	hypothetical protein	-0.698	0.000	0.000
AS94_00855	hypothetical protein	-0.479	0.014	0.031
AS94_00860	hypothetical protein	-0.429	0.000	0.001
AS94_00870	hypothetical protein	-0.524	0.001	0.002
AS94_00875	Holliday junction resolvase	-0.517	0.000	0.000
AS94_00880	hypothetical protein	-0.394	0.003	0.006
AS94_00905	transcription elongation factor GreA	-0.418	0.001	0.003
AS94_01005	deoxycytidylate deaminase	-0.636	0.000	0.001
AS94_01185	superoxide dismutase	-0.792	0.000	0.000
AS94_01260	shikimate kinase	-0.601	0.000	0.001
AS94_01265	glycine cleavage system protein T	-0.624	0.000	0.000
AS94_01270	glycine dehydrogenase subunit 1	-0.769	0.000	0.000
AS94_01275	glycine dehydrogenase subunit 2	-1.048	0.000	0.000
AS94_01280	rhodanese	-0.291	0.018	0.038
AS94_01370	2-oxoglutarate dehydrogenase E2	-0.549	0.000	0.000
AS94_01405	transcriptional regulator	-1.046	0.000	0.000
AS94_01410	trehalose-6-phosphate hydrolase	-1.817	0.000	0.000
AS94_01430	pyrroline-5-carboxylate reductase	-0.782	0.000	0.000
AS94_01455	Fur family transcriptional regulator	-0.319	0.013	0.028
AS94_01460	tyrosine recombinase XerD	-0.925	0.000	0.000
AS94_01490	sensor histidine kinase	-0.390	0.000	0.001
AS94_01560	glycerol-3-phosphate dehydrogenase	-0.516	0.000	0.000
AS94_01565	DNA-binding protein	-0.800	0.000	0.000

AS94_01605	3-phosphoshikimate 1-carboxyvinyltransferase	-0.312	0.022	0.046
AS94_01630	hypothetical protein	-0.556	0.006	0.013
AS94_01665	endonuclease III	-0.449	0.013	0.028
AS94_01700	hypothetical protein	-0.394	0.000	0.001
AS94_01730	alanine dehydrogenase	-0.505	0.001	0.003
AS94_01735	threonine dehydratase	-0.607	0.000	0.000
AS94_01740	amino acid permease	-0.388	0.009	0.020
AS94_01745	quinolone resistance protein NorB	-0.667	0.000	0.000
AS94_01750	matrix-binding protein	-0.438	0.000	0.000
AS94_01775	virulence factor	-0.295	0.009	0.021
AS94_01780	hypothetical protein	-0.493	0.000	0.001
AS94_01795	hypothetical protein	-0.462	0.000	0.000
AS94_01800	methionine sulfoxide reductase A	-0.421	0.000	0.000
AS94_01805	methionine sulfoxide reductase B	-0.355	0.005	0.012
AS94_01810	PTS glucose transporter subunit IIA	-0.295	0.012	0.025
AS94_01920	hypothetical protein	-1.003	0.000	0.000
AS94_01925	cold-shock protein	-0.320	0.017	0.036
AS94_01935	protease	-0.737	0.000	0.000
AS94_02100	aminopeptidase	-0.884	0.000	0.000
AS94_02105	prephenate dehydrogenase	-0.747	0.000	0.000
AS94_02110	DNA repair protein MucB	-1.701	0.000	0.000
AS94_02115	4-oxalocrotonate tautomerase	-1.777	0.000	0.000
AS94_02125	methionine sulfoxide reductase A	-0.532	0.000	0.001
AS94_02145	transcriptional regulator	-0.589	0.005	0.012
AS94_02165	glycerol-3-phosphate acyltransferase	-0.456	0.000	0.001
AS94_02230	XRE family transcriptional regulator	-1.344	0.000	0.000
AS94_02255	50S ribosomal protein L33	-0.526	0.002	0.004
AS94_02260	catalase	-0.479	0.000	0.000
AS94_02270	hypothetical protein	-0.544	0.000	0.001
AS94_02520	glutathione peroxidase	-0.375	0.012	0.026
AS94_02530	tRNA delta(2)-isopentenylpyrophosphate transferase	-0.440	0.013	0.028

AS94_02535	lysophospholipase	-0.980	0.000	0.000
AS94_02540	glycerol-3-phosphate dehydrogenase	-0.736	0.000	0.000
AS94_02590	2-oxoacid ferredoxin oxidoreductase subunit beta	-0.782	0.000	0.000
AS94_02595	2-oxoglutarate ferredoxin oxidoreductase subunitalpha	-0.948	0.000	0.000
AS94_02615	protein RecA	-0.281	0.006	0.013
AS94_02660	cell division protein FtsK	-0.277	0.014	0.030
AS94_02665	ribonuclease J	-0.452	0.000	0.000
AS94_02690	ribosome-binding factor A	-0.467	0.001	0.002
AS94_02720	DNA polymerase III subunit alpha	-0.333	0.002	0.004
AS94_02865	signal recognition particle protein Srp54	-0.764	0.000	0.000
AS94_02930	50S ribosomal protein L28	-1.592	0.000	0.000
AS94_02990	primosome assembly protein PriA	-0.682	0.000	0.000
AS94_02995	phosphopantothenoylcysteine decarboxylase	-0.718	0.000	0.000
AS94_03000	DNA-directed RNA polymerase subunit omega	-0.981	0.000	0.001
AS94_03005	guanylate kinase	-0.391	0.002	0.005
AS94_03075	glyoxalase	-1.094	0.000	0.000
AS94_03080	isoleucyl-tRNA synthetase	-0.878	0.000	0.000
AS94_03170	5'-nucleotidase	-0.503	0.000	0.000
AS94_03205	hypothetical protein	-0.894	0.000	0.000
AS94_03210	carbamate kinase	-2.248	0.000	0.000
AS94_03215	ornithine carbamoyltransferase	-0.905	0.000	0.000
AS94_03290	phosphoesterase	-1.105	0.000	0.000
AS94_03295	deoxyribonucleotide triphosphate pyrophosphatase	-1.087	0.000	0.000
AS94_03300	glutamate racemase	-1.035	0.000	0.000
AS94_03325	thioredoxin	-0.716	0.000	0.000
AS94_03355	phenylalanyl-tRNA synthase subunit beta	-0.647	0.000	0.000
AS94_03380	heme ABC transporter permease	-1.126	0.009	0.021
AS94_03425	phosphopantetheine adenylyltransferase	-0.633	0.000	0.001

AS94_03485	hypothetical protein	-0.477	0.020	0.041
AS94_03505	inositol monophosphatase	-0.694	0.000	0.000
AS94_03510	hypothetical protein	-0.787	0.000	0.000
AS94_03515	manganese transporter	-0.467	0.000	0.000
AS94_03590	hypothetical protein	-0.498	0.001	0.002
AS94_03635	SAM-dependent methyltransferase	-1.007	0.000	0.000
AS94_03650	thiamine ABC transporter permease	-0.662	0.002	0.006
AS94_03655	ABC transporter ATP-binding protein	-0.672	0.000	0.000
AS94_03660	cobalt ABC transporter permease	-1.017	0.000	0.000
AS94_03735	quinol oxidase subunit 2	-0.806	0.000	0.000
AS94_03740	quinol oxidase subunit 1	-1.580	0.000	0.000
AS94_03745	cytochrome O ubiquinol oxidase	-1.927	0.000	0.000
AS94_03750	quinol oxidase subunit 4	-2.152	0.000	0.000
AS94_03760	hypothetical protein	-0.324	0.012	0.027
AS94_03775	mannosyl-glycoprotein endo-beta-N-acetylglucosamidase	-0.453	0.000	0.000
AS94_03780	MarR family transcriptional regulator	-0.911	0.000	0.001
AS94_03795	glutamyl endopeptidase	-2.628	0.000	0.000
AS94_03800	cysteine protease	-2.378	0.000	0.000
AS94_03805	cysteine protease	-1.422	0.008	0.017
AS94_03810	dihydroxynaphthoic acid synthetase	-1.448	0.000	0.000
AS94_03815	2-succinyl-6-hydroxy-2_ 4-cyclohexadiene-1-carboxylate synthase	-1.214	0.000	0.000
AS94_03820	2-succinyl-5-enolpyruvyl-6-hydroxy-3-cyclohexene-1-carboxylate synthase	-0.629	0.000	0.000
AS94_03835	acetyltransferase	-0.811	0.000	0.000
AS94_03870	bacteriocin ABC transporter ATP-binding protein	-0.675	0.017	0.035
AS94_03880	bacteriocin-associated integral membrane protein	-0.751	0.000	0.001
AS94_03900	IDEAL domain protein	-0.938	0.000	0.000
AS94_03940	peptide chain release factor 1	-0.321	0.003	0.008
AS94_03995	magnesium transporter MgtE	-0.344	0.014	0.029

AS94_04000	ribosomal large subunit pseudouridine synthase D	-0.439	0.007	0.017
AS94_04005	inorganic polyphosphate/ATP-NAD kinase	-0.574	0.000	0.000
AS94_04010	GTP pyrophosphokinase	-0.890	0.000	0.000
AS94_04020	adenylate cyclase	-0.414	0.017	0.036
AS94_04025	Hemoglobin-like protein HbO	-0.403	0.016	0.033
AS94_04045	competence negative regulator MecA	-0.638	0.000	0.000
AS94_04130	MAP domain protein	-0.945	0.000	0.000
AS94_04175	hypothetical protein	-0.811	0.000	0.000
AS94_04180	ATP-dependent DNA helicase subunit A	-0.772	0.000	0.000
AS94_04185	ATP-dependent DNA helicase subunit B	-0.815	0.000	0.000
AS94_04215	glucose-6-phosphate isomerase	-0.374	0.002	0.006
AS94_04230	glycerophosphoryl diester phosphodiesterase	-0.606	0.000	0.001
AS94_04245	NADH-dependent flavin oxidoreductase	-0.844	0.000	0.000
AS94_04285	cation:proton antiporter	-0.574	0.015	0.032
AS94_04290	cation:proton antiporter	-2.171	0.000	0.000
AS94_04295	cation:proton antiporter	-0.808	0.002	0.005
AS94_04300	hypothetical protein	-1.823	0.000	0.000
AS94_04305	thioesterase	-0.775	0.000	0.000
AS94_04310	sodium:proton antiporter	-0.610	0.001	0.001
AS94_04325	hypothetical protein	-0.429	0.004	0.010
AS94_04345	nitrogen-fixing protein NifU	-0.560	0.001	0.002
AS94_04375	2-ketogluconate reductase	-0.394	0.009	0.021
AS94_04380	HAD family hydrolase	-0.332	0.018	0.037
AS94_04505	arsenate reductase	-0.914	0.000	0.000
AS94_04510	thioredoxin	-0.347	0.008	0.018
AS94_04560	membrane protein	-2.763	0.000	0.000
AS94_04580	cold-shock protein	-0.742	0.000	0.000
AS94_04585	thermonuclease	-1.632	0.000	0.000
AS94_04630	enterotoxin I	-1.137	0.000	0.000
AS94_04645	hypothetical protein	-0.575	0.000	0.000
AS94_04660	transposase	-0.990	0.000	0.000

AS94_04665	transposase	-0.790	0.001	0.002
AS94_04675	hydrolase	-0.504	0.000	0.000
AS94_04700	deacetylase	-0.329	0.012	0.026
AS94_04710	glucosamine-6-phosphate deaminase	-0.712	0.000	0.000
AS94_04720	6-phospho 3-hexuloisomerase	-0.333	0.008	0.017
AS94_04760	pyridoxal kinase	-0.486	0.000	0.000
AS94_04890	acetaldehyde reductase	-0.374	0.016	0.034
AS94_04970	cysteine synthase	-0.320	0.005	0.011
AS94_04975	heat shock protein Hsp33	-0.609	0.000	0.000
AS94_04995	hypothetical protein	-0.584	0.000	0.000
AS94_05010	nucleotide pyrophosphohydrolase	-0.656	0.000	0.000
AS94_05035	ribose-phosphate pyrophosphokinase	-0.407	0.000	0.000
AS94_05040	N-acetylglucosamine-1-phosphate uridyltransferase	-0.255	0.019	0.040
AS94_05085	methionyl-tRNA synthetase	-0.427	0.000	0.000
AS94_05105	DNA replication protein YabA	-0.878	0.000	0.000
AS94_05110	signal peptidase II	-0.474	0.000	0.001
AS94_05120	hypothetical protein	-0.758	0.000	0.000
AS94_05125	thymidylate kinase	-0.806	0.000	0.000
AS94_05130	lysine decarboxylase	-0.873	0.000	0.000
AS94_05145	aspartate aminotransferase	-0.921	0.009	0.020
AS94_05155	hypothetical protein	-0.494	0.001	0.002
AS94_05280	hypothetical protein	-1.235	0.020	0.041
AS94_05290	integrase	-0.360	0.019	0.039
AS94_05410	transposase	-2.153	0.000	0.000
AS94_05425	hypothetical protein	-0.668	0.002	0.006
AS94_05440	NA	-0.518	0.015	0.032
AS94_05445	chromosome partitioning protein ParA	-0.481	0.000	0.000
AS94_05500	iron citrate ABC transporter substrate-binding protein	-1.581	0.000	0.000
AS94_05555	alcohol dehydrogenase	-0.797	0.000	0.000
AS94_05560	alcohol dehydrogenase	-0.755	0.000	0.000
AS94_05570	6-phospho-beta-galactosidase	-2.586	0.000	0.000

AS94_05575	PTS lactose transporter subunit IIBC	-2.334	0.000	0.000
AS94_05580	PTS lactose transporter subunit IIA	-3.043	0.000	0.000
AS94_05585	tagatose-bisphosphate aldolase	-2.569	0.000	0.000
AS94_05590	tagatose-6-phosphate kinase	-2.374	0.000	0.000
AS94_05595	galactose-6-phosphate isomerase	-2.300	0.000	0.000
AS94_05600	galactose-6-phosphate isomerase	-2.599	0.000	0.000
AS94_05605	DeoR family transcriptional regulator	-0.556	0.002	0.004
AS94_05610	NAD-dependent deacetylase	-0.456	0.022	0.044
AS94_05615	2_5-diketo-D-gluconic acid reductase	-0.809	0.000	0.000
AS94_05655	alpha-acetolactate decarboxylase	-2.037	0.000	0.000
AS94_05660	acetolactate synthase	-2.320	0.000	0.000
AS94_05690	tRNA pseudouridine synthase A	-0.335	0.015	0.032
AS94_05705	cobalt ABC transporter ATP-binding protein	-0.473	0.005	0.013
AS94_05735	translation initiation factor IF-1	-0.351	0.006	0.013
AS94_05870	GNAT family acetyltransferase	-0.873	0.000	0.000
AS94_05880	malonate transporter	-0.768	0.000	0.000
AS94_05890	membrane protein	-0.495	0.001	0.003
AS94_05920	MarR family transcriptional regulator	-0.444	0.013	0.028
AS94_05930	molybdenum cofactor biosynthesis protein A	-0.594	0.000	0.000
AS94_05935	molybdopterin-guanine dinucleotide biosynthesis protein A	-0.681	0.000	0.000
AS94_05945	molybdopterin synthase subunit 2	-1.051	0.000	0.000
AS94_05950	molybdopterin-guanine dinucleotide biosynthesis protein B	-0.667	0.001	0.003
AS94_05955	molybdopterin molybdenumtransferase	-1.838	0.000	0.000
AS94_05960	molybdenum cofactor biosynthesis protein C	-1.334	0.000	0.000
AS94_05965	molybdenum cofactor biosynthesis protein B	-1.780	0.000	0.000
AS94_05970	molybdopterin biosynthesis protein MoeB	-1.371	0.000	0.000

AS94_05975	molybdenum ABC transporter ATP-binding protein	-1.366	0.000	0.000
AS94_05980	molybdenum ABC transporter permease	-1.152	0.000	0.000
AS94_05985	molybdenum ABC transporter substrate-binding protein	-0.692	0.000	0.000
AS94_05990	formate dehydrogenase subunit D	-0.503	0.001	0.003
AS94_06005	purine nucleosidase	-0.457	0.025	0.050
AS94_06010	ferrichrome ABC transporter substrate-binding protein	-0.679	0.000	0.000
AS94_06015	acyl-CoA dehydrogenase	-0.545	0.001	0.001
AS94_06020	urea transporter	-1.505	0.000	0.000
AS94_06025	urease subunit gamma	-1.562	0.005	0.011
AS94_06030	urease subunit beta	-1.691	0.000	0.001
AS94_06035	urease subunit alpha	-1.091	0.000	0.000
AS94_06040	urease accessory protein UreE	-1.128	0.000	0.000
AS94_06045	urease accessory protein UreF	-1.047	0.000	0.000
AS94_06050	urease accessory protein UreG	-1.179	0.000	0.000
AS94_06055	urease accessory protein UreD	-1.412	0.000	0.000
AS94_06060	MarR family transcriptional regulator	-0.661	0.000	0.000
AS94_06065	hypothetical protein	-0.937	0.000	0.000
AS94_06070	transcriptional regulator	-0.659	0.018	0.038
AS94_06100	hypothetical protein	-0.665	0.000	0.000
AS94_06110	2-hydroxyacid dehydrogenase	-1.462	0.000	0.000
AS94_06115	hypothetical protein	-0.734	0.000	0.000
AS94_06130	hypothetical protein	-0.935	0.000	0.000
AS94_06135	oxidoreductase	-0.524	0.000	0.000
AS94_06155	DeoR family transcriptional regulator	-0.879	0.000	0.000
AS94_06195	HAD family hydrolase	-0.592	0.005	0.012
AS94_06200	sodium transporter	-0.658	0.008	0.019
AS94_06210	PTS alpha-glucoside transporter subunit IIBC	-2.524	0.000	0.000
AS94_06225	sodium:proton antiporter	-0.460	0.003	0.008
AS94_06235	oxidoreductase	-0.298	0.012	0.026

AS94_06265	lysostaphin resistance protein A	-0.451	0.001	0.002
AS94_06270	ribose 5-phosphate isomerase	-0.857	0.000	0.000
AS94_06275	molybdenum cofactor biosysynthesis protein	-0.550	0.002	0.006
AS94_06280	aldose 1-epimerase	-0.555	0.000	0.001
AS94_06285	membrane protein	-0.764	0.001	0.001
AS94_06305	3-methyladenine DNA glycosylase	-1.074	0.000	0.000
AS94_06315	isopentenyl pyrophosphate isomerase	-0.523	0.000	0.000
AS94_06380	hemin ABC transporter ATP-binding protein	-2.343	0.000	0.000
AS94_06385	hemin ABC transporter permease	-2.331	0.000	0.000
AS94_06390	heme transporter CcmC	-0.866	0.001	0.003
AS94_06395	sensor histidine kinase	-1.154	0.000	0.000
AS94_06420	antibiotic ABC transporter permease	-0.928	0.003	0.007
AS94_06445	acetyltransferase	-0.846	0.001	0.002
AS94_06460	GNAT family acetyltransferase	-0.981	0.000	0.000
AS94_06465	ferredoxin--NADP reductase	-1.499	0.000	0.000
AS94_06490	PTS sucrose transporter subunit IIBC	-2.511	0.000	0.000
AS94_06550	LuxR family transcriptional regulator	-0.772	0.000	0.000
AS94_06555	sensor histidine kinase	-0.758	0.000	0.000
AS94_06560	nreA protein	-0.869	0.000	0.000
AS94_06565	nitrate reductase subunit gamma	-0.879	0.000	0.000
AS94_06570	nitrate reductase sununit delta	-1.490	0.000	0.000
AS94_06575	nitrate reductase	-0.998	0.000	0.000
AS94_06580	nitrate reductase	-1.111	0.000	0.000
AS94_06585	uroporphyrinogen III methyltransferase	-1.230	0.000	0.000
AS94_06590	nitrite reductase NAD(P)H small subunit	-0.916	0.000	0.001
AS94_06595	nitrite reductase	-1.216	0.000	0.000
AS94_06600	cobalamin biosynthesis protein CbiX	-1.105	0.000	0.000
AS94_06625	zinc ABC transporter substrate-binding protein	-0.421	0.007	0.016
AS94_06630	protein-disulfide isomerase	-0.766	0.000	0.000
AS94_06635	hypothetical protein	-0.755	0.003	0.008

AS94_06665	hypothetical protein	-1.160	0.001	0.002
AS94_06745	hypothetical protein	-1.320	0.000	0.001
AS94_06765	bicyclomycin transporter TcaB	-0.428	0.001	0.003
AS94_06775	hypothetical protein	-0.705	0.000	0.000
AS94_06785	alanine glycine permease	-0.741	0.000	0.000
AS94_06795	amino acid permease	-1.054	0.000	0.000
AS94_06805	epimerase	-0.584	0.000	0.000
AS94_06810	2-dehydropantoate 2-reductase	-1.502	0.000	0.000
AS94_06815	quinolone resistance protein NorB	-0.820	0.000	0.000
AS94_06820	amino acid ABC transporter permease	-1.528	0.000	0.000
AS94_06825	glycine/betaine ABC transporter substrate-binding protein	-1.462	0.000	0.000
AS94_06830	choline ABC transporter permease	-0.926	0.000	0.000
AS94_06835	glycine/betaine ABC transporter ATP-binding protein	-1.096	0.000	0.000
AS94_06840	hypothetical protein	-0.818	0.000	0.000
AS94_06845	amino acid:proton symporter	-0.696	0.000	0.000
AS94_06860	membrane protein	-0.668	0.000	0.000
AS94_06870	peptidase M28	-1.188	0.000	0.000
AS94_06875	hypothetical protein	-0.995	0.000	0.000
AS94_06910	nickel ABC transporter permease	-0.681	0.024	0.049
AS94_06915	nickel ABC transporter substrate-binding protein	-0.836	0.000	0.000
AS94_06985	hypothetical protein	-1.655	0.000	0.000
AS94_06990	hypothetical protein	-0.980	0.000	0.000
AS94_06995	hypothetical protein	-1.651	0.000	0.000
AS94_07000	hypothetical protein	-0.944	0.000	0.000
AS94_07010	hypothetical protein	-0.929	0.000	0.000
AS94_07025	helicase	-0.278	0.024	0.049
AS94_07050	UTP--glucose-1-phosphate uridylyltransferase	-0.864	0.000	0.000
AS94_07055	fibronectin-binding protein A	-0.675	0.000	0.000
AS94_07065	gluconate permease	-1.314	0.000	0.000

AS94_07070	gluconokinase	-0.704	0.000	0.000
AS94_07075	GntR family transcriptional regulator	-0.581	0.000	0.001
AS94_07080	MerR family transcriptional regulator	-1.248	0.000	0.000
AS94_07120	fructose-1_6-bisphosphatase	-0.684	0.000	0.000
AS94_07130	carboxylesterase	-1.216	0.000	0.000
AS94_07135	glyoxalase	-0.813	0.000	0.000
AS94_07140	MarR family transcriptional regulator	-1.298	0.000	0.000
AS94_07145	acetyltransferase	-0.474	0.006	0.015
AS94_07150	glyoxalase	-0.476	0.003	0.008
AS94_07155	NAD(P)H nitroreductase	-0.572	0.000	0.000
AS94_07165	HAD family hydrolase	-0.690	0.000	0.001
AS94_07215	esterase	-1.153	0.000	0.000
AS94_07225	acyl-CoA thioester hydrolase	-1.124	0.000	0.000
AS94_07230	PTS glucose transporter subunit IIABC	-1.180	0.000	0.000
AS94_07235	pyruvate oxidase	-0.789	0.000	0.000
AS94_07240	holin	-0.434	0.000	0.001
AS94_07245	holin	-2.360	0.000	0.000
AS94_07265	hydroxymethylglutaryl-CoA reductase	-0.342	0.008	0.018
AS94_07275	methylated DNA-protein cysteine S-methyltransferase	-0.767	0.000	0.000
AS94_07315	maltose O-acetyltransferase	-0.514	0.023	0.047
AS94_07325	ATPase P	-0.536	0.001	0.002
AS94_07395	TetR family transcriptional regulator	-0.747	0.001	0.002
AS94_07475	fructosamine-3-kinase	-0.562	0.000	0.000
AS94_07510	aspartate decarboxylase	-0.527	0.001	0.003
AS94_07515	pantoate--beta-alanine ligase	-0.395	0.003	0.006
AS94_07520	3-methyl-2-oxobutanoate hydroxymethyltransferase	-0.444	0.005	0.012
AS94_07530	alpha-acetolactate decarboxylase	-0.837	0.000	0.000
AS94_07555	fructose-bisphosphate aldolase	-0.487	0.000	0.000
AS94_07560	malate:quinone oxidoreductase	-0.437	0.000	0.000
AS94_07570	acyl--CoA ligase	-0.676	0.000	0.000
AS94_07590	betaine-aldehyde dehydrogenase	-0.445	0.003	0.008

AS94_07595	hypothetical protein	-0.387	0.019	0.040
AS94_07600	choline transporter BetT	-0.433	0.008	0.017
AS94_07605	ribonucleoside-triphosphate reductase activatingprotein	-0.633	0.000	0.000
AS94_07610	ribonucleoside triphosphate reductase	-0.347	0.020	0.041
AS94_07620	precorrin-2 dehydrogenase	-0.640	0.000	0.000
AS94_07625	sulfite reductase subunit alpha	-0.583	0.000	0.000
AS94_07635	peptide ABC transporter permease	-0.610	0.000	0.001
AS94_07675	tributyrin esterase	-0.817	0.000	0.000
AS94_07685	hypothetical protein	-0.997	0.001	0.002
AS94_07690	type I restriction endonuclease subunit S	-0.999	0.000	0.000
AS94_07700	RNA helicase	-0.925	0.000	0.000
AS94_07710	hypothetical protein	-0.768	0.000	0.000
AS94_07715	hypothetical protein	-1.180	0.000	0.000
AS94_07720	transposase	-0.856	0.000	0.000
AS94_07800	Vitamin B12 ABC transporter substrate-binding protein	-0.665	0.000	0.000
AS94_07805	enterobactin ABC transporter permease	-0.592	0.003	0.008
AS94_07810	HAD family hydrolase	-0.757	0.000	0.001
AS94_07815	alpha/beta hydrolase	-0.934	0.000	0.000
AS94_07820	hypothetical protein	-1.127	0.000	0.000
AS94_07925	N-acetylmannosaminyltransferase	-0.491	0.004	0.009
AS94_07930	teichoic acid ABC transporter ATP-binding protein	-0.445	0.000	0.001
AS94_07965	nucleoside permease	-0.894	0.000	0.000
AS94_07975	iron-dicitrate ABC transporter ATP-binding protein	-1.143	0.000	0.000
AS94_07980	ferrichrome ABC transporter permease	-1.020	0.000	0.000
AS94_07985	iron ABC transporter permease	-1.089	0.000	0.000
AS94_07990	dihydroxyacetone kinase subunit K	-0.370	0.005	0.013
AS94_07995	dihydroxyacetone kinase subunit L	-0.684	0.000	0.000
AS94_08000	PTS mannnose transporter subunit IIA	-1.387	0.000	0.000
AS94_08005	hypothetical protein	-0.456	0.000	0.001

AS94_08025	acetyltransferase	-0.866	0.000	0.000
AS94_08030	3-beta hydroxysteroid dehydrogenase	-0.466	0.001	0.004
AS94_08050	bacitracin ABC transporter permease	-0.704	0.000	0.000
AS94_08065	peptidase M23B	-0.353	0.003	0.007
AS94_08070	inhibitor of apoptosis-promoting Bax1	-0.713	0.000	0.000
AS94_08075	AraC family transcriptional regulator	-0.876	0.000	0.000
AS94_08080	MarR family transcriptional regulator	-3.088	0.000	0.000
AS94_08095	hypothetical protein	-0.639	0.000	0.000
AS94_08100	LysR family transcriptional regulator	-0.574	0.000	0.000
AS94_08105	sugar MFS transporter	-0.567	0.000	0.000
AS94_08170	MarR family transcriptional regulator	-0.265	0.008	0.019
AS94_08195	deoxyribodipyrimidine photo-lyase	-0.283	0.011	0.024
AS94_08220	hypothetical protein	-0.499	0.007	0.017
AS94_08225	YbaK/EbsC protein	-1.377	0.000	0.000
AS94_08230	DeoR family transcriptional regulator	-0.660	0.001	0.003
AS94_08235	phosphofructokinase	-0.829	0.000	0.000
AS94_08240	PTS fructose transporter subunit IIC	-0.562	0.000	0.000
AS94_08280	hypothetical protein	-0.403	0.004	0.009
AS94_08335	heme ABC transporter ATP-binding protein	-0.953	0.000	0.000
AS94_08340	ATP-dependent DNA helicase RecQ	-1.423	0.000	0.000
AS94_08370	lipid kinase	-0.310	0.006	0.015
AS94_08390	ribonucleotide reductase stimulatory protein	-1.282	0.000	0.000
AS94_08395	ribonucleotide-diphosphate reductase subunit alpha	-0.892	0.000	0.000
AS94_08405	iron ABC transporter permease	-1.854	0.000	0.000
AS94_08410	iron ABC transporter permease	-1.730	0.000	0.000
AS94_08415	iron ABC transporter ATP-binding protein	-1.111	0.000	0.000
AS94_08420	iron ABC transporter substrate-binding protein	-0.488	0.005	0.012
AS94_08485	hypothetical protein	-1.288	0.000	0.000
AS94_08530	excinuclease ABC subunit B	-0.825	0.000	0.000

AS94_08565	hypothetical protein	-0.438	0.019	0.040
AS94_08620	glyceraldehyde-3-phosphate dehydrogenase	-0.363	0.002	0.004
AS94_08635	phosphoglyceromutase	-0.365	0.004	0.009
AS94_08640	enolase	-0.535	0.000	0.000
AS94_08670	hypothetical protein	-0.495	0.000	0.000
AS94_08715	virulence-associated protein E	-0.734	0.002	0.005
AS94_08755	pathogenicity island protein	-0.649	0.000	0.000
AS94_08855	serine phosphatase	-0.461	0.000	0.000
AS94_08860	PemK family transcriptional regulator	-0.446	0.004	0.011
AS94_08870	alanine racemase	-0.386	0.002	0.004
AS94_08895	potassium-transporting ATPase subunit C	-1.023	0.000	0.000
AS94_08900	potassium-transporting ATPase subunit B	-0.653	0.003	0.007
AS94_08915	histidine kinase	-0.357	0.020	0.041
AS94_08975	thiamine-phosphate pyrophosphorylase	-0.830	0.007	0.015
AS94_08980	hydroxyethylthiazole kinase	-0.881	0.013	0.027
AS94_09000	single-stranded DNA-binding protein	-1.486	0.000	0.000
AS94_09005	hypothetical protein	-0.490	0.013	0.029
AS94_09140	fructose-bisphosphate aldolase	-0.803	0.000	0.000
AS94_09145	hypothetical protein	-0.534	0.004	0.009
AS94_09170	hypothetical protein	-0.457	0.001	0.003
AS94_09190	membrane protein	-1.187	0.000	0.000
AS94_09195	pyrimidine-nucleoside phosphorylase	-1.176	0.000	0.000
AS94_09200	deoxyribose-phosphate aldolase	-0.624	0.000	0.000
AS94_09210	general stress protein	-1.882	0.000	0.000
AS94_09295	FmtB protein	-1.108	0.000	0.000
AS94_09375	MarR family transcriptional regulator	-0.887	0.000	0.000
AS94_09385	siderophore ABC transporter permease	-1.180	0.003	0.007
AS94_09390	iron ABC transporter substrate-binding protein	-1.246	0.000	0.000
AS94_09415	siderophore biosynthesis protein SbnE	-0.589	0.020	0.041
AS94_09455	acetoin reductase	-1.696	0.000	0.000
AS94_09485	superoxide dismutase	-2.825	0.000	0.000

AS94_09515	phosphopentomutase	-0.774	0.000	0.000
AS94_09545	2'_ 3'-cyclic nucleotide 2'-phosphodiesterase	-1.076	0.000	0.000
AS94_09555	bifunctional acetaldehyde-CoA/alcohol dehydrogenase	-0.539	0.000	0.000
AS94_09640	monooxygenase IsdI	-0.956	0.016	0.034
AS94_09645	hypothetical protein	-1.375	0.000	0.000
AS94_09700	4'-phosphopantetheinyl transferase	-0.897	0.000	0.000
AS94_09705	hypothetical protein	-1.027	0.000	0.000
AS94_09735	isochorismatase	-0.795	0.000	0.000
AS94_09740	pyruvate decarboxylase	-0.706	0.000	0.000
AS94_09750	PTS glucose transporter subunit IIABC	-1.301	0.000	0.000
AS94_09785	ABC transporter ATP-binding protein	-1.287	0.000	0.000
AS94_09815	azoreductase	-1.128	0.000	0.000
AS94_09825	sugar ABC transporter ATP-binding protein	-1.195	0.000	0.000
AS94_09830	ABC transporter substrate-binding protein	-1.413	0.000	0.000
AS94_09835	arabinogalactan ABC transporter permease	-1.200	0.000	0.000
AS94_09840	arabinogalactan ABC transporter permease	-1.501	0.000	0.000
AS94_09845	oxidoreductase	-2.229	0.000	0.000
AS94_09850	NADH-dependent dehydrogenase	-2.559	0.000	0.000
AS94_09855	xylose isomerase	-2.694	0.000	0.000
AS94_09870	AraC family transcriptional regulator	-1.041	0.000	0.000
AS94_09885	formate acetyltransferase	-2.478	0.000	0.000
AS94_09890	pyruvate formate lyase-activating protein	-2.527	0.000	0.000
AS94_09895	hypothetical protein	-1.630	0.000	0.000
AS94_09900	glycerophosphoryl diester phosphodiesterase	-0.558	0.000	0.001
AS94_09980	nitric oxide dioxygenase	-0.698	0.000	0.000
AS94_09990	PTS glucose transporter subunit IIB	-1.095	0.000	0.000
AS94_10020	sorbitol dehydrogenase	-0.901	0.006	0.014
AS94_10030	iditol 2-dehydrogenase	-1.095	0.000	0.000
AS94_10075	cell wall biosynthesis protein ScdA	-0.712	0.000	0.000

AS94_10080	sensor histidine kinase	-0.941	0.000	0.000
AS94_10085	LytR family transcriptional regulator	-0.828	0.000	0.001
AS94_10090	murein hydrolase regulator LrgA	-0.780	0.000	0.000
AS94_10100	GntR family transcriptional regulator	-0.750	0.000	0.000
AS94_10105	phosphoenolpyruvate-dependent sugar PTS family porter_ EIIA 1	-2.185	0.000	0.000
AS94_10110	aryl-phospho-beta-D-glucosidase	-1.718	0.000	0.000
AS94_10115	hypothetical protein	-1.156	0.000	0.000
AS94_10140	LacI family transcriptional regulator	-1.287	0.000	0.000
AS94_10155	penicillin V acylase	-0.996	0.000	0.000
AS94_10255	hypothetical protein	-0.598	0.000	0.001
AS94_10320	formate/nitrite transporter	-0.783	0.000	0.000
AS94_10330	5'-nucleotidase	-0.675	0.001	0.001
AS94_10355	pseudouridine-5'-phosphate glycosidase	-0.803	0.000	0.000
AS94_10365	sialic acid transporter	-1.527	0.000	0.000
AS94_10370	N-acetylneuraminate lyase	-1.747	0.000	0.000
AS94_10375	N-acetylmannosamine kinase	-0.868	0.000	0.000
AS94_10380	RpiR family transcriptional regulator	-0.609	0.000	0.000
AS94_10385	N-acetylmannosamine-6-phosphate 2-epimerase	-0.821	0.000	0.000
AS94_10400	alpha/beta hydrolase	-0.831	0.000	0.000
AS94_10455	MarR family transcriptional regulator	-0.503	0.007	0.017
AS94_10475	glyoxalase	-0.559	0.009	0.020
AS94_10480	luciferase	-1.058	0.000	0.000
AS94_10485	FMN reductase	-0.591	0.015	0.032
AS94_10490	membrane protein	-0.960	0.000	0.000
AS94_10665	hypothetical protein	-1.408	0.000	0.000
AS94_10675	phosphoglycerate mutase	-0.449	0.012	0.026
AS94_10685	hypothetical protein	-0.397	0.003	0.008
AS94_10700	alkyl hydroperoxide reductase subunit C	-0.477	0.000	0.000
AS94_10705	NADPH-dependent oxidoreductase	-0.566	0.000	0.001
AS94_10730	xanthine phosphoribosyltransferase	-1.458	0.000	0.000
AS94_10735	xanthine permease	-1.145	0.000	0.000

AS94_10740	inosine 5'-monophosphate dehydrogenase	-1.063	0.000	0.000
AS94_10745	GMP synthase	-0.806	0.000	0.000
AS94_10815	membrane protein	-1.762	0.000	0.000
AS94_10820	3-beta hydroxysteroid dehydrogenase	-1.256	0.000	0.000
AS94_10890	hypothetical protein	-0.978	0.000	0.000
AS94_10895	hypothetical protein	-1.471	0.003	0.006
AS94_10940	NADH dehydrogenase subunit 5	-0.803	0.000	0.000
AS94_10945	hypothetical protein	-0.713	0.000	0.000
AS94_10950	hypothetical protein	-0.447	0.001	0.002
AS94_10965	carboxylesterase	-0.830	0.000	0.000
AS94_10970	sodium-dependent transporter	-0.471	0.008	0.017
AS94_11000	N-acetylmuramoyl-L-alanine amidase	-0.384	0.001	0.002
AS94_11020	hypothetical protein	-0.830	0.000	0.000
AS94_11025	hypothetical protein	-0.701	0.000	0.000
AS94_11050	PTS ascorbate transporter subunit IIA	-0.806	0.000	0.000
AS94_11075	DNA polymerase III subunit gamma/tau	-0.323	0.006	0.014
AS94_11080	hypothetical protein	-0.618	0.002	0.005
AS94_11090	adhesin	-1.569	0.000	0.000
AS94_11095	2-oxoglutarate translocator	-0.887	0.000	0.000
AS94_11100	chloramphenicol-sensitive protein RarD	-0.445	0.009	0.020
AS94_11110	nickel transporter NixA	-0.701	0.000	0.000
AS94_11115	N-acetyltransferase	-0.617	0.000	0.000
AS94_11145	cold-shock protein	-0.635	0.000	0.000
AS94_11150	Cro/Cl family transcriptional regulator	-1.114	0.000	0.000
AS94_11155	hypothetical protein	-1.053	0.000	0.000
AS94_11160	hypothetical protein	-1.163	0.000	0.000
AS94_11225	carbohydrate kinase	-1.327	0.000	0.000
AS94_11265	50S ribosomal protein L9	-0.585	0.000	0.000
AS94_11270	replicative DNA helicase	-0.915	0.000	0.000
AS94_11400	recombinase RecA	-0.609	0.002	0.005
AS94_11465	branched-chain amino acid aminotransferase	-0.377	0.000	0.001
AS94_11480	molecular chaperone	-0.896	0.000	0.000

AS94_11495	elongation factor Tu	-0.311	0.006	0.015
AS94_11520	DNA-directed RNA polymerase subunit beta'	-0.414	0.000	0.000
AS94_11525	DNA-directed RNA polymerase subunit beta	-0.292	0.009	0.019
AS94_11600	glutamyl-tRNA synthase	-0.584	0.000	0.000
AS94_11645	pyridoxal biosynthesis protein	-0.347	0.004	0.009
AS94_11660	adhesin	-2.593	0.000	0.000
AS94_11670	pyrrolidone-carboxylate peptidase	-1.024	0.000	0.000
AS94_11690	polysaccharide deacetylase	-1.341	0.000	0.000
AS94_11695	ATP phosphoribosyltransferase	-2.684	0.000	0.000
AS94_11700	ATP phosphoribosyltransferase	-2.917	0.000	0.000
AS94_11705	histidinol dehydrogenase	-2.248	0.000	0.000
AS94_11710	histidinol-phosphate aminotransferase	-2.002	0.000	0.000
AS94_11715	imidazoleglycerol-phosphate dehydratase	-1.603	0.009	0.020
AS94_11720	imidazole glycerol phosphate synthase	-1.820	0.001	0.002
AS94_11725	1-(5-phosphoribosyl)-5-[(5-phosphoribosylamino)methylideneamino] imidazole-4-carboxamide isomerase	-1.342	0.000	0.001
AS94_11730	imidazole glycerol phosphate synthase	-0.959	0.004	0.010
AS94_11735	phosphoribosyl-AMP cyclohydrolase	-0.876	0.000	0.000
AS94_11760	N-glycosyltransferase	-1.459	0.000	0.000
AS94_11830	adhesin	-0.661	0.000	0.000
AS94_11835	preprotein translocase subunit SecY	-1.029	0.000	0.001
AS94_11840	Accessory Sec system protein Asp1	-1.428	0.000	0.000
AS94_11845	accessory secretory protein Asp2	-1.126	0.000	0.000
AS94_11850	Accessory Sec system protein Asp3	-0.998	0.001	0.002
AS94_11890	mannose-6-phosphate isomerase	-2.854	0.000	0.000
AS94_11895	PTS mannose transporter subunit IIABC	-2.655	0.000	0.000
AS94_11905	hypothetical protein	-0.654	0.001	0.004
AS94_11910	hypothetical protein	-0.409	0.001	0.001
AS94_11915	aureolysin	-0.799	0.000	0.001
AS94_11920	ArgR family transcriptional regulator	-0.767	0.002	0.005

AS94_11925		arginine deiminase	-1.650	0.000	0.000
AS94_11930		ornithine carbamoyltransferase	-0.787	0.000	0.000
AS94_11935		amino acid APC transporter	-0.309	0.023	0.046
AS94_11960		Fur family transcriptional regulator	-0.743	0.000	0.000
AS94_11980		membrane protein	-0.302	0.021	0.042
AS94_12005		membrane protein	-0.503	0.002	0.004
AS94_12380		hypothetical protein	-0.881	0.000	0.000
AS94_12470		ferritin	-0.759	0.000	0.000
AS94_12565		membrane protein	-0.577	0.000	0.001
AS94_12570		NAD synthetase	-0.437	0.002	0.006
AS94_12575		nicotinate phosphoribosyltransferase	-0.365	0.011	0.025
AS94_12585		prephenate dehydratase	-0.689	0.000	0.000
AS94_12605		pyrophosphatase	-0.684	0.000	0.000
AS94_12610		aldehyde dehydrogenase	-0.768	0.000	0.000
AS94_12745		phospholipase	-0.832	0.001	0.001
AS94_12785		hypothetical protein	-1.400	0.000	0.000
AS94_12815		peptidase M23	-0.821	0.000	0.001
AS94_12825		gamma-hemolysin subunit B	-0.743	0.001	0.001
AS94_12865		nitroreductase	-0.506	0.000	0.000
AS94_12875	*hld*	delta-hemolysin	-0.725	0.000	0.000
AS94_12880	*agrB*	accessory gene regulator B	-0.893	0.000	0.000
AS94_12890	*agrC*	histidine kinase	-0.607	0.000	0.000
AS94_12895	*agrA*	histidine kinase	-0.952	0.000	0.000
AS94_12900		fructokinase	-0.915	0.000	0.000
AS94_12905		sucrose-6-phosphate hydrolase	-0.487	0.000	0.000
AS94_12945		O-sialoglycoprotein endopeptidase	-0.317	0.011	0.024
AS94_13015		hypothetical protein	-3.718	0.007	0.017
AS94_13445		protoporphyrinogen oxidase	-0.334	0.021	0.043
AS94_13475		cell-cycle regulation protein HIT	-1.061	0.000	0.000
AS94_12245		hypothetical protein	-2.279	0.000	0.001
AS94_12265	ϕSA169	hypothetical protein	-0.776	0.016	0.033
AS94_12285		DNA replication protein DnaC	-0.520	0.008	0.018
AS94_13030	mutual prophage	hypothetical protein	-1.362	0.000	0.000

AS94_13035	enterotoxin	-0.920	0.000	0.000
AS94_13070	autolysin	-2.341	0.000	0.000
AS94_13075	holin	-3.249	0.000	0.000
AS94_13080	hypothetical protein	-2.507	0.000	0.000
AS94_13090	hypothetical protein	-2.399	0.000	0.000
AS94_13095	hypothetical protein	-2.348	0.000	0.000
AS94_13100	minor structural protein	-2.067	0.000	0.000
AS94_13105	hypothetical protein	-2.466	0.008	0.018
AS94_13110	peptidase	-2.176	0.000	0.000
AS94_13115	holin	-2.245	0.000	0.000
AS94_13120	tail protein	-2.112	0.000	0.000
AS94_13130	hypothetical protein	-2.801	0.000	0.000
AS94_13135	tail protein	-2.071	0.000	0.000
AS94_13140	tail protein	-2.123	0.000	0.000
AS94_13145	hypothetical protein	-2.075	0.004	0.010
AS94_13150	hypothetical protein	-1.718	0.000	0.000
AS94_13155	hypothetical protein	-1.746	0.006	0.014
AS94_13160	hypothetical protein	-2.242	0.000	0.000
AS94_13165	phage capsid protein	-2.095	0.000	0.000
AS94_13170	ATP-dependent Clp protease ClpP	-2.070	0.000	0.000
AS94_13175	portal protein	-1.790	0.000	0.000
AS94_13180	terminase	-1.797	0.000	0.000
AS94_13185	terminase	-1.594	0.000	0.000
AS94_13190	HNH endonuclease	-1.492	0.001	0.004
AS94_13195	transcriptional regulator	-1.929	0.000	0.000
AS94_13200	helicase	-1.557	0.000	0.000
AS94_13205	hypothetical protein	-1.435	0.000	0.000
AS94_13220	hypothetical protein	-1.711	0.000	0.000
AS94_13235	Dimeric dUTPase	-0.916	0.000	0.001
AS94_13350	hypothetical protein	-1.164	0.007	0.015
AS94_13355	antirepressor	-0.741	0.000	0.000
AS94_13360	hypothetical protein	-1.727	0.000	0.000
AS94_13370	hypothetical protein	-0.962	0.000	0.000
AS94_13375	XRE family transcriptional regulator	-1.445	0.000	0.000

AS94_13390	hypothetical protein	-0.899	0.000	0.000
