# Supplementary material for: Transcriptome Analyses of Prophage in Mediating Persistent Methicillin-Resistant Staphylococcus aureus Endovascular Infection
Source: Genes (Basel). 2022 Aug 25;13(9):1527. doi: 10.3390/genes13091527 (PMC9498598; doi:10.3390/genes13091527)
Supplement: Supplementary file 1 [file genes-13-01527-s001.zip › Table S5.pdf]

Table S5. Up-regulated DEGs in 300-169 vs. 301-188::ϕSA169

locus	gene	group	product	log_2(fold change)	*p* value	*p* adj
AS94_00015			hypothetical protein	0.660	0.003	0.007
AS94_00025			calcium-binding protein	1.736	0.000	0.000
AS94_00060			phosphoenolpyruvate carboxykinase	1.067	0.000	0.000
AS94_00150			Diaminohydroxyphosphori-bosylaminopyrimidine deaminase	0.875	0.000	0.000
AS94_00155			riboflavin synthase subunit alpha	0.706	0.001	0.002
AS94_00160			GTP cyclohydrolase	0.537	0.001	0.002
AS94_00170			proline dehydrogenase	1.615	0.000	0.000
AS94_00175			lysophospholipase	0.756	0.000	0.000
AS94_00180			MarR family transcriptional regulator	0.865	0.000	0.000
AS94_00275			hypothetical protein	0.824	0.000	0.000
AS94_00280			tRNA-binding protein	1.016	0.000	0.000
AS94_00285			cell division protein FtsK	1.082	0.000	0.000
AS94_00290			UDP-N-acetylmuramate--alanine ligase	0.757	0.000	0.000
AS94_00295			hypothetical protein	0.733	0.000	0.000
AS94_00300		host genes	smooth muscle caldesmon	1.244	0.000	0.000
AS94_00315			histone deacetylase	0.387	0.016	0.035
AS94_00325			acetyl-CoA synthetase	1.010	0.000	0.000
AS94_00330			formate--tetrahydrofolate ligase	0.454	0.000	0.000
AS94_00350			serine protease	1.577	0.000	0.000
AS94_00390			30S ribosomal protein S4	0.373	0.001	0.002
AS94_00400			septation ring formation regulator EzrA	0.257	0.014	0.031
AS94_00430			acetate kinase	0.426	0.000	0.000
AS94_00440			alanine dehydrogenase	0.949	0.000	0.000
AS94_00480			oligoribonuclease	0.299	0.018	0.039
AS94_00520			citrate synthase	0.281	0.012	0.027
AS94_00530			PhoP family transcriptional regulator	0.419	0.007	0.016
AS94_00560			glyceraldehyde-3-phosphate dehydrogenase	1.143	0.000	0.000
AS94_00590			translation initiation factor IF-3	1.000	0.000	0.000
AS94_00595			50S ribosomal protein L35	1.059	0.000	0.000
AS94_00600			50S ribosomal protein L20	1.117	0.000	0.000

AS94_00605	DNA mismatch repair protein MutT	0.660	0.001	0.002
AS94_00615	trigger factor	0.854	0.000	0.000
AS94_00620	ATP-dependent protease	0.503	0.000	0.000
AS94_00625	GTP-binding protein YsxC	0.661	0.001	0.002
AS94_00630	glutamyl-tRNA reductase	0.704	0.000	0.000
AS94_00635	cytochrome C assembly protein	0.951	0.000	0.000
AS94_00640	porphobilinogen deaminase	0.989	0.000	0.000
AS94_00660	aminopeptidase	0.487	0.000	0.001
AS94_00700	membrane protein	4.341	0.000	0.000
AS94_00705	hypothetical protein	4.081	0.000	0.000
AS94_00710	rod shape-determining protein MreC	0.883	0.000	0.000
AS94_00715	rod shape-determining protein MreD	0.811	0.000	0.000
AS94_00760	queuine tRNA-ribosyltransferase	0.340	0.006	0.014
AS94_00815	hypothetical protein	0.284	0.024	0.050
AS94_00825	Rrf2 family transcriptional regulator	0.524	0.000	0.001
AS94_00830	hypothetical protein	1.334	0.000	0.000
AS94_00910	allophanate hydrolase subunit 1	2.336	0.000	0.000
AS94_00915	allophanate hydrolase subunit 2	2.139	0.000	0.000
AS94_00920	acetyl-CoA carboxylase	1.965	0.000	0.000
AS94_00925	acetyl-CoA carboxylase biotin carboxylase subunit	2.097	0.000	0.000
AS94_00930	hypothetical protein	2.053	0.000	0.000
AS94_00935	iron transporter	1.804	0.000	0.000
AS94_00955	S-adenosylhomocysteine nucleosidase	1.166	0.000	0.000
AS94_00960	hypothetical protein	1.194	0.000	0.000
AS94_00965	GTPase	1.265	0.000	0.000
AS94_00970	shikimate 5-dehydrogenase	1.269	0.000	0.000
AS94_00980	nicotinic acid mononucleotide adenylyltransferase	1.241	0.000	0.000
AS94_00985	HAD family hydrolase	1.207	0.000	0.000
AS94_00990	Iojap family protein	1.229	0.000	0.000
AS94_00995	methyltransferase	0.906	0.000	0.000
AS94_01020	30S ribosomal protein S20	0.520	0.000	0.000
AS94_01035	HrcA family transcriptional regulator	0.522	0.000	0.000
AS94_01040	heat shock protein GrpE	0.650	0.000	0.000
AS94_01045	molecular chaperone DnaK	0.494	0.000	0.000

AS94_01050	molecular chaperone DnaJ	0.961	0.000	0.000
AS94_01055	ribosomal protein L11 methyltransferase	1.068	0.000	0.000
AS94_01060	16S rRNA methyltransferase	0.843	0.000	0.000
AS94_01070	30S ribosomal protein S21	0.488	0.001	0.003
AS94_01075	serine protease	0.870	0.000	0.000
AS94_01080	hypothetical protein	0.458	0.000	0.000
AS94_01085	iron transporter	0.510	0.000	0.000
AS94_01095	rRNA maturation factor	0.451	0.006	0.014
AS94_01110	GTPase Era	0.359	0.003	0.008
AS94_01120	glycyl-tRNA synthetase	1.346	0.000	0.000
AS94_01130	phosphotransferase	0.428	0.001	0.003
AS94_01145	SAM-dependent methyltransferase	0.488	0.004	0.010
AS94_01190	penicillin-binding protein 3	0.428	0.000	0.001
AS94_01195	50S ribosomal protein L33	0.679	0.005	0.011
AS94_01200	5-formyltetrahydrofolate cyclo-ligase	0.881	0.000	0.000
AS94_01205	membrane protein	0.926	0.000	0.000
AS94_01215	glucokinase	0.797	0.000	0.000
AS94_01220	hypothetical protein	0.789	0.003	0.006
AS94_01225	hydroxyacylglutathione hydrolase	0.588	0.000	0.000
AS94_01305	elongation factor P	0.499	0.000	0.000
AS94_01310	acetyl-CoA carboxylase	0.721	0.000	0.000
AS94_01315	acetyl-CoA carboxylase biotin carboxylase subunit	0.754	0.000	0.000
AS94_01320	hypothetical protein	0.689	0.000	0.000
AS94_01325	transcription antitermination protein NusB	0.624	0.000	0.001
AS94_01330	exodeoxyribonuclease VII large subunit	0.719	0.000	0.000
AS94_01340	geranyltranstransferase	0.730	0.000	0.000
AS94_01345	arginine repressor ArgR	1.479	0.000	0.000
AS94_01350	DNA repair protein RecN	1.672	0.000	0.000
AS94_01355	dihydrolipoamide dehydrogenase	0.839	0.000	0.000
AS94_01375	hypothetical protein	0.615	0.001	0.002
AS94_01380	membrane protein	0.468	0.001	0.004
AS94_01485	PhoP family transcriptional regulator	0.788	0.000	0.000
AS94_01495	hypothetical protein	1.538	0.000	0.000
AS94_01500	hypothetical protein	1.402	0.000	0.000

AS94_01505	hypothetical protein	0.680	0.001	0.004
AS94_01520	hypothetical protein	0.543	0.003	0.008
AS94_01525	ATP-dependent DNA helicase RecQ	0.528	0.001	0.002
AS94_01530	peptidoglycan-binding protein LysM	0.307	0.004	0.009
AS94_01550	30S ribosomal protein S1	0.572	0.000	0.000
AS94_01580	heptaprenyl diphosphate synthase subunit II	0.302	0.011	0.025
AS94_01585	nucleoside diphosphate kinase	0.908	0.000	0.000
AS94_01655	asparaginyl-tRNA synthase	0.647	0.000	0.000
AS94_01675	transglycosylase	0.938	0.000	0.000
AS94_01680	recombinase RecU	0.957	0.000	0.000
AS94_01685	hypothetical protein	0.939	0.000	0.000
AS94_01690	hypothetical protein	1.112	0.000	0.000
AS94_01695	cell cycle protein GpsB	1.240	0.000	0.000
AS94_01705	RNA methyltransferase	0.776	0.000	0.000
AS94_01710	hypothetical protein	0.838	0.001	0.002
AS94_01715	sulfite reductase subunit alpha	1.736	0.000	0.000
AS94_01720	dynamin family protein	0.862	0.000	0.000
AS94_01785	thymidylate synthase	0.640	0.000	0.000
AS94_01830	UDP-diphospho-muramoylpentapeptide beta-N-acetylglucosaminyltransferase	0.716	0.000	0.000
AS94_01835	phosphatidic acid phosphatase	0.712	0.000	0.000
AS94_01855	2-oxoglutarate dehydrogenase E1	0.749	0.000	0.000
AS94_01860	dihydrolipoamide succinyltransferase	0.503	0.000	0.000
AS94_01890	hypothetical protein	0.510	0.005	0.012
AS94_01895	nitric oxide reductase activation protein NorD	0.440	0.001	0.002
AS94_01905	tellurite resistance protein TelA	0.632	0.000	0.000
AS94_01910	5-bromo-4-chloroindolyl phosphate hydrolysis protein	0.643	0.000	0.001
AS94_01915	acylphosphatase	0.711	0.000	0.001
AS94_01965	dihydrodipicolinate synthase	0.722	0.008	0.018
AS94_01970	aspartate-semialdehyde dehydrogenase	0.788	0.002	0.006
AS94_01975	aspartate kinase	1.040	0.000	0.001
AS94_01990	thioredoxine reductase	1.610	0.000	0.000
AS94_02120	LytR family transcriptional regulator	0.923	0.000	0.000
AS94_02130	phosphatidylglycerol lysyltransferase	0.491	0.000	0.000

AS94_02155	DNA topoisomerase IV subunit A	0.352	0.002	0.005
AS94_02160	DNA topoisomerase IV subunit B	0.552	0.000	0.000
AS94_02175	4-hydroxybenzoyl-CoA thioesterase	0.518	0.000	0.000
AS94_02180	aconitate hydratase	0.738	0.000	0.000
AS94_02185	choline transporter	0.664	0.000	0.000
AS94_02240	secretion protein	0.883	0.000	0.000
AS94_02245	guanosine 5'-monophosphate oxidoreductase	1.455	0.000	0.000
AS94_02275	HAD family hydrolase	0.367	0.004	0.009
AS94_02280	homoserine kinase	0.931	0.000	0.000
AS94_02285	threonine synthase	0.589	0.000	0.000
AS94_02290	homoserine dehydrogenase	1.028	0.000	0.000
AS94_02295	aspartate kinase	1.786	0.000	0.000
AS94_02300	hypothetical protein	0.735	0.000	0.001
AS94_02480	DNA-binding protein	0.311	0.018	0.038
AS94_02545	glycerol kinase	0.527	0.000	0.000
AS94_02560	DNA mismatch repair protein MutL	0.631	0.000	0.000
AS94_02565	DNA mismatch repair protein MutS	0.494	0.000	0.000
AS94_02635	hypothetical protein	0.424	0.002	0.005
AS94_02645	zinc protease	0.371	0.016	0.036
AS94_02675	30S ribosomal protein S15	0.308	0.004	0.009
AS94_02685	tRNA pseudouridine synthase B	0.497	0.006	0.015
AS94_02700	50S ribosomal protein L7	0.624	0.000	0.001
AS94_02710	transcription elongation factor NusA	0.691	0.000	0.000
AS94_02715	ribosome maturation protein RimP	0.884	0.000	0.000
AS94_02725	prolyl-tRNA synthetase	0.509	0.000	0.000
AS94_02730	zinc metalloprotease	0.662	0.000	0.000
AS94_02735	phosphatidate cytidylyltransferase	0.535	0.000	0.001
AS94_02740	UDP pyrophosphate synthase	0.891	0.000	0.000
AS94_02755	elongation factor Ts	1.115	0.000	0.000
AS94_02760	30S ribosomal protein S2	1.029	0.000	0.000
AS94_02765	transcriptional regulator	0.678	0.000	0.000
AS94_02770	ATP-dependent protease	1.064	0.000	0.000
AS94_02775	ATP-dependent protease	1.054	0.000	0.000
AS94_02780	tyrosine recombinase XerC	1.247	0.000	0.000

AS94_02785	tRNA (uracil-5-)-methyltransferase	0.500	0.000	0.000
AS94_02790	DNA topoisomerase I	0.542	0.000	0.000
AS94_02815	succinyl-CoA synthetase subsunit alpha	0.905	0.000	0.000
AS94_02820	malate--CoA ligase subunit beta	1.209	0.000	0.000
AS94_02845	50S ribosomal protein L19	0.613	0.000	0.000
AS94_02880	chromosome segregation protein SMC	0.423	0.000	0.001
AS94_02890	acyl carrier protein	0.341	0.018	0.038
AS94_02905	phosphate acyltransferase	0.597	0.000	0.000
AS94_02910	transcription factor	0.953	0.000	0.000
AS94_02920	hypothetical protein	0.695	0.000	0.000
AS94_02925	hypothetical protein	0.375	0.008	0.019
AS94_02945	GTPase	0.476	0.002	0.004
AS94_03015	3-demethylubiquinone-9 3-methyltransferase	0.586	0.000	0.000
AS94_03020	hypothetical protein	1.416	0.007	0.016
AS94_03025	orotate phosphoribosyltransferase	1.077	0.000	0.000
AS94_03030	orotidine 5'-phosphate decarboxylase	1.367	0.000	0.000
AS94_03035	carbamoyl phosphate synthase large subunit	1.053	0.000	0.000
AS94_03040	carbamoyl phosphate synthase small subunit	0.581	0.006	0.014
AS94_03115	cell division protein FtsZ	0.604	0.000	0.000
AS94_03120	cell division protein FtsA	0.822	0.000	0.000
AS94_03125	cell division protein FtsQ	0.928	0.000	0.000
AS94_03130	UDP-N-acetylmuramoyl-L-alanyl-D-glutamate synthetase	0.934	0.000	0.000
AS94_03135	phospho-N-acetylmuramoyl-pentapeptide- transferase	0.826	0.000	0.000
AS94_03140	penicillin-binding protein 1	1.170	0.000	0.000
AS94_03145	cell division protein FtsL	0.888	0.000	0.000
AS94_03150	16S rRNA methyltransferase	1.212	0.000	0.000
AS94_03155	cell division protein MraZ	1.280	0.000	0.000
AS94_03165	hypothetical protein	1.215	0.000	0.000
AS94_03175	hypothetical protein	1.307	0.003	0.007
AS94_03180	hypothetical protein	1.479	0.002	0.005
AS94_03195	ethanolamine utilization protein EutQ	1.162	0.000	0.000
AS94_03245	leukocidin/Hemolysin toxin family protein	3.250	0.000	0.000
AS94_03310	succinate dehydrogenase flavoprotein subunit	0.464	0.001	0.002

AS94_03315	succinate dehydrogenase cytochrome B558	0.894	0.000	0.000
AS94_03330	DNA mismatch repair protein MutS	0.697	0.000	0.000
AS94_03335	DNA polymerase	0.666	0.000	0.000
AS94_03460	membrane protein	0.796	0.000	0.000
AS94_03465	protoheme IX farnesyltransferase	0.918	0.000	0.000
AS94_03470	heme A synthase	1.041	0.000	0.000
AS94_03475	pyruvate carboxylase	0.539	0.000	0.000
AS94_03490	hypothetical protein	0.349	0.010	0.024
AS94_03495	GTP-binding protein	0.529	0.000	0.000
AS94_03530	spermidine/putrescine ABC transporter substrate-binding protein	0.755	0.000	0.000
AS94_03570	2-oxoisovalerate dehydrogenase subunit beta	0.273	0.008	0.020
AS94_03575	pyruvate dehydrogenase E1 subunit alpha	0.329	0.001	0.004
AS94_03600	potassium transporter Trk	0.799	0.000	0.000
AS94_03605	cytochrome D ubiquinol oxidase subunit II	2.006	0.000	0.000
AS94_03610	cytochrome D ubiquinol oxidase subunit I	2.035	0.000	0.000
AS94_03620	phosphoenolpyruvate-protein phosphotransferase	0.676	0.000	0.000
AS94_03625	phosphocarrier protein HPr	0.818	0.000	0.000
AS94_03630	hypothetical protein	0.659	0.000	0.000
AS94_03640	membrane protein	0.657	0.000	0.000
AS94_03645	hypothetical protein	1.397	0.021	0.045
AS94_03665	phosphoribosylamine--glycine ligase	0.727	0.000	0.000
AS94_03670	purine biosynthesis protein purH	1.399	0.000	0.000
AS94_03675	phosphoribosylglycinamide formyltransferase	1.815	0.000	0.000
AS94_03680	phosphoribosylaminoimidazole synthetase	1.666	0.000	0.000
AS94_03685	amidophosphoribosyltransferase	1.530	0.000	0.000
AS94_03690	phosphoribosylformylglycinamidine synthase	1.235	0.000	0.000
AS94_03695	phosphoribosylformylglycinamidine synthase	0.766	0.007	0.016
AS94_03710	phosphoribosylaminoimidazole carboxylase	0.802	0.002	0.006
AS94_03720	tetrahydrofolate dehydrogenase	0.820	0.000	0.000
AS94_03730	chitinase	1.288	0.000	0.000
AS94_03755	methicillin resistance protein FmtA	0.938	0.000	0.000
AS94_03885	CAAX amino terminal protease	0.522	0.004	0.009
AS94_03965	hypothetical protein	0.913	0.000	0.000

AS94_04055	tryptophanyl-tRNA synthetase	0.309	0.018	0.039
AS94_04085	peptide ABC transporter substrate-binding protein	0.857	0.000	0.000
AS94_04090	peptide ABC transporter ATP-binding protein	0.781	0.000	0.001
AS94_04095	peptide ABC transporter ATP-binding protein	0.841	0.000	0.001
AS94_04100	peptide ABC transporter permease	0.798	0.000	0.000
AS94_04105	peptide ABC transporter permease	0.892	0.000	0.000
AS94_04115	3-oxoacyl-ACP synthase	0.295	0.012	0.027
AS94_04120	3-oxoacyl-ACP synthase	0.517	0.001	0.002
AS94_04135	phosphatidylethanolamine-binding protein	0.681	0.008	0.018
AS94_04145	ATP-dependent Clp protease ATP-binding protein	0.588	0.000	0.000
AS94_04160	phosphatase	0.388	0.009	0.020
AS94_04170	hypothetical protein	0.505	0.005	0.013
AS94_04190	signal peptidase IB	1.066	0.000	0.000
AS94_04195	signal peptidase I	1.031	0.000	0.000
AS94_04200	hypothetical protein	0.433	0.007	0.016
AS94_04220	argininosuccinate synthase	6.152	0.000	0.000
AS94_04225	argininosuccinate lyase	5.631	0.000	0.000
AS94_04235	glutamate dehydrogenase	0.255	0.023	0.049
AS94_04240	ornithine-oxoacid aminotransferase	0.680	0.000	0.000
AS94_04250	general stress protein	0.361	0.012	0.027
AS94_04320	NADH dehydrogenase	0.734	0.000	0.000
AS94_04335	NADH dehydrogenase	0.362	0.005	0.013
AS94_04340	disulfide oxidoreductase	0.723	0.000	0.001
AS94_04350	D-alanyl-lipoteichoic acid biosynthesis protein DltD	0.752	0.000	0.000
AS94_04360	D-alanyl transfer protein DltB	0.718	0.000	0.000
AS94_04365	D-alanine--poly(phosphoribitol) ligase	0.968	0.000	0.000
AS94_04405	5'-nucleotidase	0.507	0.000	0.001
AS94_04410	membrane protein	0.513	0.006	0.015
AS94_04450	Fe-S cluster assembly protein SufD	0.535	0.000	0.000
AS94_04455	iron ABC transporter ATP-binding protein	0.820	0.000	0.000
AS94_04460	hypothetical protein	0.824	0.000	0.000
AS94_04470	methionine ABC transporter substrate-binding protein	0.403	0.015	0.032
AS94_04475	ABC transporter permease	0.565	0.017	0.037
AS94_04485	thioredoxin	0.494	0.023	0.049

AS94_04490	topiosmerase	0.653	0.002	0.004
AS94_04540	phosphoglycerate mutase	0.940	0.000	0.000
AS94_04550	hypothetical protein	0.812	0.000	0.001
AS94_04605	clumping factor A	0.694	0.000	0.000
AS94_04635	enterotoxin	0.775	0.000	0.000
AS94_04655	hypothetical protein	0.752	0.000	0.000
AS94_04670	hydrolase	1.770	0.000	0.000
AS94_04725	haloacid dehalogenase	1.456	0.000	0.000
AS94_04730	glycine/betaine MFS transporter	1.381	0.000	0.000
AS94_04735	long-chain fatty acid--CoA ligase	3.781	0.000	0.000
AS94_04740	acetyl-CoA acetyltransferase	2.229	0.000	0.000
AS94_04745	vraC	3.127	0.000	0.000
AS94_04750	hypothetical protein	3.366	0.000	0.000
AS94_04755	vraX	3.312	0.000	0.000
AS94_04780	amino acid permease	0.782	0.000	0.000
AS94_04810	mevalonate kinase	1.382	0.000	0.000
AS94_04815	diphosphomevalonate decarboxylase	1.502	0.000	0.000
AS94_04820	phosphomevalonate kinase	1.298	0.000	0.000
AS94_04840	transposase	0.745	0.000	0.000
AS94_04845	hypothetical protein	0.905	0.007	0.017
AS94_04865	oxidoreductase ion channel protein IolS	0.429	0.003	0.008
AS94_04950	lysyl-tRNA synthetase	0.428	0.000	0.000
AS94_04955	2-amino-4-hydroxy-6- hydroxymethyldihydropteridine pyrophosphokinase	1.123	0.000	0.000
AS94_04960	dihydroneopterin aldolase	1.843	0.000	0.000
AS94_04965	dihydropteroate synthase	2.032	0.000	0.000
AS94_04980	zinc metalloprotease	0.368	0.000	0.001
AS94_04985	hypoxanthine phosphoribosyltransferase	0.367	0.009	0.022
AS94_05140	hypothetical protein	0.541	0.020	0.043
AS94_05190	1-phosphatidylinositol phosphodiesterase	0.916	0.000	0.001
AS94_05340	replication initiation factor family protein	3.139	0.004	0.010
AS94_05345	hypothetical protein	3.206	0.008	0.019
AS94_05350	hypothetical protein	3.347	0.007	0.016
AS94_05355	hypothetical protein	3.487	0.006	0.014

AS94_05420		ATP-binding protein	0.456	0.001	0.003
AS94_05435		integrase	1.211	0.008	0.018
AS94_05465		hemolysin III	0.763	0.000	0.000
AS94_05470		uridylyltransferase	0.742	0.000	0.000
AS94_05475		membrane protein	0.671	0.000	0.000
AS94_05480		hypothetical protein	0.698	0.000	0.000
AS94_05485		membrane protein	2.482	0.000	0.000
AS94_05525	*asp23*	alkaline shock protein 23	1.013	0.000	0.000
AS94_05530		membrane protein	0.845	0.000	0.000
AS94_05535		hypothetical protein	0.691	0.000	0.000
AS94_05540		glycine/betaine ABC transporter permease	1.196	0.000	0.000
AS94_05625		hyaluronate lyase	0.558	0.002	0.004
AS94_05650		toxin	1.240	0.000	0.000
AS94_05680		30S ribosomal protein S9	0.961	0.000	0.000
AS94_05685		50S ribosomal protein L13	1.063	0.000	0.000
AS94_05775		30S ribosomal protein S8	0.479	0.001	0.003
AS94_05785		50S ribosomal protein L5	0.452	0.001	0.002
AS94_05790		50S ribosomal protein L24	0.643	0.000	0.000
AS94_05795		50S ribosomal protein L14	0.359	0.008	0.019
AS94_05800		30S ribosomal protein S17	0.698	0.000	0.001
AS94_05810		50S ribosomal protein L16	0.540	0.000	0.000
AS94_05815		30S ribosomal protein S3	0.601	0.000	0.000
AS94_05820		50S ribosomal protein L22	0.524	0.000	0.001
AS94_05825		30S ribosomal protein S19	0.607	0.000	0.000
AS94_05830		50S ribosomal protein L2	0.452	0.000	0.001
AS94_05835		50S ribosomal protein L23	0.627	0.000	0.001
AS94_05840		50S ribosomal protein L4	0.377	0.004	0.010
AS94_05845		50S ribosomal protein L3	0.609	0.000	0.000
AS94_05850		30S ribosomal protein S10	0.723	0.000	0.000
AS94_06080		hypothetical protein	0.859	0.000	0.000
AS94_06085		sodium:proton antiporter	0.517	0.000	0.001
AS94_06145		LytR family transcriptional regulator	0.788	0.000	0.000
AS94_06170		RpiR family transcriptional regulator	0.335	0.011	0.025
AS94_06205		hypothetical protein	1.951	0.000	0.000

AS94_06245	imidazolonepropionase	2.024	0.000	0.000
AS94_06250	urocanate hydratase	2.078	0.000	0.000
AS94_06300	hypothetical protein	1.044	0.000	0.000
AS94_06310	sodium:glutamate symporter	0.613	0.000	0.000
AS94_06325	3-hydroxyacyl-CoA dehydrogenase	0.582	0.010	0.023
AS94_06365	membrane protein	1.731	0.000	0.000
AS94_06425	malate:quinone oxidoreductase	0.872	0.000	0.000
AS94_06430	L-lactate permease	0.405	0.002	0.005
AS94_06435	CDP-glycerol glycerophosphotransferase	0.337	0.015	0.033
AS94_06450	quinone oxidoreductase	0.365	0.010	0.023
AS94_06510	hypothetical protein	0.855	0.006	0.013
AS94_06655	amino acid ABC transporter substrate-binding protein	0.523	0.000	0.001
AS94_06670	phosphoglyceromutase	0.821	0.000	0.000
AS94_06680	hypothetical protein	1.886	0.000	0.000
AS94_06685	gamma-hemolysin subunit A	0.956	0.000	0.000
AS94_06755	glycerate kinase	1.762	0.000	0.000
AS94_06760	membrane protein	1.999	0.000	0.000
AS94_06770	transcriptional regulator	1.084	0.001	0.002
AS94_06855	chloramphenicol resistance protein DHA1	0.578	0.002	0.006
AS94_06940	short-chain dehydrogenase	0.425	0.001	0.003
AS94_06945	aminobenzoyl-glutamate transporter	1.048	0.000	0.000
AS94_07095	membrane protein	0.345	0.015	0.032
AS94_07100	glucarate transporter	0.578	0.000	0.001
AS94_07190	serine dehydratase subunit alpha	1.378	0.000	0.000
AS94_07195	serine dehydratase	1.527	0.000	0.000
AS94_07200	transcriptional regulator	1.288	0.000	0.000
AS94_07270	hydroxymethylglutaryl-CoA synthase	1.378	0.000	0.000
AS94_07280	Clp protease ATP-binding protein	1.294	0.000	0.000
AS94_07310	1-pyrroline-5-carboxylate dehydrogenase	1.261	0.000	0.000
AS94_07320	hypothetical protein	4.145	0.000	0.000
AS94_07350	dehydrosqualene synthase	0.874	0.000	0.000
AS94_07355	4_4'-diaponeurosporenoate glycosyltransferase	0.602	0.001	0.002
AS94_07360	diapolycopene oxygenase	0.589	0.000	0.001
AS94_07480	dihydroorotate dehydrogenase	0.991	0.000	0.000

AS94_07540		amino acid permease	1.069	0.000	0.000
AS94_07545		4-aminobutyrate aminotransferase	1.378	0.000	0.000
AS94_07615		citrate transporter	0.791	0.006	0.015
AS94_07745		Replication and maintenance protein	14.474	0.000	0.000
AS94_07750		SAM-dependent methlyltransferase	15.654	0.000	0.000
AS94_07835	*sarA*	transcriptional regulator	0.621	0.000	0.000
AS94_07855		recombinase	0.542	0.015	0.033
AS94_07860		cation:proton antiporter	0.930	0.000	0.000
AS94_07865		cation:proton antiporter	1.002	0.001	0.003
AS94_07870		cation:proton antiporter	1.711	0.000	0.000
AS94_07875		cation:proton antiporter	1.466	0.000	0.000
AS94_07880		cation:proton antiporter	1.602	0.000	0.000
AS94_07890		cation:proton antiporter	1.534	0.000	0.000
AS94_07900		manganese ABC transporter substrate-binding protein	1.328	0.000	0.000
AS94_07905		membrane protein	1.233	0.000	0.000
AS94_07910		phosphonate ABC transporter ATP-binding protein	1.616	0.000	0.000
AS94_07920		membrane protein	1.875	0.000	0.000
AS94_08150		hypothetical protein	0.884	0.000	0.000
AS94_08155		UDP pyrophosphate phosphatase	0.818	0.000	0.000
AS94_08160		cysteine ABC transporter ATP-binding protein	0.456	0.000	0.000
AS94_08165		cysteine ABC transporter ATP-binding protein	0.633	0.000	0.000
AS94_08175		cobalamin biosynthesis protein CobW	0.369	0.013	0.029
AS94_08190		membrane protein	0.854	0.000	0.000
AS94_08205		hypothetical protein	0.684	0.010	0.022
AS94_08215		multidrug MFS transporter	0.549	0.000	0.000
AS94_08255		glyoxal reductase	0.603	0.000	0.000
AS94_08330		glycerol phosphate lipoteichoic acid synthase	1.274	0.000	0.000
AS94_08350		ABC transporter permease	0.345	0.006	0.015
AS94_08375		peptide ABC transporter permease	0.447	0.000	0.001
AS94_08470		membrane protein	0.675	0.000	0.000
AS94_08480		hypothetical protein	0.888	0.000	0.000
AS94_08575		hypothetical protein	0.777	0.000	0.000
AS94_08580		sporulation protein	0.537	0.000	0.000
AS94_08590		ATP-dependent Clp protease proteolytic subunit	0.617	0.000	0.000

AS94_08610	hypothetical protein	0.436	0.000	0.001
AS94_08615	transcriptional regulator	0.515	0.000	0.000
AS94_08675	integrase	0.555	0.000	0.001
AS94_08680	DNA-binding protein	0.540	0.013	0.029
AS94_08685	hypothetical protein	1.686	0.014	0.030
AS94_08765	transposase	0.728	0.003	0.007
AS94_08810	NA	1.507	0.000	0.000
AS94_08960	phospholipase D	0.564	0.000	0.000
AS94_08965	phosphohydrolase	0.516	0.001	0.002
AS94_08970	membrane protein	0.290	0.015	0.033
AS94_09015	UDP-N-acetylglucosamine 1-carboxyvinyltransferase	0.379	0.001	0.004
AS94_09020	membrane protein	0.970	0.023	0.049
AS94_09040	F0F1 ATP synthase subunit alpha	0.654	0.000	0.000
AS94_09045	F0F1 ATP synthase subunit delta	0.627	0.000	0.000
AS94_09050	F0F1 ATP synthase subunit B	0.895	0.000	0.000
AS94_09055	F0F1 ATP synthase subunit C	0.383	0.005	0.013
AS94_09060	F0F1 ATP synthase subunit A	0.465	0.000	0.000
AS94_09065	ATP synthase	0.592	0.000	0.000
AS94_09070	UDP-N-acetylglucosamine 2-epimerase	0.854	0.000	0.000
AS94_09075	uracil phosphoribosyltransferase	0.793	0.000	0.000
AS94_09080	serine hydroxymethyltransferase	0.792	0.000	0.000
AS94_09085	hypothetical protein	0.872	0.000	0.000
AS94_09100	N5-glutamine S-adenosyl-L-methionine-dependent methyltransferase	0.541	0.000	0.000
AS94_09105	peptide chain release factor 1	0.557	0.000	0.000
AS94_09115	50S ribosomal protein L31	0.289	0.020	0.044
AS94_09150	CTP synthetase	0.987	0.000	0.000
AS94_09160	acetyltransferase	0.884	0.000	0.000
AS94_09165	pantothenate kinase	0.645	0.001	0.003
AS94_09240	ArsR family transcriptional regulator	1.904	0.000	0.000
AS94_09245	cation transporter	1.318	0.000	0.000
AS94_09250	lytic regulatory protein	0.743	0.000	0.000
AS94_09275	PTS mannitol transporter subunit IIB	1.842	0.000	0.000
AS94_09280	PTS lactose transporter subunit IIB	1.918	0.000	0.000

AS94_09285	PTS mannitol transporter subunit IIA	1.500	0.000	0.000
AS94_09290	mannitol-1-phosphate 5-dehydrogenase	1.509	0.000	0.000
AS94_09310	ABC transporter permease	0.367	0.002	0.006
AS94_09315	arginase	0.880	0.000	0.000
AS94_09360	NA	0.611	0.018	0.039
AS94_09440	membrane protein	1.102	0.007	0.017
AS94_09450	membrane protein	0.844	0.004	0.009
AS94_09490	surface protein	0.634	0.000	0.000
AS94_09505	antibiotic MFS transporter	0.562	0.002	0.006
AS94_09560	capsular polysaccharide biosynthesis protein CapA	1.641	0.000	0.000
AS94_09565	capsular polysaccharide biosynthesis protein Cap5B	1.808	0.000	0.000
AS94_09570	capsular polysaccharide biosynthesis protein Cap8C	1.647	0.000	0.000
AS94_09575	polysaccharide biosynthesis protein EpsC	1.611	0.000	0.000
AS94_09580	UDP-glucose 4-epimerase	1.840	0.000	0.000
AS94_09585	capsular polysaccharide biosynthesis protein Cap8F	1.717	0.000	0.000
AS94_09590	UDP-N-acetylglucosamine 2-epimerase	1.877	0.000	0.000
AS94_09595	capsular polysaccharide biosynthesis protein	1.991	0.000	0.000
AS94_09600	capsular polysaccharide biosynthesis protein	1.620	0.000	0.000
AS94_09605	capsular polysaccharide biosynthesis protein	1.536	0.000	0.000
AS94_09610	capsular polysaccharide biosynthesis protein	1.070	0.000	0.000
AS94_09615	glycosyltransferase family 1	0.774	0.000	0.000
AS94_09620	capsular polysaccharide biosynthesis protein Cap8M	0.691	0.001	0.004
AS94_09625	UDP-glucose 4-epimerase	0.622	0.001	0.002
AS94_09630	UDP-N-acetyl-D-mannosamine dehydrogenase	0.571	0.000	0.000
AS94_09660	hypothetical protein	1.430	0.002	0.004
AS94_09690	multidrug MFS transporter	1.144	0.000	0.000
AS94_09695	peptide synthetase	0.353	0.002	0.004
AS94_09755	cellobiose operon outer surface protein	1.293	0.000	0.000
AS94_09760	N-acetylmuramic acid-6-phosphate etherase	1.205	0.000	0.000
AS94_09765	permease	1.432	0.000	0.000
AS94_09770	RpiR family transcriptional regulator	1.547	0.000	0.000
AS94_09820	peptidase M23	0.956	0.000	0.000
AS94_09865	antiporter	2.802	0.000	0.000
AS94_09880	iron ABC transporter substrate-binding protein	0.571	0.015	0.033

AS94_09920	3-hydroxyacyl-CoA dehydrogenase	1.118	0.000	0.000
AS94_09925	glutaryl-CoA dehydrogenase	1.385	0.000	0.001
AS94_09930	long-chain fatty acid--CoA ligase	1.974	0.000	0.000
AS94_09935	coenzyme A transferase	2.050	0.000	0.000
AS94_09955	NmrA family protein	2.065	0.000	0.000
AS94_09960	DeoR family transcriptional regulator	1.475	0.000	0.000
AS94_09985	lactate dehydrogenase	1.252	0.000	0.000
AS94_09995	inosine-uridine preferring nucleoside hydrolase	0.524	0.008	0.018
AS94_10035	2-C-methyl-D-erythritol 4-phosphate cytidylyltransferase	0.961	0.000	0.000
AS94_10040	ribitol-5-phosphate dehydrogenase	0.720	0.001	0.003
AS94_10045	teichoic acid biosynthesis protein	0.961	0.000	0.000
AS94_10050	CDP-glycerol:glycerophosphate glycerophosphotransferase	0.422	0.016	0.034
AS94_10095	antiholin	0.788	0.000	0.000
AS94_10120	hypothetical protein	0.817	0.000	0.001
AS94_10125	ribokinase	3.000	0.000	0.000
AS94_10130	ribose pyranase	3.101	0.000	0.000
AS94_10135	ribose transporter RbsU	3.014	0.000	0.000
AS94_10200	virulence factor EsxA	0.490	0.000	0.000
AS94_10205	type VII secretion protein EsaA	0.886	0.000	0.000
AS94_10220	type VII secretion protein EssB	0.904	0.001	0.002
AS94_10225	cell division protein FtsK	0.375	0.024	0.049
AS94_10260	membrane protein	0.767	0.013	0.029
AS94_10315	hypothetical protein	0.863	0.000	0.000
AS94_10325	branched-chain amino acid transporter II carrierprotein	0.668	0.000	0.000
AS94_10390	histidine transporter	2.250	0.000	0.000
AS94_10395	lipase	0.939	0.000	0.000
AS94_10470	sn-glycerol-3-phosphate transporter	1.983	0.000	0.000
AS94_10555	acetyl-CoA acetyltransferase	0.778	0.000	0.000
AS94_10565	5-methyltetrahydropteroyltriglutamate-- homocysteine methyltransferase	0.777	0.000	0.000
AS94_10570	5_10-methylenetetrahydrofolate reductase	0.913	0.000	0.000
AS94_10575	cystathionine beta-lyase	1.129	0.001	0.003

AS94_10580	cystathionine gamma-synthase	1.239	0.000	0.001
AS94_10600	GTP-binding protein YchF	0.486	0.000	0.001
AS94_10660	peptidase	1.600	0.000	0.000
AS94_10670	membrane protein	0.649	0.000	0.000
AS94_10725	hypothetical protein	0.638	0.000	0.000
AS94_10760	hypothetical protein	0.432	0.002	0.006
AS94_10785	growth inhibitor PemK	0.716	0.000	0.000
AS94_10790	transposase	0.770	0.009	0.021
AS94_10800	hypothetical protein	1.022	0.000	0.000
AS94_10805	hypothetical protein	0.657	0.000	0.000
AS94_10910	hypothetical protein	0.681	0.001	0.002
AS94_10915	membrane protein	0.506	0.002	0.006
AS94_10930	membrane protein	0.803	0.003	0.007
AS94_10935	cobalamin synthesis protein CobW	2.349	0.000	0.000
AS94_10985	methionine ABC transporter ATP-binding protein	1.379	0.000	0.000
AS94_11005	membrane protein	0.379	0.021	0.044
AS94_11055	oligo-1_6-glucosidase	0.290	0.013	0.029
AS94_11060	GntR family transctiptional regulator	0.369	0.002	0.006
AS94_11070	acetyltransferase	0.671	0.005	0.011
AS94_11170	16S rRNA methyltransferase	0.654	0.000	0.000
AS94_11175	tRNA uridine 5-carboxymethylaminomethyl modification protein	0.622	0.000	0.000
AS94_11180	tRNA modification GTPase	0.465	0.001	0.002
AS94_11185	ribonuclease P	0.738	0.003	0.008
AS94_11190	50S ribosomal protein L34	0.691	0.000	0.000
AS94_11195	chromosome replication protein DnaA	0.676	0.000	0.000
AS94_11200	DNA polymerase III subunit beta	0.677	0.000	0.000
AS94_11210	recombinase F	0.737	0.000	0.000
AS94_11215	DNA gyrase subunit B	0.798	0.000	0.000
AS94_11220	DNA topoisomerase IV subunit A	0.757	0.000	0.000
AS94_11230	histidine ammonia-lyase	0.796	0.000	0.000
AS94_11235	seryl-tRNA synthetase	1.149	0.000	0.000
AS94_11275	adenylosuccinate synthetase	2.120	0.000	0.000
AS94_11320	50S rRNA methyltransferase	0.816	0.000	0.000

AS94_11325	hypothetical protein	0.393	0.001	0.003
AS94_11355	penicillin-binding protein	0.836	0.000	0.000
AS94_11360	methicillin resistance protein	0.420	0.005	0.013
AS94_11430	hydrolase	2.577	0.000	0.000
AS94_11470	UDP-glucose 4-epimerase	1.274	0.000	0.000
AS94_11485	2-amino-3-ketobutyrate CoA ligase	0.753	0.000	0.000
AS94_11530	16S rRNA methyltransferase	0.752	0.000	0.000
AS94_11535	50S ribosomal protein L7/L12	0.922	0.000	0.000
AS94_11540	50S ribosomal protein L10	1.025	0.000	0.000
AS94_11545	50S ribosomal protein L1	0.948	0.000	0.000
AS94_11550	50S ribosomal protein L11	0.572	0.000	0.000
AS94_11575	hypothetical protein	0.637	0.000	0.001
AS94_11580	RNA methyltransferase	0.358	0.022	0.047
AS94_11610	DNA repair protein RadA	0.344	0.007	0.016
AS94_11615	ATP-dependent Clp protease ATP-binding protein	0.477	0.000	0.000
AS94_11620	ATP:guanido phosphotransferase	0.481	0.000	0.000
AS94_11625	excinuclease ABC subunit B	0.498	0.000	0.000
AS94_11630	CtsR family transcriptional regulator	0.406	0.000	0.001
AS94_11665	DNA damage-inducible protein DinB	0.399	0.021	0.045
AS94_11680	lactonase	1.005	0.000	0.000
AS94_11740	lipase	1.830	0.000	0.000
AS94_11765	transcriptional regulator	0.620	0.000	0.000
AS94_11815	hypothetical protein	0.719	0.003	0.007
AS94_11825	flavin reductase	0.948	0.000	0.000
AS94_11855	preprotein translocase subunit SecA	0.381	0.003	0.007
AS94_11870	surface anchored protein	1.032	0.000	0.000
AS94_11875	isochorismatase hydrolase	0.363	0.003	0.007
AS94_11880	N-acetylmuramoyl-L-alanine amidase	0.606	0.000	0.000
AS94_11940	carbamate kinase	0.738	0.000	0.000
AS94_11945	Crp/Fnr family transcriptional regulator	0.736	0.000	0.000
AS94_11985	multidrug ABC transporter ATP-binding protein	0.508	0.000	0.000
AS94_11990	hypothetical protein	0.619	0.000	0.000
AS94_12020	RecX family transcriptional regulator	0.815	0.000	0.000
AS94_12025	glycosyltransferase	2.930	0.000	0.000

AS94_12030	general stress protein	0.329	0.002	0.006
AS94_12410	ribonuclease BN	0.808	0.000	0.000
AS94_12415	LuxR family transcriptional regulator	3.056	0.000	0.000
AS94_12420	sensor histidine kinase	3.494	0.000	0.000
AS94_12425	transporter	3.384	0.000	0.000
AS94_12430	hypothetical protein	3.562	0.000	0.000
AS94_12465	UDP-N-acetylmuramate--alanine ligase	0.425	0.001	0.004
AS94_12490	RNA methyltransferase	0.577	0.000	0.000
AS94_12545	adenylosuccinate lyase	0.732	0.000	0.000
AS94_12550	cysteine protease	2.179	0.000	0.000
AS94_12555	staphostatin A	1.670	0.000	0.000
AS94_12590	C4-dicarboxylate ABC transporter	0.585	0.000	0.000
AS94_12630	beta-lactamase	0.535	0.004	0.009
AS94_12635	beta-lactamase	0.552	0.000	0.000
AS94_12645	hypothetical protein	0.583	0.000	0.000
AS94_12655	transposase Tn554	0.299	0.015	0.033
AS94_12690	membrane protein	0.448	0.015	0.033
AS94_12700	membrane protein	1.133	0.000	0.000
AS94_12705	antibiotic ABC transporter ATP-binding protein	0.870	0.000	0.000
AS94_12710	membrane protein	1.031	0.006	0.015
AS94_12715	sodium ABC transporter ATP-binding protein	1.381	0.000	0.000
AS94_12720	GntR family transcriptional regulator	1.258	0.000	0.000
AS94_12740	membrane protein	0.367	0.016	0.035
AS94_12755	complement inhibitor	1.320	0.000	0.000
AS94_12760	histidine kinase	1.086	0.007	0.016
AS94_12765	peptidoglycan hydrolase	0.537	0.001	0.004
AS94_12770	staphylokinase	0.499	0.024	0.050
AS94_12845	molecular chaperone GroEL	0.417	0.001	0.004
AS94_12850	molecular chaperone GroES	0.925	0.000	0.000
AS94_12970	dihydroxy-acid dehydratase	1.727	0.000	0.000
AS94_12975	acetolactate synthase	1.070	0.000	0.000
AS94_12985	ketol-acid reductoisomerase	1.081	0.021	0.044
AS94_13490	peptidyl-prolyl cis-trans isomerase	2.758	0.000	0.000
AS94_13500	DNA double-strand break repair Rad50 ATPase	0.415	0.000	0.000

AS94_13505		DNA repair exonuclease	0.451	0.000	0.001
AS94_13540		fumarate hydratase	0.323	0.013	0.029
AS94_13575		amino acid ABC transporter ATP-binding protein	4.322	0.000	0.000
AS94_13580		glutamate ABC transporter permease	4.340	0.000	0.000
AS94_12040	ϕSA169	hypothetical protein	1.352	0.014	0.031
AS94_12055		autolysin	0.907	0.000	0.000
AS94_12070		tail protein	1.010	0.000	0.000
AS94_12075		cell wall hydrolase	1.057	0.000	0.000
AS94_12090		hypothetical protein	1.351	0.009	0.020
AS94_12095		hypothetical protein	0.901	0.000	0.000
AS94_12100		minor structural protein	0.806	0.000	0.000
AS94_12105		peptidase	1.095	0.000	0.000
AS94_12110		phage tail protein	1.205	0.000	0.000
AS94_12115		membrane protein	0.843	0.000	0.000
AS94_12120		phi 11	1.235	0.001	0.002
AS94_12125		hypothetical protein	1.259	0.000	0.000
AS94_12130		tail protein	1.063	0.000	0.000
AS94_12135		phi 11	1.024	0.003	0.006
AS94_12140		hypothetical protein	1.349	0.002	0.004
AS94_12145		hypothetical protein	1.717	0.001	0.002
AS94_12150		phage head-tail adapter protein	1.569	0.000	0.000
AS94_12155		phi 11	1.495	0.010	0.022
AS94_12160		hypothetical protein	1.100	0.000	0.000
AS94_12165		phage capsid protein	1.440	0.000	0.000
AS94_12175		phage head morphogenesis protein	1.017	0.000	0.000
AS94_12180		phage portal protein	0.948	0.000	0.000
AS94_12185		hypothetical protein	1.121	0.000	0.000
AS94_12190		terminase	0.929	0.000	0.000
AS94_12345		BRO-like protein	0.458	0.006	0.015
AS94_12375		integrase	0.534	0.021	0.045
AS94_13065	mutual prophage	hypothetical protein	0.973	0.022	0.047
AS94_13265		hypothetical protein	5.182	0.000	0.000
AS94_13280		hypothetical protein	1.551	0.015	0.033

AS94_13290	hypothetical protein	8.643	0.000	0.000
AS94_13305	DNA replication protein DnaC	1.570	0.000	0.000
AS94_13310	replication protein	10.530	0.000	0.000
AS94_13315	hypothetical protein	9.973	0.000	0.000
AS94_13395	integrase	1.071	0.000	0.000
