# Supplementary material for: Transcriptome Analyses of Prophage in Mediating Persistent Methicillin-Resistant Staphylococcus aureus Endovascular Infection
Source: Genes (Basel). 2022 Aug 25;13(9):1527. doi: 10.3390/genes13091527 (PMC9498598; doi:10.3390/genes13091527)
Supplement: Supplementary file 1 [file genes-13-01527-s001.zip › Table S6.pdf]

Table S6. Down-regulated DEGs in 300-169 vs. 301-188::ϕSA169

locus	gene	group	product	log_2(fold change)	*p* value	*p* adj
AS94_00045		host genes	membrane protein	-0.567	0.007	0.016
AS94_00055			dipeptidyl aminopeptidase	-0.623	0.000	0.000
AS94_00075			glyoxal reductase	-0.346	0.017	0.036
AS94_00110			membrane protein	-0.611	0.000	0.001
AS94_00195			multidrug MFS transporter	-0.461	0.001	0.002
AS94_00205			hypothetical protein	-0.894	0.000	0.000
AS94_00215			NAD(FAD)-utilizing dehydrogenase	-0.387	0.017	0.036
AS94_00245			phosphotransferase	-0.916	0.000	0.000
AS94_00250			tRNA (guanine-N(7)-)-methyltransferase	-0.563	0.000	0.000
AS94_00255			hypothetical protein	-0.895	0.000	0.000
AS94_00270			thioredoxin	-0.406	0.004	0.010
AS94_00305			3-deoxy-7-phosphoheptulonate synthase	-1.566	0.000	0.000
AS94_00345			tyrosyl-tRNA synthase	-0.433	0.001	0.003
AS94_00355			1-acyl-sn-glycerol-3-phosphate acyltransferase	-0.410	0.014	0.032
AS94_00370			3-phosphoglycerate dehydrogenase	-0.480	0.001	0.002
AS94_00395			hypothetical protein	-0.572	0.001	0.003
AS94_00435			universal stress protein UspA	-1.876	0.000	0.000
AS94_00445			dipeptidase	-0.632	0.000	0.000
AS94_00450			beta-lactamase	-1.457	0.000	0.000
AS94_00460			universal stress protein UspA	-1.219	0.000	0.000
AS94_00500			acetyl-CoA carboxylase subunit alpha	-0.284	0.014	0.032
AS94_00505			6-phosphofructokinase	-1.025	0.000	0.000
AS94_00510			pyruvate kinase	-0.834	0.000	0.000
AS94_00525			isocitrate dehydrogenase	-0.533	0.000	0.000
AS94_00545			DNA polymerase I	-0.455	0.000	0.000
AS94_00550			formamidopyrimidine-DNA glycosylase	-0.471	0.000	0.000
AS94_00555			dephospho-CoA kinase	-1.236	0.000	0.000
AS94_00575			primosomal protein DnaI	-0.388	0.001	0.002
AS94_00580			threonyl-tRNA synthase	-1.991	0.000	0.000
AS94_00585			gamma-aminobutyrate permease	-1.476	0.000	0.000
AS94_00645			uroporphyrinogen III synthase	-0.371	0.019	0.041

AS94_00650	delta-aminolevulinic acid dehydratase	-0.343	0.004	0.010
AS94_00655	glutamate-1-semialdehyde aminotransferase	-0.492	0.000	0.000
AS94_00665	DNA-3-methyladenine glycosylase	-1.350	0.000	0.000
AS94_00730	50S ribosomal protein L27	-1.359	0.000	0.000
AS94_00735	GTPase CgtA	-0.346	0.004	0.011
AS94_00770	preprotein translocase subunit SecD	-0.244	0.021	0.044
AS94_00785	GTP pyrophosphokinase	-0.241	0.022	0.047
AS94_00795	cell wall amidase	-0.446	0.001	0.002
AS94_00800	hypothetical protein	-0.383	0.011	0.024
AS94_00810	aspartyl-tRNA synthase	-0.611	0.000	0.000
AS94_00840	hypothetical protein	-0.828	0.000	0.000
AS94_00860	hypothetical protein	-0.396	0.001	0.002
AS94_00865	alanyl-tRNA synthase	-0.431	0.000	0.000
AS94_00870	hypothetical protein	-0.541	0.001	0.002
AS94_00875	Holliday junction resolvase	-0.446	0.000	0.001
AS94_00880	hypothetical protein	-0.433	0.001	0.003
AS94_00905	transcription elongation factor GreA	-0.305	0.017	0.036
AS94_01005	deoxycytidylate deaminase	-0.694	0.000	0.000
AS94_01185	superoxide dismutase	-0.779	0.000	0.000
AS94_01260	shikimate kinase	-0.493	0.003	0.008
AS94_01265	glycine cleavage system protein T	-0.518	0.000	0.001
AS94_01270	glycine dehydrogenase subunit 1	-0.706	0.000	0.000
AS94_01275	glycine dehydrogenase subunit 2	-1.029	0.000	0.000
AS94_01370	2-oxoglutarate dehydrogenase E2	-0.639	0.000	0.000
AS94_01405	transcriptional regulator	-0.834	0.000	0.000
AS94_01410	trehalose-6-phosphate hydrolase	-1.788	0.000	0.000
AS94_01430	pyrroline-5-carboxylate reductase	-0.677	0.000	0.000
AS94_01460	tyrosine recombinase XerD	-0.800	0.000	0.000
AS94_01490	sensor histidine kinase	-0.314	0.003	0.006
AS94_01540	asparaginase	-0.421	0.010	0.023
AS94_01560	glycerol-3-phosphate dehydrogenase	-0.621	0.000	0.000
AS94_01565	DNA-binding protein	-0.780	0.000	0.000
AS94_01630	hypothetical protein	-0.463	0.022	0.046
AS94_01665	endonuclease III	-0.649	0.000	0.001

AS94_01700	hypothetical protein	-0.461	0.000	0.000
AS94_01735	threonine dehydratase	-0.402	0.011	0.025
AS94_01745	quinolone resistance protein NorB	-0.495	0.001	0.004
AS94_01750	matrix-binding protein	-0.381	0.000	0.001
AS94_01780	hypothetical protein	-0.648	0.000	0.000
AS94_01795	hypothetical protein	-0.444	0.000	0.000
AS94_01800	methionine sulfoxide reductase A	-0.371	0.001	0.002
AS94_01805	methionine sulfoxide reductase B	-0.466	0.000	0.001
AS94_01810	PTS glucose transporter subunit IIA	-0.432	0.000	0.001
AS94_01920	hypothetical protein	-0.943	0.000	0.000
AS94_01935	protease	-0.909	0.000	0.000
AS94_02055	aminoacyltransferase	-0.301	0.010	0.023
AS94_02060	aminoacyltransferase	-0.300	0.022	0.046
AS94_02100	aminopeptidase	-0.875	0.000	0.000
AS94_02105	prephenate dehydrogenase	-0.810	0.000	0.000
AS94_02110	DNA repair protein MucB	-1.518	0.000	0.000
AS94_02115	4-oxalocrotonate tautomerase	-1.891	0.000	0.000
AS94_02125	methionine sulfoxide reductase A	-0.396	0.007	0.017
AS94_02145	transcriptional regulator	-0.709	0.001	0.002
AS94_02165	glycerol-3-phosphate acyltransferase	-0.377	0.002	0.006
AS94_02230	XRE family transcriptional regulator	-1.470	0.000	0.000
AS94_02255	50S ribosomal protein L33	-0.422	0.012	0.027
AS94_02270	hypothetical protein	-0.378	0.009	0.022
AS94_02530	tRNA delta(2)-isopentenylpyrophosphate transferase	-0.411	0.020	0.044
AS94_02535	lysophospholipase	-0.821	0.000	0.000
AS94_02540	glycerol-3-phosphate dehydrogenase	-0.747	0.000	0.000
AS94_02590	2-oxoacid ferredoxin oxidoreductase subunit beta	-1.289	0.000	0.000
AS94_02595	2-oxoglutarate ferredoxin oxidoreductase subunitalpha	-1.245	0.000	0.000
AS94_02615	protein RecA	-0.316	0.002	0.005
AS94_02660	cell division protein FtsK	-0.289	0.010	0.024
AS94_02665	ribonuclease J	-0.474	0.000	0.000

AS94_02690	ribosome-binding factor A	-0.560	0.000	0.000
AS94_02720	DNA polymerase III subunit alpha	-0.432	0.000	0.000
AS94_02865	signal recognition particle protein Srp54	-0.786	0.000	0.000
AS94_02930	50S ribosomal protein L28	-1.954	0.000	0.000
AS94_02935	thiamine pyrophosphokinase	-0.441	0.021	0.044
AS94_02990	primosome assembly protein PriA	-0.645	0.000	0.000
AS94_02995	phosphopantothenoylcysteine decarboxylase	-0.662	0.000	0.000
AS94_03000	DNA-directed RNA polymerase subunit omega	-1.050	0.000	0.001
AS94_03005	guanylate kinase	-0.338	0.007	0.017
AS94_03060	uracil phosphoribosyltransferase	-0.512	0.003	0.007
AS94_03075	glyoxalase	-1.127	0.000	0.000
AS94_03080	isoleucyl-tRNA synthetase	-0.993	0.000	0.000
AS94_03170	5'-nucleotidase	-0.493	0.000	0.000
AS94_03205	hypothetical protein	-0.951	0.000	0.000
AS94_03210	carbamate kinase	-2.256	0.000	0.000
AS94_03215	ornithine carbamoyltransferase	-0.686	0.000	0.000
AS94_03290	phosphoesterase	-1.167	0.000	0.000
AS94_03295	deoxyribonucleotide triphosphate pyrophosphatase	-1.136	0.000	0.000
AS94_03300	glutamate racemase	-1.082	0.000	0.000
AS94_03325	thioredoxin	-0.610	0.000	0.000
AS94_03355	phenylalanyl-tRNA synthase subunit beta	-0.694	0.000	0.000
AS94_03425	phosphopantetheine adenylyltransferase	-0.611	0.001	0.002
AS94_03505	inositol monophosphatase	-0.780	0.000	0.000
AS94_03510	hypothetical protein	-0.862	0.000	0.000
AS94_03515	manganese transporter	-0.496	0.000	0.000
AS94_03585	peptide deformylase	-0.322	0.013	0.029
AS94_03590	hypothetical protein	-0.339	0.020	0.044
AS94_03635	SAM-dependent methyltransferase	-0.999	0.000	0.000
AS94_03650	thiamine ABC transporter permease	-0.785	0.000	0.001
AS94_03655	ABC transporter ATP-binding protein	-0.735	0.000	0.000
AS94_03660	cobalt ABC transporter permease	-1.253	0.000	0.000
AS94_03735	quinol oxidase subunit 2	-0.799	0.000	0.000

AS94_03740	quinol oxidase subunit 1	-1.679	0.000	0.000
AS94_03745	cytochrome O ubiquinol oxidase	-2.104	0.000	0.000
AS94_03750	quinol oxidase subunit 4	-2.221	0.000	0.000
AS94_03760	hypothetical protein	-0.488	0.000	0.001
AS94_03765	hypothetical protein	-0.496	0.002	0.006
AS94_03775	mannosyl-glycoprotein endo-beta-N-acetylglucosamidase	-0.465	0.000	0.000
AS94_03780	MarR family transcriptional regulator	-0.851	0.001	0.003
AS94_03795	glutamyl endopeptidase	-1.963	0.000	0.000
AS94_03800	cysteine protease	-1.563	0.000	0.000
AS94_03810	dihydroxynaphthoic acid synthetase	-1.479	0.000	0.000
AS94_03815	2-succinyl-6-hydroxy-2_ 4-cyclohexadiene-1-carboxylate synthase	-1.194	0.000	0.000
AS94_03820	2-succinyl-5-enolpyruvyl-6-hydroxy-3-cyclohexene-1-carboxylate synthase	-0.691	0.000	0.000
AS94_03835	acetyltransferase	-0.783	0.000	0.000
AS94_03870	bacteriocin ABC transporter ATP-binding protein	-1.139	0.000	0.000
AS94_03880	bacteriocin-associated integral membrane protein	-0.742	0.000	0.001
AS94_03900	IDEAL domain protein	-1.069	0.000	0.000
AS94_03925	ATP synthase	-0.550	0.000	0.000
AS94_03940	peptide chain release factor 1	-0.332	0.002	0.006
AS94_03985	enoyl-ACP reductase	-0.356	0.002	0.005
AS94_03995	magnesium transporter MgtE	-0.459	0.001	0.003
AS94_04005	inorganic polyphosphate/ATP-NAD kinase	-0.707	0.000	0.000
AS94_04010	GTP pyrophosphokinase	-0.940	0.000	0.000
AS94_04045	competence negative regulator MecA	-0.782	0.000	0.000
AS94_04130	MAP domain protein	-0.687	0.000	0.000
AS94_04155	DNA methyltransferase	-0.387	0.002	0.004
AS94_04175	hypothetical protein	-0.821	0.000	0.000
AS94_04180	ATP-dependent DNA helicase subunit A	-0.776	0.000	0.000
AS94_04185	ATP-dependent DNA helicase subunit B	-0.820	0.000	0.000
AS94_04215	glucose-6-phosphate isomerase	-0.359	0.004	0.009

AS94_04230	glycerophosphoryl diester phosphodiesterase	-0.461	0.006	0.014
AS94_04245	NADH-dependent flavin oxidoreductase	-0.989	0.000	0.000
AS94_04285	cation:proton antiporter	-0.918	0.000	0.000
AS94_04290	cation:proton antiporter	-2.248	0.000	0.000
AS94_04295	cation:proton antiporter	-1.343	0.000	0.000
AS94_04300	hypothetical protein	-1.642	0.000	0.000
AS94_04305	thioesterase	-0.828	0.000	0.000
AS94_04310	sodium:proton antiporter	-0.666	0.000	0.001
AS94_04325	hypothetical protein	-0.530	0.000	0.001
AS94_04345	nitrogen-fixing protein NifU	-0.444	0.007	0.017
AS94_04375	2-ketogluconate reductase	-0.674	0.000	0.000
AS94_04420	nitronate monooxygenase	-0.347	0.011	0.025
AS94_04505	arsenate reductase	-0.880	0.000	0.000
AS94_04510	thioredoxin	-0.428	0.001	0.003
AS94_04560	membrane protein	-2.984	0.000	0.000
AS94_04580	cold-shock protein	-0.527	0.000	0.000
AS94_04585	thermonuclease	-1.211	0.000	0.000
AS94_04630	enterotoxin I	-0.795	0.000	0.000
AS94_04645	hypothetical protein	-0.548	0.000	0.000
AS94_04660	transposase	-0.890	0.000	0.000
AS94_04665	transposase	-0.806	0.001	0.002
AS94_04695	GTP cyclohydrolase	-0.505	0.000	0.000
AS94_04700	deacetylase	-0.546	0.000	0.000
AS94_04705	hypothetical protein	-0.491	0.001	0.002
AS94_04710	glucosamine-6-phosphate deaminase	-0.606	0.000	0.001
AS94_04720	6-phospho 3-hexuloisomerase	-0.395	0.001	0.004
AS94_04760	pyridoxal kinase	-0.711	0.000	0.000
AS94_04775	membrane protein	-0.527	0.003	0.009
AS94_04805	lipoate-protein ligase A	-0.285	0.009	0.021
AS94_04830	dihydrolipoamide dehydrogenase	-0.456	0.008	0.018
AS94_04890	acetaldehyde reductase	-0.664	0.000	0.000
AS94_04970	cysteine synthase	-0.317	0.005	0.012
AS94_04975	heat shock protein Hsp33	-0.765	0.000	0.000

AS94_04995	hypothetical protein	-0.507	0.000	0.000
AS94_05000	cell division protein DivIC	-0.555	0.003	0.007
AS94_05010	nucleotide pyrophosphohydrolase	-0.789	0.000	0.000
AS94_05035	ribose-phosphate pyrophosphokinase	-0.428	0.000	0.000
AS94_05085	methionyl-tRNA synthetase	-0.471	0.000	0.000
AS94_05105	DNA replication protein YabA	-0.852	0.000	0.000
AS94_05110	signal peptidase II	-0.417	0.001	0.003
AS94_05120	hypothetical protein	-0.742	0.000	0.000
AS94_05125	thymidylate kinase	-0.973	0.000	0.000
AS94_05130	lysine decarboxylase	-0.729	0.000	0.000
AS94_05155	hypothetical protein	-0.915	0.000	0.000
AS94_05290	integrase	-0.353	0.021	0.045
AS94_05410	transposase	-2.258	0.000	0.000
AS94_05425	hypothetical protein	-0.577	0.009	0.021
AS94_05440	NA	-0.566	0.008	0.018
AS94_05445	chromosome partitioning protein ParA	-0.550	0.000	0.000
AS94_05450	multidrug MFS transporter	-0.577	0.000	0.000
AS94_05500	iron citrate ABC transporter substrate-binding protein	-1.250	0.000	0.000
AS94_05515	macrolide MFS transporter	-0.546	0.018	0.039
AS94_05555	alcohol dehydrogenase	-0.802	0.000	0.000
AS94_05560	alcohol dehydrogenase	-0.805	0.000	0.000
AS94_05570	6-phospho-beta-galactosidase	-2.188	0.000	0.000
AS94_05575	PTS lactose transporter subunit IIBC	-1.592	0.000	0.000
AS94_05580	PTS lactose transporter subunit IIA	-1.933	0.000	0.000
AS94_05585	tagatose-bisphosphate aldolase	-1.762	0.000	0.000
AS94_05590	tagatose-6-phosphate kinase	-1.519	0.000	0.000
AS94_05595	galactose-6-phosphate isomerase	-1.210	0.000	0.000
AS94_05600	galactose-6-phosphate isomerase	-1.840	0.000	0.000
AS94_05605	DeoR family transcriptional regulator	-0.556	0.002	0.004
AS94_05615	2_5-diketo-D-gluconic acid reductase	-0.966	0.000	0.000
AS94_05620	MerR family transcriptional regulator	-0.950	0.006	0.014
AS94_05655	alpha-acetolactate decarboxylase	-2.048	0.000	0.000
AS94_05660	acetolactate synthase	-2.238	0.000	0.000

AS94_05705	cobalt ABC transporter ATP-binding protein	-0.542	0.001	0.004
AS94_05870	GNAT family acetyltransferase	-0.834	0.000	0.000
AS94_05880	malonate transporter	-0.922	0.000	0.000
AS94_05890	membrane protein	-0.586	0.000	0.000
AS94_05920	MarR family transcriptional regulator	-0.607	0.001	0.002
AS94_05930	molybdenum cofactor biosynthesis protein A	-0.841	0.000	0.000
AS94_05935	molybdopterin-guanine dinucleotide biosynthesis protein A	-0.839	0.000	0.000
AS94_05945	molybdopterin synthase subunit 2	-1.042	0.000	0.000
AS94_05950	molybdopterin-guanine dinucleotide biosynthesis protein B	-0.747	0.000	0.001
AS94_05955	molybdopterin molybdenumtransferase	-1.877	0.000	0.000
AS94_05960	molybdenum cofactor biosynthesis protein C	-1.289	0.000	0.000
AS94_05965	molybdenum cofactor biosynthesis protein B	-1.775	0.000	0.000
AS94_05970	molybdopterin biosynthesis protein MoeB	-1.200	0.000	0.000
AS94_05975	molybdenum ABC transporter ATP-binding protein	-1.476	0.000	0.000
AS94_05980	molybdenum ABC transporter permease	-1.250	0.000	0.000
AS94_05985	molybdenum ABC transporter substrate-binding protein	-0.388	0.015	0.034
AS94_05990	formate dehydrogenase subunit D	-0.884	0.000	0.000
AS94_06010	ferrichrome ABC transporter substrate-binding protein	-0.714	0.000	0.000
AS94_06015	acyl-CoA dehydrogenase	-0.473	0.003	0.007
AS94_06020	urea transporter	-1.519	0.000	0.000
AS94_06025	urease subunit gamma	-1.313	0.018	0.039
AS94_06030	urease subunit beta	-1.604	0.001	0.001
AS94_06035	urease subunit alpha	-1.104	0.000	0.000
AS94_06040	urease accessory protein UreE	-0.692	0.022	0.047
AS94_06045	urease accessory protein UreF	-1.080	0.000	0.000
AS94_06050	urease accessory protein UreG	-1.076	0.000	0.000
AS94_06055	urease accessory protein UreD	-1.468	0.000	0.000
AS94_06060	MarR family transcriptional regulator	-0.752	0.000	0.000
AS94_06065	hypothetical protein	-0.700	0.000	0.000

AS94_06070	transcriptional regulator	-0.654	0.020	0.042
AS94_06100	hypothetical protein	-1.172	0.000	0.000
AS94_06110	2-hydroxyacid dehydrogenase	-1.415	0.000	0.000
AS94_06115	hypothetical protein	-0.777	0.000	0.000
AS94_06130	hypothetical protein	-1.438	0.000	0.000
AS94_06135	oxidoreductase	-0.751	0.000	0.000
AS94_06155	DeoR family transcriptional regulator	-0.848	0.000	0.000
AS94_06190	membrane protein	-1.640	0.015	0.032
AS94_06195	HAD family hydrolase	-0.655	0.002	0.005
AS94_06210	PTS alpha-glucoside transporter subunit IIBC	-2.416	0.000	0.000
AS94_06215	RpiR family transcriptional regulator	-1.314	0.000	0.000
AS94_06225	sodium:proton antiporter	-0.555	0.000	0.001
AS94_06235	oxidoreductase	-0.582	0.000	0.000
AS94_06265	lysostaphin resistance protein A	-0.943	0.000	0.000
AS94_06270	ribose 5-phosphate isomerase	-1.125	0.000	0.000
AS94_06275	molybdenum cofactor biosysynthesis protein	-0.831	0.000	0.000
AS94_06280	aldose 1-epimerase	-0.382	0.015	0.033
AS94_06285	membrane protein	-0.525	0.018	0.040
AS94_06305	3-methyladenine DNA glycosylase	-1.077	0.000	0.000
AS94_06315	isopentenyl pyrophosphate isomerase	-0.522	0.000	0.000
AS94_06380	hemin ABC transporter ATP-binding protein	-2.303	0.000	0.000
AS94_06385	hemin ABC transporter permease	-2.441	0.000	0.000
AS94_06390	heme transporter CcmC	-0.871	0.001	0.003
AS94_06395	sensor histidine kinase	-1.345	0.000	0.000
AS94_06420	antibiotic ABC transporter permease	-1.124	0.000	0.001
AS94_06445	acetyltransferase	-0.746	0.003	0.007
AS94_06460	GNAT family acetyltransferase	-1.040	0.000	0.000
AS94_06465	ferredoxin--NADP reductase	-1.362	0.000	0.000
AS94_06490	PTS sucrose transporter subunit IIBC	-2.418	0.000	0.000
AS94_06550	LuxR family transcriptional regulator	-0.798	0.000	0.000
AS94_06555	sensor histidine kinase	-0.531	0.000	0.000
AS94_06560	nreA protein	-0.653	0.000	0.000
AS94_06565	nitrate reductase subunit gamma	-0.839	0.000	0.000

AS94_06570	nitrate reductase sununit delta	-1.421	0.000	0.000
AS94_06575	nitrate reductase	-0.926	0.000	0.000
AS94_06580	nitrate reductase	-0.997	0.000	0.000
AS94_06585	uroporphyrinogen III methyltransferase	-1.445	0.000	0.000
AS94_06590	nitrite reductase NAD(P)H small subunit	-1.350	0.000	0.000
AS94_06595	nitrite reductase	-1.209	0.000	0.000
AS94_06600	cobalamin biosynthesis protein CbiX	-0.901	0.000	0.000
AS94_06605	N-acetyltransferase	-0.495	0.010	0.023
AS94_06625	zinc ABC transporter substrate-binding protein	-0.361	0.021	0.045
AS94_06630	protein-disulfide isomerase	-0.496	0.011	0.025
AS94_06665	hypothetical protein	-1.387	0.000	0.000
AS94_06745	hypothetical protein	-1.690	0.000	0.000
AS94_06765	bicyclomycin transporter TcaB	-0.481	0.000	0.001
AS94_06775	hypothetical protein	-0.616	0.000	0.000
AS94_06790	sodium:proton antiporter	-0.398	0.003	0.008
AS94_06795	amino acid permease	-1.036	0.000	0.000
AS94_06805	epimerase	-0.751	0.000	0.000
AS94_06810	2-dehydropantoate 2-reductase	-1.571	0.000	0.000
AS94_06815	quinolone resistance protein NorB	-1.012	0.000	0.000
AS94_06820	amino acid ABC transporter permease	-1.689	0.000	0.000
AS94_06825	glycine/betaine ABC transporter substrate-binding protein	-1.528	0.000	0.000
AS94_06830	choline ABC transporter permease	-0.835	0.000	0.000
AS94_06835	glycine/betaine ABC transporter ATP-binding protein	-0.801	0.000	0.000
AS94_06840	hypothetical protein	-0.705	0.000	0.000
AS94_06845	amino acid:proton symporter	-0.948	0.000	0.000
AS94_06860	membrane protein	-0.989	0.000	0.000
AS94_06870	peptidase M28	-1.211	0.000	0.000
AS94_06875	hypothetical protein	-0.858	0.000	0.000
AS94_06890	antibiotic MFS transporter	-0.510	0.021	0.045
AS94_06955	hypothetical protein	-0.644	0.023	0.049
AS94_06985	hypothetical protein	-1.486	0.000	0.000

AS94_06990	hypothetical protein	-1.159	0.000	0.000
AS94_06995	hypothetical protein	-1.505	0.000	0.000
AS94_07000	hypothetical protein	-0.845	0.000	0.000
AS94_07010	hypothetical protein	-0.764	0.000	0.000
AS94_07040	hypothetical protein	-0.820	0.001	0.003
AS94_07050	UTP--glucose-1-phosphate uridylyltransferase	-0.790	0.000	0.000
AS94_07055	fibronectin-binding protein A	-0.561	0.000	0.001
AS94_07065	gluconate permease	-1.153	0.000	0.000
AS94_07070	gluconokinase	-0.317	0.005	0.012
AS94_07080	MerR family transcriptional regulator	-1.302	0.000	0.000
AS94_07120	fructose-1_6-bisphosphatase	-0.619	0.000	0.000
AS94_07125	membrane protein	-0.347	0.011	0.025
AS94_07130	carboxylesterase	-1.161	0.000	0.000
AS94_07135	glyoxalase	-0.723	0.000	0.001
AS94_07140	MarR family transcriptional regulator	-1.147	0.000	0.000
AS94_07145	acetyltransferase	-0.394	0.024	0.050
AS94_07150	glyoxalase	-0.546	0.001	0.002
AS94_07155	NAD(P)H nitroreductase	-0.788	0.000	0.000
AS94_07165	HAD family hydrolase	-0.712	0.000	0.001
AS94_07180	sortase A	-0.349	0.013	0.029
AS94_07215	esterase	-1.431	0.000	0.000
AS94_07225	acyl-CoA thioester hydrolase	-1.027	0.000	0.000
AS94_07230	PTS glucose transporter subunit IIABC	-1.206	0.000	0.000
AS94_07235	pyruvate oxidase	-1.038	0.000	0.000
AS94_07240	holin	-0.753	0.000	0.000
AS94_07245	holin	-2.964	0.000	0.000
AS94_07250	LysR family transcriptional regulator	-0.730	0.000	0.001
AS94_07255	hypothetical protein	-0.768	0.020	0.043
AS94_07265	hydroxymethylglutaryl-CoA reductase	-0.320	0.013	0.029
AS94_07275	methylated DNA-protein cysteine S-methyltransferase	-0.863	0.000	0.000
AS94_07395	TetR family transcriptional regulator	-0.849	0.000	0.000
AS94_07440	hydrolase	-0.398	0.001	0.003

AS94_07475	fructosamine-3-kinase	-0.860	0.000	0.000
AS94_07510	aspartate decarboxylase	-0.460	0.004	0.011
AS94_07515	pantoate--beta-alanine ligase	-0.400	0.002	0.006
AS94_07520	3-methyl-2-oxobutanoate hydroxymethyltransferase	-0.432	0.006	0.015
AS94_07530	alpha-acetolactate decarboxylase	-0.857	0.000	0.000
AS94_07560	malate:quinone oxidoreductase	-0.522	0.000	0.000
AS94_07570	acyl--CoA ligase	-0.782	0.000	0.000
AS94_07580	hypothetical protein	-0.899	0.010	0.023
AS94_07590	betaine-aldehyde dehydrogenase	-0.668	0.000	0.000
AS94_07595	hypothetical protein	-0.542	0.001	0.003
AS94_07600	choline transporter BetT	-0.655	0.000	0.000
AS94_07605	ribonucleoside-triphosphate reductase activatingprotein	-0.894	0.000	0.000
AS94_07620	precorrin-2 dehydrogenase	-0.828	0.000	0.000
AS94_07625	sulfite reductase subunit alpha	-0.734	0.000	0.000
AS94_07635	peptide ABC transporter permease	-0.667	0.000	0.000
AS94_07675	tributyrin esterase	-0.758	0.000	0.000
AS94_07685	hypothetical protein	-0.966	0.001	0.003
AS94_07690	type I restriction endonuclease subunit S	-0.853	0.000	0.000
AS94_07700	RNA helicase	-0.913	0.000	0.000
AS94_07710	hypothetical protein	-0.729	0.000	0.000
AS94_07715	hypothetical protein	-1.049	0.000	0.000
AS94_07720	transposase	-0.737	0.000	0.000
AS94_07800	Vitamin B12 ABC transporter substrate-binding protein	-0.516	0.003	0.007
AS94_07810	HAD family hydrolase	-0.742	0.001	0.002
AS94_07815	alpha/beta hydrolase	-1.002	0.000	0.000
AS94_07820	hypothetical protein	-1.548	0.000	0.000
AS94_07840	membrane protein	-0.452	0.022	0.047
AS94_07895	sodium:proton antiporter	-0.309	0.012	0.027
AS94_07925	N-acetylmannosaminyltransferase	-0.587	0.001	0.002
AS94_07930	teichoic acid ABC transporter ATP-binding protein	-0.565	0.000	0.000

AS94_07965	nucleoside permease	-0.908	0.000	0.000
AS94_07975	iron-dicitrate ABC transporter ATP-binding protein	-0.977	0.000	0.000
AS94_07980	ferrichrome ABC transporter permease	-0.894	0.000	0.000
AS94_07985	iron ABC transporter permease	-0.956	0.000	0.000
AS94_07995	dihydroxyacetone kinase subunit L	-0.536	0.000	0.001
AS94_08000	PTS mannnose transporter subunit IIA	-1.151	0.000	0.000
AS94_08005	hypothetical protein	-0.573	0.000	0.000
AS94_08010	membrane protein	-0.509	0.000	0.000
AS94_08025	acetyltransferase	-0.890	0.000	0.000
AS94_08030	3-beta hydroxysteroid dehydrogenase	-0.508	0.001	0.002
AS94_08050	bacitracin ABC transporter permease	-0.764	0.000	0.000
AS94_08065	peptidase M23B	-0.580	0.000	0.000
AS94_08070	inhibitor of apoptosis-promoting Bax1	-0.631	0.000	0.000
AS94_08075	AraC family transcriptional regulator	-0.966	0.000	0.000
AS94_08080	MarR family transcriptional regulator	-2.695	0.000	0.000
AS94_08095	hypothetical protein	-0.547	0.000	0.000
AS94_08100	LysR family transcriptional regulator	-0.561	0.000	0.000
AS94_08105	sugar MFS transporter	-0.643	0.000	0.000
AS94_08170	MarR family transcriptional regulator	-0.281	0.005	0.013
AS94_08195	deoxyribodipyrimidine photo-lyase	-0.334	0.003	0.007
AS94_08220	hypothetical protein	-0.999	0.000	0.000
AS94_08225	YbaK/EbsC protein	-1.240	0.000	0.000
AS94_08240	PTS fructose transporter subunit IIC	-0.450	0.002	0.005
AS94_08300	7-cyano-7-deazaguanine synthase	-0.447	0.014	0.030
AS94_08335	heme ABC transporter ATP-binding protein	-0.778	0.000	0.000
AS94_08340	ATP-dependent DNA helicase RecQ	-1.288	0.000	0.000
AS94_08370	lipid kinase	-0.758	0.000	0.000
AS94_08390	ribonucleotide reductase stimulatory protein	-1.517	0.000	0.000
AS94_08395	ribonucleotide-diphosphate reductase subunit alpha	-1.064	0.000	0.000
AS94_08400	ribonucleotide-diphosphate reductase subunit beta	-0.271	0.012	0.028
AS94_08405	iron ABC transporter permease	-1.355	0.000	0.000

AS94_08410	iron ABC transporter permease	-1.251	0.001	0.003
AS94_08485	hypothetical protein	-1.285	0.000	0.000
AS94_08530	excinuclease ABC subunit B	-0.839	0.000	0.000
AS94_08565	hypothetical protein	-0.585	0.002	0.005
AS94_08620	glyceraldehyde-3-phosphate dehydrogenase	-0.583	0.000	0.000
AS94_08635	phosphoglyceromutase	-0.415	0.001	0.003
AS94_08640	enolase	-0.637	0.000	0.000
AS94_08670	hypothetical protein	-0.374	0.003	0.007
AS94_08715	virulence-associated protein E	-0.790	0.001	0.003
AS94_08755	pathogenicity island protein	-0.539	0.000	0.000
AS94_08870	alanine racemase	-0.404	0.001	0.003
AS94_08895	potassium-transporting ATPase subunit C	-1.320	0.000	0.000
AS94_08900	potassium-transporting ATPase subunit B	-0.834	0.000	0.000
AS94_08975	thiamine-phosphate pyrophosphorylase	-0.807	0.008	0.019
AS94_08980	hydroxyethylthiazole kinase	-0.824	0.020	0.043
AS94_09000	single-stranded DNA-binding protein	-0.931	0.002	0.005
AS94_09125	aldehyde dehydrogenase	-0.489	0.000	0.000
AS94_09130	HxlR family transcriptional regulator	-0.328	0.004	0.009
AS94_09135	UDP-N-acetylglucosamine 1-carboxyvinyltransferase	-0.362	0.001	0.003
AS94_09140	fructose-bisphosphate aldolase	-1.039	0.000	0.000
AS94_09145	hypothetical protein	-0.889	0.000	0.000
AS94_09170	hypothetical protein	-0.341	0.015	0.034
AS94_09190	membrane protein	-1.295	0.000	0.000
AS94_09195	pyrimidine-nucleoside phosphorylase	-1.117	0.000	0.000
AS94_09200	deoxyribose-phosphate aldolase	-0.527	0.000	0.000
AS94_09210	general stress protein	-1.297	0.000	0.000
AS94_09235	oxidoreductase	-0.482	0.000	0.001
AS94_09295	FmtB protein	-1.238	0.000	0.000
AS94_09375	MarR family transcriptional regulator	-0.683	0.000	0.000
AS94_09390	iron ABC transporter substrate-binding protein	-1.066	0.001	0.002
AS94_09415	siderophore biosynthesis protein SbnE	-0.589	0.020	0.044
AS94_09430	diaminopimelate decarboxylase	-0.717	0.009	0.021

AS94_09435	siderophore biosynthesis protein SbnI	-0.971	0.000	0.001
AS94_09455	acetoin reductase	-1.887	0.000	0.000
AS94_09485	superoxide dismutase	-2.792	0.000	0.000
AS94_09515	phosphopentomutase	-0.973	0.000	0.000
AS94_09545	2'_ 3'-cyclic nucleotide 2'-phosphodiesterase	-1.224	0.000	0.000
AS94_09640	monooxygenase IsdI	-1.073	0.007	0.016
AS94_09645	hypothetical protein	-0.822	0.016	0.035
AS94_09700	4'-phosphopantetheinyl transferase	-0.669	0.000	0.000
AS94_09705	hypothetical protein	-0.977	0.000	0.000
AS94_09735	isochorismatase	-0.661	0.000	0.000
AS94_09740	pyruvate decarboxylase	-0.590	0.000	0.000
AS94_09750	PTS glucose transporter subunit IIABC	-1.286	0.000	0.000
AS94_09785	ABC transporter ATP-binding protein	-1.172	0.000	0.000
AS94_09815	azoreductase	-0.923	0.000	0.000
AS94_09825	sugar ABC transporter ATP-binding protein	-0.998	0.000	0.001
AS94_09830	ABC transporter substrate-binding protein	-1.004	0.000	0.000
AS94_09835	arabinogalactan ABC transporter permease	-0.991	0.000	0.000
AS94_09840	arabinogalactan ABC transporter permease	-1.504	0.000	0.000
AS94_09845	oxidoreductase	-1.963	0.000	0.000
AS94_09850	NADH-dependent dehydrogenase	-2.303	0.000	0.000
AS94_09855	xylose isomerase	-2.370	0.000	0.000
AS94_09870	AraC family transcriptional regulator	-1.307	0.000	0.000
AS94_09885	formate acetyltransferase	-2.568	0.000	0.000
AS94_09890	pyruvate formate lyase-activating protein	-2.617	0.000	0.000
AS94_09895	hypothetical protein	-1.059	0.004	0.010
AS94_09900	glycerophosphoryl diester phosphodiesterase	-0.478	0.003	0.006
AS94_09980	nitric oxide dioxygenase	-0.419	0.004	0.011
AS94_09990	PTS glucose transporter subunit IIB	-1.257	0.000	0.000
AS94_10030	iditol 2-dehydrogenase	-0.994	0.000	0.000
AS94_10075	cell wall biosynthesis protein ScdA	-0.491	0.000	0.000
AS94_10080	sensor histidine kinase	-0.717	0.000	0.000
AS94_10085	LytR family transcriptional regulator	-0.829	0.000	0.001
AS94_10100	GntR family transcriptional regulator	-0.977	0.000	0.000

AS94_10105	phosphoenolpyruvate-dependent sugar PTS family porter_ EIIA 1	-2.224	0.000	0.000
AS94_10110	aryl-phospho-beta-D-glucosidase	-2.039	0.000	0.000
AS94_10115	hypothetical protein	-1.335	0.000	0.000
AS94_10140	LacI family transcriptional regulator	-1.483	0.000	0.000
AS94_10155	penicillin V acylase	-0.848	0.000	0.000
AS94_10160	glycyl-glycine endopeptidase	-0.675	0.000	0.000
AS94_10255	hypothetical protein	-0.400	0.019	0.040
AS94_10320	formate/nitrite transporter	-0.602	0.000	0.000
AS94_10340	peptide ABC transporter ATP-binding protein	-0.437	0.001	0.002
AS94_10355	pseudouridine-5'-phosphate glycosidase	-0.616	0.002	0.005
AS94_10365	sialic acid transporter	-0.743	0.000	0.000
AS94_10370	N-acetylneuraminate lyase	-0.910	0.000	0.000
AS94_10375	N-acetylmannosamine kinase	-0.500	0.000	0.000
AS94_10380	RpiR family transcriptional regulator	-0.540	0.000	0.000
AS94_10385	N-acetylmannosamine-6-phosphate 2-epimerase	-0.813	0.000	0.000
AS94_10400	alpha/beta hydrolase	-0.946	0.000	0.000
AS94_10475	glyoxalase	-0.553	0.010	0.022
AS94_10480	luciferase	-1.150	0.000	0.000
AS94_10485	FMN reductase	-0.957	0.000	0.000
AS94_10490	membrane protein	-1.031	0.000	0.000
AS94_10665	hypothetical protein	-1.321	0.000	0.000
AS94_10675	phosphoglycerate mutase	-0.416	0.020	0.044
AS94_10700	alkyl hydroperoxide reductase subunit C	-0.366	0.001	0.002
AS94_10705	NADPH-dependent oxidoreductase	-0.712	0.000	0.000
AS94_10730	xanthine phosphoribosyltransferase	-1.523	0.000	0.000
AS94_10735	xanthine permease	-1.247	0.000	0.000
AS94_10740	inosine 5'-monophosphate dehydrogenase	-1.206	0.000	0.000
AS94_10745	GMP synthase	-1.191	0.000	0.000
AS94_10815	membrane protein	-1.961	0.000	0.000
AS94_10820	3-beta hydroxysteroid dehydrogenase	-1.140	0.000	0.000
AS94_10890	hypothetical protein	-1.002	0.000	0.000

AS94_10895	hypothetical protein	-1.503	0.002	0.005
AS94_10940	NADH dehydrogenase subunit 5	-0.926	0.000	0.000
AS94_10945	hypothetical protein	-0.629	0.000	0.000
AS94_10965	carboxylesterase	-0.800	0.000	0.000
AS94_10970	sodium-dependent transporter	-0.903	0.000	0.000
AS94_11000	N-acetylmuramoyl-L-alanine amidase	-0.457	0.000	0.000
AS94_11020	hypothetical protein	-1.007	0.000	0.000
AS94_11025	hypothetical protein	-0.535	0.002	0.004
AS94_11075	DNA polymerase III subunit gamma/tau	-0.370	0.002	0.004
AS94_11080	hypothetical protein	-0.499	0.013	0.029
AS94_11085	recombinase RecR	-0.384	0.012	0.026
AS94_11090	adhesin	-1.330	0.000	0.000
AS94_11095	2-oxoglutarate translocator	-0.947	0.000	0.000
AS94_11100	chloramphenicol-sensitive protein RarD	-0.508	0.003	0.007
AS94_11110	nickel transporter NixA	-0.893	0.000	0.000
AS94_11115	N-acetyltransferase	-0.821	0.000	0.000
AS94_11145	cold-shock protein	-0.592	0.000	0.000
AS94_11150	Cro/Cl family transcriptional regulator	-1.032	0.000	0.000
AS94_11155	hypothetical protein	-0.629	0.002	0.006
AS94_11160	hypothetical protein	-0.695	0.001	0.003
AS94_11225	carbohydrate kinase	-1.238	0.000	0.000
AS94_11265	50S ribosomal protein L9	-0.666	0.000	0.000
AS94_11270	replicative DNA helicase	-1.089	0.000	0.000
AS94_11305	hypothetical protein	-0.388	0.000	0.001
AS94_11400	recombinase RecA	-0.828	0.000	0.000
AS94_11465	branched-chain amino acid aminotransferase	-0.363	0.001	0.002
AS94_11480	molecular chaperone	-1.156	0.000	0.000
AS94_11490	N-acetyl-L_L-diaminopimelate deacetylase	-0.456	0.003	0.008
AS94_11495	elongation factor Tu	-0.374	0.001	0.003
AS94_11520	DNA-directed RNA polymerase subunit beta'	-0.472	0.000	0.000
AS94_11525	DNA-directed RNA polymerase subunit beta	-0.271	0.015	0.033
AS94_11600	glutamyl-tRNA synthase	-0.585	0.000	0.000
AS94_11660	adhesin	-2.529	0.000	0.000

AS94_11670	pyrrolidone-carboxylate peptidase	-1.052	0.000	0.000
AS94_11690	polysaccharide deacetylase	-1.007	0.002	0.006
AS94_11695	ATP phosphoribosyltransferase	-2.797	0.000	0.000
AS94_11700	ATP phosphoribosyltransferase	-2.726	0.000	0.000
AS94_11705	histidinol dehydrogenase	-2.226	0.000	0.000
AS94_11710	histidinol-phosphate aminotransferase	-1.979	0.000	0.000
AS94_11715	imidazoleglycerol-phosphate dehydratase	-1.428	0.020	0.044
AS94_11720	imidazole glycerol phosphate synthase	-1.895	0.001	0.002
AS94_11725	1-(5-phosphoribosyl)-5-[(5-phosphoribosylamino)methylideneamino] imidazole-4-carboxamide isomerase	-1.252	0.001	0.002
AS94_11730	imidazole glycerol phosphate synthase	-0.932	0.005	0.013
AS94_11735	phosphoribosyl-AMP cyclohydrolase	-0.752	0.001	0.003
AS94_11760	N-glycosyltransferase	-1.757	0.000	0.000
AS94_11800	hypothetical protein	-0.383	0.005	0.012
AS94_11830	adhesin	-0.674	0.000	0.000
AS94_11835	preprotein translocase subunit SecY	-1.093	0.000	0.000
AS94_11840	Accessory Sec system protein Asp1	-1.249	0.000	0.000
AS94_11845	accessory secretory protein Asp2	-1.085	0.000	0.000
AS94_11850	Accessory Sec system protein Asp3	-0.775	0.009	0.022
AS94_11890	mannose-6-phosphate isomerase	-3.259	0.000	0.000
AS94_11895	PTS mannose transporter subunit IIABC	-2.737	0.000	0.000
AS94_11905	hypothetical protein	-0.564	0.006	0.015
AS94_11925	arginine deiminase	-1.290	0.000	0.000
AS94_11930	ornithine carbamoyltransferase	-0.449	0.001	0.004
AS94_11960	Fur family transcriptional regulator	-0.644	0.000	0.000
AS94_11980	membrane protein	-0.296	0.023	0.049
AS94_12005	membrane protein	-0.661	0.000	0.000
AS94_12405	hypothetical protein	-0.387	0.009	0.021
AS94_12435	methionine aminopeptidase	-0.422	0.001	0.004
AS94_12470	ferritin	-0.897	0.000	0.000
AS94_12565	membrane protein	-0.648	0.000	0.000
AS94_12570	NAD synthetase	-0.443	0.002	0.005
AS94_12585	prephenate dehydratase	-0.617	0.000	0.000

AS94_12605		pyrophosphatase	-0.864	0.000	0.000
AS94_12610		aldehyde dehydrogenase	-0.845	0.000	0.000
AS94_12670		hypothetical protein	-0.801	0.000	0.000
AS94_12785		hypothetical protein	-1.746	0.000	0.000
AS94_12815		peptidase M23	-0.945	0.000	0.000
AS94_12865		nitroreductase	-0.400	0.002	0.006
AS94_12875	*hld*	delta-hemolysin	-0.296	0.004	0.010
AS94_12880	*agrB*	accessory gene regulator B	-0.784	0.000	0.000
AS94_12890	*agrC*	histidine kinase	-0.536	0.000	0.000
AS94_12895	*agrA*	histidine kinase	-0.901	0.000	0.000
AS94_12900		fructokinase	-1.091	0.000	0.000
AS94_12905		sucrose-6-phosphate hydrolase	-0.423	0.000	0.000
AS94_12930		redox-sensing transcriptional repressor Rex	-0.258	0.022	0.046
AS94_12945		O-sialoglycoprotein endopeptidase	-0.444	0.000	0.001
AS94_13015		hypothetical protein	-3.340	0.017	0.036
AS94_13445		protoporphyrinogen oxidase	-0.459	0.001	0.004
AS94_13475		cell-cycle regulation protein HIT	-1.246	0.000	0.000
AS94_13480		hypothetical protein	-0.558	0.000	0.000
AS94_13560		glucosamine-6-phosphate isomerase	-0.322	0.002	0.005
AS94_13585		membrane protein	-0.484	0.015	0.034
AS94_12195		transcriptional regulator	-0.864	0.005	0.012
AS94_12245		hypothetical protein	-1.619	0.014	0.030
AS94_12265	ϕSA169	hypothetical protein	-1.225	0.000	0.000
AS94_12285		DNA replication protein DnaC	-0.648	0.001	0.003
AS94_12360		transcriptional regulator	-0.419	0.006	0.015
AS94_13030		hypothetical protein	-1.393	0.000	0.000
AS94_13035		enterotoxin	-0.712	0.001	0.003
AS94_13070		autolysin	-0.873	0.000	0.000
AS94_13075		holin	-1.556	0.000	0.000
AS94_13095	mutual prophage	hypothetical protein	-0.651	0.002	0.006
AS94_13100		minor structural protein	-0.542	0.001	0.003
AS94_13110		peptidase	-0.459	0.021	0.044
AS94_13120		tail protein	-0.489	0.000	0.000
AS94_13135		tail protein	-0.647	0.000	0.001

AS94_13195	transcriptional regulator	-1.194	0.000	0.000
AS94_13200	helicase	-0.662	0.002	0.006
AS94_13205	hypothetical protein	-0.686	0.001	0.002
AS94_13220	hypothetical protein	-1.089	0.000	0.000
AS94_13350	hypothetical protein	-1.035	0.016	0.036
AS94_13360	hypothetical protein	-1.755	0.000	0.000
AS94_13370	hypothetical protein	-0.611	0.000	0.000
AS94_13375	XRE family transcriptional regulator	-0.882	0.000	0.001
AS94_13390	hypothetical protein	-0.551	0.000	0.000
