# Supplementary material for: Transcriptome Analyses of Prophage in Mediating Persistent Methicillin-Resistant Staphylococcus aureus Endovascular Infection
Source: Genes (Basel). 2022 Aug 25;13(9):1527. doi: 10.3390/genes13091527 (PMC9498598; doi:10.3390/genes13091527)
Supplement: Supplementary file 1 [file genes-13-01527-s001.zip › Table S7.pdf]

Table S7. Up-regulated DEGs in both 300-169 vs. 301-188 and 300-169 vs. 301-188::ϕSA169

							**300-169 vs. 301-188**			**300-169 vs. 301-188::ϕSA169**		
**locus**	**gene**	**group**	**product**	***agr*-regulated (Y)**	**sarA-regulated (Y)**	**sigB-regulated (Y)**	**log_2(fold change)**	***p* value**	***p* adj**	**log_2(fold change)**	***p* value**	***p* adj**
AS94_00015			hypothetical protein				0.979	0.000	0.000	0.660	0.003	0.007
AS94_00025			calcium-binding protein				1.265	0.000	0.000	1.736	0.000	0.000
AS94_00060	*pckA*		phosphoenolpyruvate carboxykinase		Y		0.596	0.000	0.000	1.067	0.000	0.000
AS94_00150			Diaminohydroxyphospho-ribosylaminopyrimidine deaminase				1.209	0.000	0.000	0.875	0.000	0.000
AS94_00155	*ribD*		riboflavin synthase subunit alpha	Y			0.838	0.000	0.000	0.706	0.001	0.002
AS94_00160	*ribE*		GTP cyclohydrolase	Y			0.831	0.000	0.000	0.537	0.001	0.002
AS94_00170	*ribA*		proline dehydrogenase	Y			0.863	0.000	0.000	1.615	0.000	0.000
AS94_00175			lysophospholipase				0.575	0.000	0.000	0.756	0.000	0.000
AS94_00180	*rot*		MarR family transcriptional regulator				0.666	0.000	0.000	0.865	0.000	0.000
AS94_00275			hypothetical protein				1.099	0.000	0.000	0.824	0.000	0.000
AS94_00280			tRNA-binding protein				1.274	0.000	0.000	1.016	0.000	0.000
AS94_00285			cell division protein FtsK				1.122	0.000	0.000	1.082	0.000	0.000
AS94_00290	*murC*	host genes	UDP-N-acetylmuramate--alanine ligase			Y	0.808	0.000	0.000	0.757	0.000	0.000
AS94_00295			hypothetical protein				0.999	0.000	0.000	0.733	0.000	0.000
AS94_00300			smooth muscle caldesmon				1.170	0.000	0.000	1.244	0.000	0.000
AS94_00325			acetyl-CoA synthetase				0.516	0.000	0.000	1.010	0.000	0.000
AS94_00330	*fhs*		formate--tetrahydrofolate ligase		Y		0.281	0.017	0.036	0.454	0.000	0.000
AS94_00350			serine protease				1.256	0.000	0.000	1.577	0.000	0.000
AS94_00390	*rpsD*		30S ribosomal protein S4				0.617	0.000	0.000	0.373	0.001	0.002
AS94_00430	*ackA*		acetate kinase				0.460	0.000	0.000	0.426	0.000	0.000
AS94_00440	*ald*		alanine dehydrogenase				0.595	0.000	0.000	0.949	0.000	0.000
AS94_00530	*phoP*		PhoP family transcriptional regulator				0.349	0.024	0.049	0.419	0.007	0.016
AS94_00560	*gap*		glyceraldehyde-3-phosphate dehydrogenase				0.674	0.000	0.000	1.143	0.000	0.000
AS94_00590			translation initiation factor IF-3				1.200	0.000	0.000	1.000	0.000	0.000
AS94_00595	*rpmI*		50S ribosomal protein L35				1.376	0.000	0.000	1.059	0.000	0.000

AS94_00600	*rplT*	50S ribosomal protein L20	1.353	0.000	0.000	1.117	0.000	0.000
AS94_00605		DNA mismatch repair protein MutT	0.476	0.015	0.031	0.660	0.001	0.002
AS94_00615		trigger factor	1.057	0.000	0.000	0.854	0.000	0.000
AS94_00620		ATP-dependent protease	0.740	0.000	0.000	0.503	0.000	0.000
AS94_00625		GTP-binding protein YsxC	0.620	0.002	0.004	0.661	0.001	0.002
AS94_00630	*hemA*	glutamyl-tRNA reductase	0.366	0.000	0.001	0.704	0.000	0.000
AS94_00635		cytochrome C assembly protein	0.707	0.000	0.000	0.951	0.000	0.000
AS94_00640	*hemC*	porphobilinogen deaminase	0.816	0.000	0.000	0.989	0.000	0.000
AS94_00660		aminopeptidase	0.345	0.009	0.021	0.487	0.000	0.001
AS94_00700		membrane protein	3.845	0.000	0.000	4.341	0.000	0.000
AS94_00705		hypothetical protein	4.743	0.000	0.000	4.081	0.000	0.000
AS94_00710		rod shape-determining protein MreC	0.865	0.000	0.000	0.883	0.000	0.000
AS94_00715		rod shape-determining protein MreD	0.839	0.000	0.000	0.811	0.000	0.000
AS94_00760		queuine tRNA-ribosyltransferase	0.546	0.000	0.000	0.340	0.006	0.014
AS94_00815		hypothetical protein	0.338	0.007	0.016	0.284	0.024	0.050
AS94_00830		hypothetical protein	1.817	0.000	0.000	1.334	0.000	0.000
AS94_00910		allophanate hydrolase subunit 1	2.559	0.000	0.000	2.336	0.000	0.000
AS94_00915		allophanate hydrolase subunit 2	2.558	0.000	0.000	2.139	0.000	0.000
AS94_00920	*rnfC*	acetyl-CoA carboxylase	2.132	0.000	0.000	1.965	0.000	0.000
AS94_00925		acetyl-CoA carboxylase biotin carboxylase subunit	2.006	0.000	0.000	2.097	0.000	0.000
AS94_00930	*lamB/YcsF*	hypothetical protein	1.816	0.000	0.000	2.053	0.000	0.000
AS94_00935		iron transporter	1.412	0.000	0.000	1.804	0.000	0.000
AS94_00955	*mtnN*	S-adenosylhomocysteine nucleosidase	1.082	0.000	0.000	1.166	0.000	0.000
AS94_00960		hypothetical protein	1.271	0.000	0.000	1.194	0.000	0.000
AS94_00965		GTPase	1.316	0.000	0.000	1.265	0.000	0.000
AS94_00970	*aroE*	shikimate 5-dehydrogenase	1.238	0.000	0.000	1.269	0.000	0.000
AS94_00980	*nadD*	nicotinic acid mononucleotide adenylyltransferase	1.237	0.000	0.000	1.241	0.000	0.000
AS94_00985		HAD family hydrolase	1.203	0.000	0.000	1.207	0.000	0.000

AS94_00990		Iojap family protein	1.158	0.000	0.000	1.229	0.000	0.000
AS94_00995		methyltransferase	1.162	0.000	0.000	0.906	0.000	0.000
AS94_01020	*rpsT*	30S ribosomal protein S20	0.813	0.000	0.000	0.520	0.000	0.000
AS94_01035		HrcA family transcriptional regulator	0.382	0.003	0.007	0.522	0.000	0.000
AS94_01040		heat shock protein GrpE	0.332	0.007	0.017	0.650	0.000	0.000
AS94_01050		molecular chaperone DnaJ	0.880	0.000	0.000	0.961	0.000	0.000
AS94_01055		ribosomal protein L11 methyltransferase	1.069	0.000	0.000	1.068	0.000	0.000
AS94_01060		16S rRNA methyltransferase	0.938	0.000	0.000	0.843	0.000	0.000
AS94_01070	*rpsU*	30S ribosomal protein S21	0.750	0.000	0.000	0.488	0.001	0.003
AS94_01075		serine protease	0.726	0.000	0.000	0.870	0.000	0.000
AS94_01080		hypothetical protein	0.434	0.000	0.001	0.458	0.000	0.000
AS94_01085		iron transporter	0.476	0.000	0.000	0.510	0.000	0.000
AS94_01120	*glyS*	glycyl-tRNA synthetase	1.455	0.000	0.000	1.346	0.000	0.000
AS94_01145		SAM-dependent methyltransferase	0.715	0.000	0.000	0.488	0.004	0.010
AS94_01190		penicillin-binding protein 3	0.412	0.001	0.002	0.428	0.000	0.001
AS94_01195	*rpmG*	50S ribosomal protein L33	0.880	0.000	0.001	0.679	0.005	0.011
AS94_01200		5-formyltetrahydrofolate cyclo-ligase	0.544	0.009	0.019	0.881	0.000	0.000
AS94_01205		membrane protein	0.761	0.000	0.000	0.926	0.000	0.000
AS94_01215		glucokinase	0.731	0.000	0.000	0.797	0.000	0.000
AS94_01220		hypothetical protein	0.830	0.001	0.004	0.789	0.003	0.006
AS94_01225		hydroxyacylglutathione hydrolase	0.715	0.000	0.000	0.588	0.000	0.000
AS94_01305		elongation factor P	0.635	0.000	0.000	0.499	0.000	0.000
AS94_01310	*accB*	acetyl-CoA carboxylase	0.851	0.000	0.000	0.721	0.000	0.000
AS94_01315	*accC*	acetyl-CoA carboxylase biotin carboxylase subunit	0.847	0.000	0.000	0.754	0.000	0.000
AS94_01320		hypothetical protein	0.790	0.000	0.000	0.689	0.000	0.000
AS94_01325		transcription antitermination protein NusB	0.587	0.001	0.002	0.624	0.000	0.001
AS94_01330	*xseA*	exodeoxyribonuclease VII large subunit	0.691	0.000	0.000	0.719	0.000	0.000
AS94_01340		geranyltranstransferase	0.603	0.000	0.000	0.730	0.000	0.000
AS94_01345		arginine repressor ArgR	1.174	0.000	0.000	1.479	0.000	0.000

AS94_01350		DNA repair protein RecN		1.368	0.000	0.000	1.672	0.000	0.000
AS94_01355	*lpdA*	dihydrolipoamide dehydrogenase		0.664	0.000	0.000	0.839	0.000	0.000
AS94_01375		hypothetical protein		0.830	0.000	0.000	0.615	0.001	0.002
AS94_01380		membrane protein		0.676	0.000	0.000	0.468	0.001	0.004
AS94_01485	*srrA*	PhoP family transcriptional regulator		0.497	0.000	0.000	0.788	0.000	0.000
AS94_01495		hypothetical protein		1.190	0.000	0.000	1.538	0.000	0.000
AS94_01500		hypothetical protein		1.027	0.000	0.000	1.402	0.000	0.000
AS94_01505		hypothetical protein		0.570	0.007	0.016	0.680	0.001	0.004
AS94_01520		hypothetical protein		0.418	0.022	0.046	0.543	0.003	0.008
AS94_01525		ATP-dependent DNA helicase RecQ		0.465	0.003	0.007	0.528	0.001	0.002
AS94_01530		peptidoglycan-binding protein LysM		0.259	0.015	0.031	0.307	0.004	0.009
AS94_01550	*rpsA*	30S ribosomal protein S1		0.414	0.000	0.000	0.572	0.000	0.000
AS94_01585	*ndk*	nucleoside diphosphate kinase		0.787	0.000	0.000	0.908	0.000	0.000
AS94_01655	*asnS*	asparaginyl-tRNA synthase		0.561	0.000	0.000	0.647	0.000	0.000
AS94_01675		transglycosylase		1.190	0.000	0.000	0.938	0.000	0.000
AS94_01680		recombinase RecU		1.159	0.000	0.000	0.957	0.000	0.000
AS94_01685		hypothetical protein		0.913	0.000	0.000	0.939	0.000	0.000
AS94_01690		hypothetical protein		1.081	0.000	0.000	1.112	0.000	0.000
AS94_01695		cell cycle protein GpsB		1.211	0.000	0.000	1.240	0.000	0.000
AS94_01705		RNA methyltransferase		1.002	0.000	0.000	0.776	0.000	0.000
AS94_01710		hypothetical protein		0.892	0.000	0.001	0.838	0.001	0.002
AS94_01715		sulfite reductase subunit alpha		1.860	0.000	0.000	1.736	0.000	0.000
AS94_01720		dynamin family protein		0.696	0.000	0.000	0.862	0.000	0.000
AS94_01785	*thyA*	thymidylate synthase		0.675	0.000	0.000	0.640	0.000	0.000
AS94_01830	*murG*	UDP-diphospho-muramoylpentapeptide beta-N- acetylglucosaminyltransferase	Y	0.617	0.000	0.000	0.716	0.000	0.000
AS94_01835		phosphatidic acid phosphatase		0.783	0.000	0.000	0.712	0.000	0.000
AS94_01855	*sucA*	2-oxoglutarate dehydrogenase E1		0.400	0.000	0.000	0.749	0.000	0.000
AS94_01860	*sucB*	dihydrolipoamide succinyltransferase		0.266	0.012	0.026	0.503	0.000	0.000
AS94_01890		hypothetical protein		0.760	0.000	0.000	0.510	0.005	0.012

AS94_01895		nitric oxide reductase activation protein NorD			0.444	0.001	0.002	0.440	0.001	0.002
AS94_01905		tellurite resistance protein TelA			0.509	0.000	0.000	0.632	0.000	0.000
AS94_01910		5-bromo-4-chloroindolyl phosphate hydrolysis protein			0.560	0.001	0.004	0.643	0.000	0.001
AS94_01915		acylphosphatase			0.727	0.000	0.001	0.711	0.000	0.001
AS94_01970	*asd*	aspartate-semialdehyde dehydrogenase	Y		0.689	0.007	0.017	0.788	0.002	0.006
AS94_01975		aspartate kinase			0.920	0.001	0.003	1.040	0.000	0.001
AS94_01990	*pstS*	thioredoxine reductase			1.098	0.007	0.016	1.610	0.000	0.000
AS94_02120		LytR family transcriptional regulator			1.111	0.000	0.000	0.923	0.000	0.000
AS94_02130	*mprF*	phosphatidylglycerol lysyltransferase			0.502	0.000	0.000	0.491	0.000	0.000
AS94_02155		DNA topoisomerase IV subunit A			0.486	0.000	0.000	0.352	0.002	0.005
AS94_02160		DNA topoisomerase IV subunit B			0.661	0.000	0.000	0.552	0.000	0.000
AS94_02175		4-hydroxybenzoyl-CoA thioesterase			0.549	0.000	0.000	0.518	0.000	0.000
AS94_02180	*acnA*	aconitate hydratase			0.769	0.000	0.000	0.738	0.000	0.000
AS94_02185		choline transporter			0.898	0.000	0.000	0.664	0.000	0.000
AS94_02240		secretion protein			0.922	0.000	0.000	0.883	0.000	0.000
AS94_02245	*guaC*	guanosine 5'-monophosphate oxidoreductase			1.619	0.000	0.000	1.455	0.000	0.000
AS94_02275		HAD family hydrolase			0.310	0.014	0.029	0.367	0.004	0.009
AS94_02280		homoserine kinase			0.840	0.000	0.000	0.931	0.000	0.000
AS94_02285	*thrB*	threonine synthase	Y		0.453	0.001	0.004	0.589	0.000	0.000
AS94_02290	*thrC*	homoserine dehydrogenase	Y		0.629	0.000	0.000	1.028	0.000	0.000
AS94_02295		aspartate kinase			1.302	0.000	0.000	1.786	0.000	0.000
AS94_02300		hypothetical protein			0.713	0.000	0.001	0.735	0.000	0.001
AS94_02560	*mutL*	DNA mismatch repair protein MutL		Y	0.448	0.000	0.001	0.631	0.000	0.000
AS94_02565	*mutS*	DNA mismatch repair protein MutS		Y	0.538	0.000	0.000	0.494	0.000	0.000
AS94_02635		hypothetical protein			0.311	0.023	0.046	0.424	0.002	0.005
AS94_02645		zinc protease			0.461	0.003	0.007	0.371	0.016	0.036
AS94_02675	*rpsO*	30S ribosomal protein S15			0.598	0.000	0.000	0.308	0.004	0.009
AS94_02685		tRNA pseudouridine synthase B			0.498	0.006	0.014	0.497	0.006	0.015

AS94_02700		50S ribosomal protein L7		0.404	0.021	0.043	0.624	0.000	0.001
AS94_02710		transcription elongation factor NusA		0.535	0.000	0.000	0.691	0.000	0.000
AS94_02715		ribosome maturation protein RimP		0.588	0.000	0.000	0.884	0.000	0.000
AS94_02725	*proS*	prolyl-tRNA synthetase		0.658	0.000	0.000	0.509	0.000	0.000
AS94_02730		zinc metalloprotease		0.744	0.000	0.000	0.662	0.000	0.000
AS94_02735		phosphatidate cytidylyltransferase		0.544	0.000	0.000	0.535	0.000	0.001
AS94_02740	*uppS*	UDP pyrophosphate synthase		0.939	0.000	0.000	0.891	0.000	0.000
AS94_02755		elongation factor Ts		1.044	0.000	0.000	1.115	0.000	0.000
AS94_02760	*rpsB*	30S ribosomal protein S2		1.301	0.000	0.000	1.029	0.000	0.000
AS94_02765		transcriptional regulator		0.812	0.000	0.000	0.678	0.000	0.000
AS94_02770		ATP-dependent protease		0.999	0.000	0.000	1.064	0.000	0.000
AS94_02775		ATP-dependent protease		1.002	0.000	0.000	1.054	0.000	0.000
AS94_02780		tyrosine recombinase XerC		1.153	0.000	0.000	1.247	0.000	0.000
AS94_02785		tRNA (uracil-5-)-methyltransferase		0.658	0.000	0.000	0.500	0.000	0.000
AS94_02790		DNA topoisomerase I		0.853	0.000	0.000	0.542	0.000	0.000
AS94_02815	*sucD*	succinyl-CoA synthetase subsunit alpha		0.794	0.000	0.000	0.905	0.000	0.000
AS94_02820	*sucC*	malate--CoA ligase subunit beta	Y	1.079	0.000	0.000	1.209	0.000	0.000
AS94_02845	*rplS*	50S ribosomal protein L19		0.873	0.000	0.000	0.613	0.000	0.000
AS94_02880		chromosome segregation protein SMC		0.313	0.008	0.017	0.423	0.000	0.001
AS94_02890	*acpP*	acyl carrier protein		0.624	0.000	0.000	0.341	0.018	0.038
AS94_02905	*plsX*	phosphate acyltransferase		0.676	0.000	0.000	0.597	0.000	0.000
AS94_02910		transcription factor		0.920	0.000	0.000	0.953	0.000	0.000
AS94_02920		hypothetical protein		0.820	0.000	0.000	0.695	0.000	0.000
AS94_02925		hypothetical protein		0.454	0.001	0.004	0.375	0.008	0.019
AS94_02945	*rsgA*	GTPase		0.416	0.005	0.013	0.476	0.002	0.004
AS94_03015		3-demethylubiquinone-9 3-methyltransferase		0.446	0.004	0.009	0.586	0.000	0.000
AS94_03025	*pyrE*	orotate phosphoribosyltransferase		1.225	0.000	0.000	1.077	0.000	0.000
AS94_03030	*pyrF*	orotidine 5'-phosphate decarboxylase		1.430	0.000	0.000	1.367	0.000	0.000

AS94_03035	*carB*	carbamoyl phosphate synthase large subunit			1.017	0.000	0.000	1.053	0.000	0.000
AS94_03040	*carA*	carbamoyl phosphate synthase small subunit	Y		0.724	0.001	0.002	0.581	0.006	0.014
AS94_03115		cell division protein FtsZ			0.639	0.000	0.000	0.604	0.000	0.000
AS94_03120		cell division protein FtsA			0.745	0.000	0.000	0.822	0.000	0.000
AS94_03125		cell division protein FtsQ			0.878	0.000	0.000	0.928	0.000	0.000
AS94_03130	*murD*	UDP-N-acetylmuramoyl-L-alanyl-D-glutamate synthetase		Y	0.874	0.000	0.000	0.934	0.000	0.000
AS94_03135	*mraY*	phospho-N-acetylmuramoyl-pentapeptide-transferase			0.862	0.000	0.000	0.826	0.000	0.000
AS94_03140		penicillin-binding protein 1			1.055	0.000	0.000	1.170	0.000	0.000
AS94_03145		cell division protein FtsL			0.851	0.000	0.000	0.888	0.000	0.000
AS94_03150		16S rRNA methyltransferase			1.112	0.000	0.000	1.212	0.000	0.000
AS94_03155		cell division protein MraZ			1.223	0.000	0.000	1.280	0.000	0.000
AS94_03165		hypothetical protein			0.912	0.000	0.000	1.215	0.000	0.000
AS94_03175		hypothetical protein			1.653	0.000	0.001	1.307	0.003	0.007
AS94_03180		hypothetical protein			1.556	0.001	0.003	1.479	0.002	0.005
AS94_03195		ethanolamine utilization protein EutQ			0.891	0.000	0.000	1.162	0.000	0.000
AS94_03245		leukocidin/Hemolysin toxin family protein			2.663	0.000	0.000	3.250	0.000	0.000
AS94_03310	*sdhA*	succinate dehydrogenase flavoprotein subunit			0.483	0.000	0.001	0.464	0.001	0.002
AS94_03315	*sdhC*	succinate dehydrogenase cytochrome B558			0.552	0.000	0.000	0.894	0.000	0.000
AS94_03330	*mutS2*	DNA mismatch repair protein MutS		Y	0.661	0.000	0.000	0.697	0.000	0.000
AS94_03335		DNA polymerase			0.684	0.000	0.000	0.666	0.000	0.000
AS94_03460		membrane protein			0.330	0.012	0.025	0.796	0.000	0.000
AS94_03465	*cyoE*	protoheme IX farnesyltransferase			0.489	0.000	0.000	0.918	0.000	0.000
AS94_03470	*ctaA*	heme A synthase			0.634	0.000	0.000	1.041	0.000	0.000
AS94_03495		GTP-binding protein			0.801	0.000	0.000	0.529	0.000	0.000
AS94_03530		spermidine/putrescine ABC transporter substrate-binding protein			0.732	0.000	0.000	0.755	0.000	0.000
AS94_03600		potassium transporter Trk			0.917	0.000	0.000	0.799	0.000	0.000
AS94_03605		cytochrome D ubiquinol oxidase subunit II			2.267	0.000	0.000	2.006	0.000	0.000
AS94_03610		cytochrome D ubiquinol oxidase subunit I			2.164	0.000	0.000	2.035	0.000	0.000

AS94_03620	*ptsI*	phosphoenolpyruvate-protein phosphotransferase			0.790	0.000	0.000	0.676	0.000	0.000
AS94_03625	*ptsH*	phosphocarrier protein HPr			0.650	0.000	0.000	0.818	0.000	0.000
AS94_03630		hypothetical protein			0.791	0.000	0.000	0.659	0.000	0.000
AS94_03640		membrane protein			0.810	0.000	0.000	0.657	0.000	0.000
AS94_03665	*purD*	phosphoribosylamine--glycine ligase			0.763	0.000	0.000	0.727	0.000	0.000
AS94_03670	*purH*	purine biosynthesis protein purH			1.316	0.000	0.000	1.399	0.000	0.000
AS94_03675	*purN*	phosphoribosylglycinamide formyltransferase			1.580	0.000	0.000	1.815	0.000	0.000
AS94_03680	*purM*	phosphoribosylaminoimidazole synthetase			1.747	0.000	0.000	1.666	0.000	0.000
AS94_03685	*purF*	amidophosphoribosyltransferase			1.476	0.000	0.000	1.530	0.000	0.000
AS94_03690	*purL*	phosphoribosylformylglycinamidine synthase			1.275	0.000	0.000	1.235	0.000	0.000
AS94_03695	*purQ*	phosphoribosylformylglycinamidine synthase		Y	1.063	0.000	0.001	0.766	0.007	0.016
AS94_03710	*purK*	phosphoribosylaminoimidazole carboxylase		Y	1.272	0.000	0.000	0.802	0.002	0.006
AS94_03720	*folD*	tetrahydrofolate dehydrogenase		Y	0.734	0.000	0.000	0.820	0.000	0.000
AS94_03730		chitinase			0.998	0.000	0.000	1.288	0.000	0.000
AS94_03755		methicillin resistance protein FmtA			1.096	0.000	0.000	0.938	0.000	0.000
AS94_03885		CAAX amino terminal protease			0.972	0.000	0.000	0.522	0.004	0.009
AS94_03965		hypothetical protein			0.692	0.000	0.000	0.913	0.000	0.000
AS94_04055	*trpS*	tryptophanyl-tRNA synthetase			0.433	0.001	0.002	0.309	0.018	0.039
AS94_04085		peptide ABC transporter substrate-binding protein			0.579	0.000	0.000	0.857	0.000	0.000
AS94_04090		peptide ABC transporter ATP-binding protein			0.747	0.000	0.001	0.781	0.000	0.001
AS94_04095		peptide ABC transporter ATP-binding protein			0.695	0.002	0.006	0.841	0.000	0.001
AS94_04100		peptide ABC transporter permease			0.794	0.000	0.000	0.798	0.000	0.000
AS94_04105		peptide ABC transporter permease			0.991	0.000	0.000	0.892	0.000	0.000
AS94_04115	*fabF*	3-oxoacyl-ACP synthase	Y		0.719	0.000	0.000	0.295	0.012	0.027
AS94_04120	*fabH*	3-oxoacyl-ACP synthase	Y		1.095	0.000	0.000	0.517	0.001	0.002
AS94_04170		hypothetical protein			0.867	0.000	0.000	0.505	0.005	0.013
AS94_04190	*lepB*	signal peptidase IB			1.090	0.000	0.000	1.066	0.000	0.000
AS94_04195	*lepB*	signal peptidase I			1.056	0.000	0.000	1.031	0.000	0.000

AS94_04200		hypothetical protein			0.632	0.000	0.000	0.433	0.007	0.016
AS94_04220	*argG*	argininosuccinate synthase	Y	Y	6.222	0.000	0.000	6.152	0.000	0.000
AS94_04225	*argH*	argininosuccinate lyase	Y	Y	5.619	0.000	0.000	5.631	0.000	0.000
AS94_04240	*rocD*	ornithine-oxoacid aminotransferase		Y	0.351	0.001	0.003	0.680	0.000	0.000
AS94_04320		NADH dehydrogenase			0.899	0.000	0.000	0.734	0.000	0.000
AS94_04350	*dltD*	D-alanyl-lipoteichoic acid biosynthesis protein DltD	Y		0.797	0.000	0.000	0.752	0.000	0.000
AS94_04360	*dltB*	D-alanyl transfer protein DltB	Y		0.780	0.000	0.000	0.718	0.000	0.000
AS94_04365	*dltA*	D-alanine--poly(phosphoribitol) ligase	Y		0.872	0.000	0.000	0.968	0.000	0.000
AS94_04405		5'-nucleotidase			0.552	0.000	0.000	0.507	0.000	0.001
AS94_04410		membrane protein			0.580	0.002	0.005	0.513	0.006	0.015
AS94_04450		Fe-S cluster assembly protein SufD			0.594	0.000	0.000	0.535	0.000	0.000
AS94_04455		iron ABC transporter ATP-binding protein			0.651	0.000	0.000	0.820	0.000	0.000
AS94_04460		hypothetical protein			0.945	0.000	0.000	0.824	0.000	0.000
AS94_04485		thioredoxin			0.846	0.000	0.000	0.494	0.023	0.049
AS94_04490		topiosmerase			0.640	0.002	0.005	0.653	0.002	0.004
AS94_04540		phosphoglycerate mutase			1.008	0.000	0.000	0.940	0.000	0.000
AS94_04550		hypothetical protein			1.038	0.000	0.000	0.812	0.000	0.001
AS94_04605	*clfA*	clumping factor A	Y	Y	0.877	0.000	0.000	0.694	0.000	0.000
AS94_04635	*entC3*	enterotoxin			0.311	0.004	0.010	0.775	0.000	0.000
AS94_04655		hypothetical protein			0.459	0.000	0.001	0.752	0.000	0.000
AS94_04670	*sdrC*	hydrolase	Y	Y	1.617	0.000	0.000	1.770	0.000	0.000
AS94_04725		haloacid dehalogenase			1.509	0.000	0.000	1.456	0.000	0.000
AS94_04730		glycine/betaine MFS transporter			1.488	0.000	0.000	1.381	0.000	0.000
AS94_04735		long-chain fatty acid--CoA ligase			4.180	0.000	0.000	3.781	0.000	0.000
AS94_04740		acetyl-CoA acetyltransferase			2.242	0.000	0.000	2.229	0.000	0.000
AS94_04745		vraC			1.973	0.001	0.002	3.127	0.000	0.000
AS94_04750		hypothetical protein			3.222	0.000	0.000	3.366	0.000	0.000
AS94_04755		vraX			3.049	0.000	0.000	3.312	0.000	0.000
AS94_04780		amino acid permease			1.289	0.000	0.000	0.782	0.000	0.000

AS94_04810	*mvk*	mevalonate kinase	Y	1.594	0.000	0.000	1.382	0.000	0.000
AS94_04815	*mvaD*	diphosphomevalonate decarboxylase	Y	1.814	0.000	0.000	1.502	0.000	0.000
AS94_04820		phosphomevalonate kinase		1.673	0.000	0.000	1.298	0.000	0.000
AS94_04840		transposase		0.710	0.000	0.000	0.745	0.000	0.000
AS94_04865		oxidoreductase ion channel protein IolS		0.386	0.008	0.018	0.429	0.003	0.008
AS94_04950	*lysS*	lysyl-tRNA synthetase		0.452	0.000	0.000	0.428	0.000	0.000
AS94_04955	*folK*	2-amino-4-hydroxy-6-hydroxymethyldihydropteridine pyrophosphokinase		1.104	0.000	0.000	1.123	0.000	0.000
AS94_04960	*folB*	dihydroneopterin aldolase		1.683	0.000	0.000	1.843	0.000	0.000
AS94_04965	*folP*	dihydropteroate synthase		2.204	0.000	0.000	2.032	0.000	0.000
AS94_04980		zinc metalloprotease		0.610	0.000	0.000	0.368	0.000	0.001
AS94_04985	*hpt*	hypoxanthine phosphoribosyltransferase		0.524	0.000	0.001	0.367	0.009	0.022
AS94_05140		hypothetical protein		0.718	0.002	0.005	0.541	0.020	0.043
AS94_05340		replication initiation factor family protein		2.706	0.013	0.028	3.139	0.004	0.010
AS94_05345		hypothetical protein		2.940	0.016	0.033	3.206	0.008	0.019
AS94_05350		hypothetical protein		3.020	0.014	0.031	3.347	0.007	0.016
AS94_05355		hypothetical protein		3.042	0.016	0.034	3.487	0.006	0.014
AS94_05435		integrase		1.176	0.009	0.021	1.211	0.008	0.018
AS94_05465		hemolysin III		0.562	0.000	0.000	0.763	0.000	0.000
AS94_05470		uridylyltransferase		0.526	0.000	0.000	0.742	0.000	0.000
AS94_05475		membrane protein		0.402	0.006	0.015	0.671	0.000	0.000
AS94_05480		hypothetical protein		0.399	0.001	0.002	0.698	0.000	0.000
AS94_05485		membrane protein		2.663	0.000	0.000	2.482	0.000	0.000
AS94_05525	*asp23*	alkaline shock protein 23		1.326	0.000	0.000	1.013	0.000	0.000
AS94_05530		membrane protein		0.988	0.000	0.000	0.845	0.000	0.000
AS94_05535		hypothetical protein		0.950	0.000	0.000	0.691	0.000	0.000
AS94_05540		glycine/betaine ABC transporter permease		1.635	0.000	0.000	1.196	0.000	0.000
AS94_05625		hyaluronate lyase		0.555	0.002	0.004	0.558	0.002	0.004
AS94_05650		toxin		1.123	0.000	0.000	1.240	0.000	0.000

AS94_05680	*rpsI*	30S ribosomal protein S9			1.190	0.000	0.000	0.961	0.000	0.000
AS94_05685	*rplM*	50S ribosomal protein L13			1.212	0.000	0.000	1.063	0.000	0.000
AS94_05785	*rplE*	50S ribosomal protein L5			0.333	0.013	0.027	0.452	0.001	0.002
AS94_05790	*rplX*	50S ribosomal protein L24			0.436	0.002	0.005	0.643	0.000	0.000
AS94_05800	*rpsQ*	30S ribosomal protein S17			0.567	0.003	0.007	0.698	0.000	0.001
AS94_05810	*rplP*	50S ribosomal protein L16			0.513	0.000	0.001	0.540	0.000	0.000
AS94_05815	*rpsC*	30S ribosomal protein S3			0.501	0.000	0.001	0.601	0.000	0.000
AS94_05820	*rplV*	50S ribosomal protein L22			0.396	0.005	0.013	0.524	0.000	0.001
AS94_05825	*rpsS*	30S ribosomal protein S19			0.439	0.004	0.009	0.607	0.000	0.000
AS94_05830	*rplB*	50S ribosomal protein L2			0.464	0.000	0.001	0.452	0.000	0.001
AS94_05840	*rplD*	50S ribosomal protein L4			0.427	0.001	0.003	0.377	0.004	0.010
AS94_05845	*rplC*	50S ribosomal protein L3			0.615	0.000	0.000	0.609	0.000	0.000
AS94_05850	*rpsJ*	30S ribosomal protein S10			0.939	0.000	0.000	0.723	0.000	0.000
AS94_06080		hypothetical protein			1.519	0.000	0.000	0.859	0.000	0.000
AS94_06085		sodium:proton antiporter			0.882	0.000	0.000	0.517	0.000	0.001
AS94_06145		LytR family transcriptional regulator			0.714	0.000	0.000	0.788	0.000	0.000
AS94_06170		RpiR family transcriptional regulator			0.363	0.006	0.014	0.335	0.011	0.025
AS94_06205		hypothetical protein			2.047	0.000	0.000	1.951	0.000	0.000
AS94_06245	*hutI*	imidazolonepropionase		Y	2.049	0.000	0.000	2.024	0.000	0.000
AS94_06250	*hutU*	urocanate hydratase	Y	Y	1.987	0.000	0.000	2.078	0.000	0.000
AS94_06300		hypothetical protein			0.877	0.000	0.000	1.044	0.000	0.000
AS94_06310		sodium:glutamate symporter			1.092	0.000	0.000	0.613	0.000	0.000
AS94_06325		3-hydroxyacyl-CoA dehydrogenase			0.555	0.014	0.030	0.582	0.010	0.023
AS94_06365		membrane protein			1.854	0.000	0.000	1.731	0.000	0.000
AS94_06425	*mqo*	malate:quinone oxidoreductase			0.620	0.000	0.000	0.872	0.000	0.000
AS94_06450		quinone oxidoreductase			0.523	0.000	0.001	0.365	0.010	0.023
AS94_06655		amino acid ABC transporter substrate-binding protein			0.566	0.000	0.000	0.523	0.000	0.001
AS94_06670	*gpmA*	phosphoglyceromutase			0.512	0.000	0.000	0.821	0.000	0.000
AS94_06680	*sbi*	hypothetical protein	Y	Y	1.422	0.000	0.000	1.886	0.000	0.000

AS94_06685	*hlgA*	gamma-hemolysin subunit A			0.618	0.005	0.011	0.956	0.000	0.000
AS94_06755		glycerate kinase			1.650	0.000	0.000	1.762	0.000	0.000
AS94_06760		membrane protein			1.748	0.000	0.000	1.999	0.000	0.000
AS94_06770		transcriptional regulator			1.261	0.000	0.000	1.084	0.001	0.002
AS94_06855		chloramphenicol resistance protein DHA1			0.710	0.000	0.001	0.578	0.002	0.006
AS94_06940		short-chain dehydrogenase			0.676	0.000	0.000	0.425	0.001	0.003
AS94_06945		aminobenzoyl-glutamate transporter			1.029	0.000	0.000	1.048	0.000	0.000
AS94_07095		membrane protein			0.710	0.000	0.000	0.345	0.015	0.032
AS94_07100		glucarate transporter			0.367	0.019	0.040	0.578	0.000	0.001
AS94_07190	*sdaAA*	serine dehydratase subunit alpha	Y		1.350	0.000	0.000	1.378	0.000	0.000
AS94_07195	*sdaAB*	serine dehydratase	Y		1.304	0.000	0.000	1.527	0.000	0.000
AS94_07200		transcriptional regulator			1.123	0.000	0.000	1.288	0.000	0.000
AS94_07270		hydroxymethylglutaryl-CoA synthase			1.456	0.000	0.000	1.378	0.000	0.000
AS94_07280		Clp protease ATP-binding protein			1.513	0.000	0.000	1.294	0.000	0.000
AS94_07310		1-pyrroline-5-carboxylate dehydrogenase			0.884	0.000	0.000	1.261	0.000	0.000
AS94_07320		hypothetical protein			3.453	0.000	0.000	4.145	0.000	0.000
AS94_07350	*crtM*	dehydrosqualene synthase	Y	Y	1.071	0.000	0.000	0.874	0.000	0.000
AS94_07355	*crtQ*	4_4'-diaponeurosporenoate glycosyltransferase	Y	Y	0.774	0.000	0.000	0.602	0.001	0.002
AS94_07360	*crtP*	diapolycopene oxygenase		Y	0.709	0.000	0.000	0.589	0.000	0.001
AS94_07480	*pyrD*	dihydroorotate dehydrogenase			0.910	0.000	0.000	0.991	0.000	0.000
AS94_07540		amino acid permease			1.179	0.000	0.000	1.069	0.000	0.000
AS94_07545		4-aminobutyrate aminotransferase			1.004	0.003	0.007	1.378	0.000	0.000
AS94_07745		Replication and maintenance protein			14.571	0.000	0.000	14.474	0.000	0.000
AS94_07750		SAM-dependent methlyltransferase			15.751	0.000	0.000	15.654	0.000	0.000
AS94_07835	*sarA*	transcriptional regulator			0.785	0.000	0.000	0.621	0.000	0.000
AS94_07855		recombinase			0.844	0.000	0.001	0.542	0.015	0.033
AS94_07860		cation:proton antiporter			1.176	0.000	0.000	0.930	0.000	0.000
AS94_07865		cation:proton antiporter			1.088	0.000	0.001	1.002	0.001	0.003
AS94_07870		cation:proton antiporter			1.431	0.000	0.000	1.711	0.000	0.000

AS94_07875		cation:proton antiporter	1.387	0.000	0.000	1.466	0.000	0.000
AS94_07880		cation:proton antiporter	1.154	0.002	0.006	1.602	0.000	0.000
AS94_07890		cation:proton antiporter	1.142	0.000	0.000	1.534	0.000	0.000
AS94_07900		manganese ABC transporter substrate-binding protein	0.683	0.000	0.000	1.328	0.000	0.000
AS94_07905	*fecCD*	membrane protein	0.545	0.000	0.000	1.233	0.000	0.000
AS94_07910		phosphonate ABC transporter ATP-binding protein	0.862	0.000	0.000	1.616	0.000	0.000
AS94_07920		membrane protein	1.800	0.000	0.000	1.875	0.000	0.000
AS94_08150		hypothetical protein	0.818	0.000	0.000	0.884	0.000	0.000
AS94_08155	*uppP*	UDP pyrophosphate phosphatase	1.261	0.000	0.000	0.818	0.000	0.000
AS94_08160		cysteine ABC transporter ATP-binding protein	0.405	0.001	0.001	0.456	0.000	0.000
AS94_08165		cysteine ABC transporter ATP-binding protein	0.549	0.000	0.000	0.633	0.000	0.000
AS94_08190		membrane protein	0.539	0.000	0.000	0.854	0.000	0.000
AS94_08215		multidrug MFS transporter	0.685	0.000	0.000	0.549	0.000	0.000
AS94_08255		glyoxal reductase	0.753	0.000	0.000	0.603	0.000	0.000
AS94_08330	*ltaS1*	glycerol phosphate lipoteichoic acid synthase	1.264	0.000	0.000	1.274	0.000	0.000
AS94_08350		ABC transporter permease	0.530	0.000	0.000	0.345	0.006	0.015
AS94_08375		peptide ABC transporter permease	0.279	0.020	0.042	0.447	0.000	0.001
AS94_08470		membrane protein	0.738	0.000	0.000	0.675	0.000	0.000
AS94_08480		hypothetical protein	1.192	0.000	0.000	0.888	0.000	0.000
AS94_08575		hypothetical protein	0.803	0.000	0.000	0.777	0.000	0.000
AS94_08580		sporulation protein	0.500	0.000	0.001	0.537	0.000	0.000
AS94_08590		ATP-dependent Clp protease proteolytic subunit	0.457	0.000	0.000	0.617	0.000	0.000
AS94_08610		hypothetical protein	0.466	0.000	0.000	0.436	0.000	0.001
AS94_08615		transcriptional regulator	0.486	0.000	0.000	0.515	0.000	0.000
AS94_08675		integrase	0.448	0.003	0.007	0.555	0.000	0.001
AS94_08685		hypothetical protein	3.481	0.000	0.000	1.686	0.014	0.030
AS94_08810		NA	1.493	0.000	0.000	1.507	0.000	0.000
AS94_08960	*cls*	phospholipase D	0.656	0.000	0.000	0.564	0.000	0.000

AS94_08965	*oxaA*	phosphohydrolase		0.530	0.000	0.001	0.516	0.001	0.002
AS94_08970		membrane protein		0.613	0.000	0.000	0.290	0.015	0.033
AS94_09015	*murA*	UDP-N-acetylglucosamine 1-carboxyvinyltransferase	Y	0.562	0.000	0.000	0.379	0.001	0.004
AS94_09020		membrane protein		1.061	0.013	0.028	0.970	0.023	0.049
AS94_09040	*atpA*	F0F1 ATP synthase subunit alpha		0.694	0.000	0.000	0.654	0.000	0.000
AS94_09045	*atpH*	F0F1 ATP synthase subunit delta		0.533	0.000	0.000	0.627	0.000	0.000
AS94_09050	*atpF*	F0F1 ATP synthase subunit B		0.776	0.000	0.000	0.895	0.000	0.000
AS94_09055	*atpE*	F0F1 ATP synthase subunit C		0.318	0.021	0.042	0.383	0.005	0.013
AS94_09060	*atpB*	F0F1 ATP synthase subunit A		0.435	0.000	0.000	0.465	0.000	0.000
AS94_09065		ATP synthase		0.419	0.006	0.013	0.592	0.000	0.000
AS94_09070		UDP-N-acetylglucosamine 2-epimerase		0.710	0.000	0.000	0.854	0.000	0.000
AS94_09075	*upp*	uracil phosphoribosyltransferase		0.716	0.000	0.000	0.793	0.000	0.000
AS94_09080	*glyA*	serine hydroxymethyltransferase		0.589	0.000	0.000	0.792	0.000	0.000
AS94_09085		hypothetical protein		0.661	0.000	0.000	0.872	0.000	0.000
AS94_09100		N5-glutamine S-adenosyl-L-methionine-dependent methyltransferase		0.552	0.000	0.000	0.541	0.000	0.000
AS94_09105		peptide chain release factor 1		0.684	0.000	0.000	0.557	0.000	0.000
AS94_09115	*rpmE*	50S ribosomal protein L31		0.378	0.002	0.006	0.289	0.020	0.044
AS94_09150	*pyrG*	CTP synthetase		1.261	0.000	0.000	0.987	0.000	0.000
AS94_09160		acetyltransferase		1.323	0.000	0.000	0.884	0.000	0.000
AS94_09165	*coaA*	pantothenate kinase		0.898	0.000	0.000	0.645	0.001	0.003
AS94_09240		ArsR family transcriptional regulator		1.854	0.000	0.000	1.904	0.000	0.000
AS94_09245		cation transporter		1.422	0.000	0.000	1.318	0.000	0.000
AS94_09250		lytic regulatory protein		0.641	0.000	0.000	0.743	0.000	0.000
AS94_09275	*mtlA*	PTS mannitol transporter subunit IIB	Y	1.502	0.000	0.001	1.842	0.000	0.000
AS94_09280		PTS lactose transporter subunit IIB		1.979	0.000	0.000	1.918	0.000	0.000
AS94_09285	*mtlF*	PTS mannitol transporter subunit IIA	Y	1.613	0.000	0.000	1.500	0.000	0.000
AS94_09290	*mtlD*	mannitol-1-phosphate 5-dehydrogenase	Y	1.814	0.000	0.000	1.509	0.000	0.000
AS94_09315	*rocF*	arginase		0.803	0.000	0.000	0.880	0.000	0.000

AS94_09450		membrane protein			0.766	0.008	0.018	0.844	0.004	0.009
AS94_09490		surface protein			0.567	0.000	0.000	0.634	0.000	0.000
AS94_09560		capsular polysaccharide biosynthesis protein CapA			1.554	0.000	0.000	1.641	0.000	0.000
AS94_09565		capsular polysaccharide biosynthesis protein Cap5B			1.706	0.000	0.000	1.808	0.000	0.000
AS94_09570		capsular polysaccharide biosynthesis protein Cap8C			1.635	0.000	0.000	1.647	0.000	0.000
AS94_09575		polysaccharide biosynthesis protein EpsC			1.580	0.000	0.000	1.611	0.000	0.000
AS94_09580		UDP-glucose 4-epimerase			1.728	0.000	0.000	1.840	0.000	0.000
AS94_09585		capsular polysaccharide biosynthesis protein Cap8F			1.526	0.000	0.000	1.717	0.000	0.000
AS94_09590		UDP-N-acetylglucosamine 2-epimerase			1.646	0.000	0.000	1.877	0.000	0.000
AS94_09595		capsular polysaccharide biosynthesis protein			1.591	0.000	0.000	1.991	0.000	0.000
AS94_09600		capsular polysaccharide biosynthesis protein			1.343	0.000	0.000	1.620	0.000	0.000
AS94_09605		capsular polysaccharide biosynthesis protein			1.211	0.000	0.000	1.536	0.000	0.000
AS94_09610		capsular polysaccharide biosynthesis protein			0.896	0.001	0.001	1.070	0.000	0.000
AS94_09615		glycosyltransferase family 1			0.556	0.000	0.001	0.774	0.000	0.000
AS94_09630	*wecC*	UDP-N-acetyl-D-mannosamine dehydrogenase			0.311	0.025	0.049	0.571	0.000	0.000
AS94_09690		multidrug MFS transporter			0.882	0.000	0.000	1.144	0.000	0.000
AS94_09755		cellobiose operon outer surface protein			0.638	0.000	0.000	1.293	0.000	0.000
AS94_09760	*murQ*	N-acetylmuramic acid-6-phosphate etherase	Y	Y	0.578	0.000	0.000	1.205	0.000	0.000
AS94_09765		permease			0.676	0.000	0.000	1.432	0.000	0.000
AS94_09770		RpiR family transcriptional regulator			0.881	0.000	0.000	1.547	0.000	0.000
AS94_09820		peptidase M23			1.093	0.000	0.000	0.956	0.000	0.000
AS94_09865		antiporter			2.494	0.000	0.000	2.802	0.000	0.000
AS94_09920		3-hydroxyacyl-CoA dehydrogenase			0.879	0.001	0.001	1.118	0.000	0.000
AS94_09925		glutaryl-CoA dehydrogenase			1.155	0.003	0.006	1.385	0.000	0.001
AS94_09930		long-chain fatty acid--CoA ligase			1.533	0.000	0.000	1.974	0.000	0.000
AS94_09935		coenzyme A transferase			1.762	0.000	0.000	2.050	0.000	0.000
AS94_09955		NmrA family protein			2.177	0.000	0.000	2.065	0.000	0.000
AS94_09960		DeoR family transcriptional regulator			1.806	0.000	0.000	1.475	0.000	0.000

AS94_09985		lactate dehydrogenase			0.788	0.000	0.000	1.252	0.000	0.000
AS94_09995		inosine-uridine preferring nucleoside hydrolase			0.623	0.002	0.004	0.524	0.008	0.018
AS94_10035	*ispD1*	2-C-methyl-D-erythritol 4-phosphate cytidylyltransferase			0.982	0.000	0.000	0.961	0.000	0.000
AS94_10040		ribitol-5-phosphate dehydrogenase			0.721	0.001	0.002	0.720	0.001	0.003
AS94_10045		teichoic acid biosynthesis protein			0.748	0.000	0.000	0.961	0.000	0.000
AS94_10050		CDP-glycerol:glycerophosphate glycerophosphotransferase			0.775	0.000	0.000	0.422	0.016	0.034
AS94_10125	*rbsK*	ribokinase		Y	2.808	0.000	0.000	3.000	0.000	0.000
AS94_10130	*rbsD*	ribose pyranase		Y	2.726	0.000	0.000	3.101	0.000	0.000
AS94_10135		ribose transporter RbsU			2.853	0.000	0.000	3.014	0.000	0.000
AS94_10200		virulence factor EsxA			0.390	0.001	0.002	0.490	0.000	0.000
AS94_10205		type VII secretion protein EsaA			0.914	0.000	0.000	0.886	0.000	0.000
AS94_10220		type VII secretion protein EssB			0.596	0.024	0.049	0.904	0.001	0.002
AS94_10315		hypothetical protein			0.745	0.000	0.001	0.863	0.000	0.000
AS94_10325		branched-chain amino acid transporter II carrierprotein			0.968	0.000	0.000	0.668	0.000	0.000
AS94_10390		histidine transporter			2.081	0.000	0.000	2.250	0.000	0.000
AS94_10395	*lip2*	lipase		Y	0.498	0.000	0.000	0.939	0.000	0.000
AS94_10470		sn-glycerol-3-phosphate transporter			1.821	0.000	0.000	1.983	0.000	0.000
AS94_10555	*thlA*	acetyl-CoA acetyltransferase			0.837	0.000	0.000	0.778	0.000	0.000
AS94_10565	*metE*	5-methyltetrahydropteroyltriglutamate--homocysteine methyltransferase	Y		0.852	0.000	0.000	0.777	0.000	0.000
AS94_10570		5_10-methylenetetrahydrofolate reductase			0.983	0.000	0.000	0.913	0.000	0.000
AS94_10575		cystathionine beta-lyase			0.840	0.013	0.028	1.129	0.001	0.003
AS94_10580	*metB*	cystathionine gamma-synthase			0.782	0.015	0.032	1.239	0.000	0.001
AS94_10600		GTP-binding protein YchF			0.305	0.024	0.049	0.486	0.000	0.001
AS94_10660		peptidase			1.829	0.000	0.000	1.600	0.000	0.000
AS94_10670		membrane protein			0.941	0.000	0.000	0.649	0.000	0.000
AS94_10725		hypothetical protein			0.541	0.000	0.000	0.638	0.000	0.000
AS94_10785		growth inhibitor PemK			0.719	0.000	0.000	0.716	0.000	0.000
AS94_10800		hypothetical protein			0.786	0.000	0.000	1.022	0.000	0.000

AS94_10935		cobalamin synthesis protein CobW				2.380	0.000	0.000	2.349	0.000	0.000
AS94_10985	*metN*	methionine ABC transporter ATP-binding protein		Y		1.535	0.000	0.000	1.379	0.000	0.000
AS94_11070		acetyltransferase				0.620	0.009	0.019	0.671	0.005	0.011
AS94_11170		16S rRNA methyltransferase				0.540	0.000	0.001	0.654	0.000	0.000
AS94_11175		tRNA uridine 5-carboxymethylaminomethyl modification protein				0.569	0.000	0.000	0.622	0.000	0.000
AS94_11180		tRNA modification GTPase				0.481	0.000	0.001	0.465	0.001	0.002
AS94_11185		ribonuclease P				0.873	0.000	0.001	0.738	0.003	0.008
AS94_11190	*rpmH*	50S ribosomal protein L34				0.924	0.000	0.000	0.691	0.000	0.000
AS94_11195	*dnaA*	chromosome replication protein DnaA				0.548	0.000	0.000	0.676	0.000	0.000
AS94_11200	*dnaN*	DNA polymerase III subunit beta				0.635	0.000	0.000	0.677	0.000	0.000
AS94_11210	*recF*	recombinase F				0.481	0.000	0.000	0.737	0.000	0.000
AS94_11215		DNA gyrase subunit B				0.557	0.000	0.000	0.798	0.000	0.000
AS94_11220		DNA topoisomerase IV subunit A				0.725	0.000	0.000	0.757	0.000	0.000
AS94_11230	*hutH*	histidine ammonia-lyase			Y	0.628	0.000	0.000	0.796	0.000	0.000
AS94_11235	*serS*	seryl-tRNA synthetase				1.022	0.000	0.000	1.149	0.000	0.000
AS94_11275	*purA*	adenylosuccinate synthetase		Y		2.591	0.000	0.000	2.120	0.000	0.000
AS94_11320		50S rRNA methyltransferase				0.509	0.001	0.002	0.816	0.000	0.000
AS94_11355	*mecA*	penicillin-binding protein	Y			0.635	0.000	0.000	0.836	0.000	0.000
AS94_11360	*mecR1*	methicillin resistance protein	Y			0.415	0.006	0.014	0.420	0.005	0.013
AS94_11430		hydrolase				2.561	0.000	0.000	2.577	0.000	0.000
AS94_11470		UDP-glucose 4-epimerase				0.795	0.000	0.000	1.274	0.000	0.000
AS94_11485		2-amino-3-ketobutyrate CoA ligase				0.544	0.000	0.000	0.753	0.000	0.000
AS94_11530		16S rRNA methyltransferase				0.767	0.000	0.000	0.752	0.000	0.000
AS94_11535	*rplL*	50S ribosomal protein L7/L12		Y		0.976	0.000	0.000	0.922	0.000	0.000
AS94_11540	*rplJ*	50S ribosomal protein L10		Y		1.082	0.000	0.000	1.025	0.000	0.000
AS94_11545	*rplA*	50S ribosomal protein L1				1.176	0.000	0.000	0.948	0.000	0.000
AS94_11550	*rplK*	50S ribosomal protein L11				0.711	0.000	0.000	0.572	0.000	0.000
AS94_11575		hypothetical protein				0.417	0.015	0.031	0.637	0.000	0.001

AS94_11610		DNA repair protein RadA			0.355	0.005	0.012	0.344	0.007	0.016
AS94_11615		ATP-dependent Clp protease ATP-binding protein			0.396	0.000	0.000	0.477	0.000	0.000
AS94_11665		DNA damage-inducible protein DinB			0.614	0.000	0.001	0.399	0.021	0.045
AS94_11680		lactonase			0.993	0.000	0.000	1.005	0.000	0.000
AS94_11740	*lip*	lipase		Y	1.297	0.000	0.000	1.830	0.000	0.000
AS94_11765		transcriptional regulator			0.602	0.000	0.000	0.620	0.000	0.000
AS94_11815		hypothetical protein			0.695	0.004	0.009	0.719	0.003	0.007
AS94_11825		flavin reductase			0.795	0.001	0.002	0.948	0.000	0.000
AS94_11855	*secA2*	preprotein translocase subunit SecA			0.320	0.012	0.027	0.381	0.003	0.007
AS94_11870		surface anchored protein			0.933	0.000	0.000	1.032	0.000	0.000
AS94_11875		isochorismatase hydrolase			0.341	0.005	0.012	0.363	0.003	0.007
AS94_11880		N-acetylmuramoyl-L-alanine amidase			0.521	0.000	0.000	0.606	0.000	0.000
AS94_11940	*arcC*	carbamate kinase	Y		0.510	0.000	0.001	0.738	0.000	0.000
AS94_11945		Crp/Fnr family transcriptional regulator			0.580	0.000	0.000	0.736	0.000	0.000
AS94_11985		multidrug ABC transporter ATP-binding protein			0.925	0.000	0.000	0.508	0.000	0.000
AS94_11990		hypothetical protein			0.695	0.000	0.000	0.619	0.000	0.000
AS94_12020		RecX family transcriptional regulator			0.925	0.000	0.000	0.815	0.000	0.000
AS94_12025	*mgt*	glycosyltransferase			2.944	0.000	0.000	2.930	0.000	0.000
AS94_12030		general stress protein			0.703	0.000	0.000	0.329	0.002	0.006
AS94_12410		ribonuclease BN			1.228	0.000	0.000	0.808	0.000	0.000
AS94_12415	*vraR*	LuxR family transcriptional regulator			3.187	0.000	0.000	3.056	0.000	0.000
AS94_12420	*vraS*	sensor histidine kinase			3.380	0.000	0.000	3.494	0.000	0.000
AS94_12425		transporter			3.431	0.000	0.000	3.384	0.000	0.000
AS94_12430		hypothetical protein			3.841	0.000	0.000	3.562	0.000	0.000
AS94_12465	*mur*	UDP-N-acetylmuramate--alanine ligase			0.705	0.000	0.000	0.425	0.001	0.004
AS94_12490		RNA methyltransferase			0.594	0.000	0.000	0.577	0.000	0.000
AS94_12545	*purB*	adenylosuccinate lyase			0.810	0.000	0.000	0.732	0.000	0.000
AS94_12550		cysteine protease			1.797	0.000	0.000	2.179	0.000	0.000

AS94_12555			staphostatin A		1.108	0.001	0.004	1.670	0.000	0.000
AS94_12590			C4-dicarboxylate ABC transporter		0.379	0.006	0.014	0.585	0.000	0.000
AS94_12630	*blaR1*		beta-lactamase		0.440	0.017	0.036	0.535	0.004	0.009
AS94_12700			membrane protein		1.446	0.000	0.000	1.133	0.000	0.000
AS94_12705			antibiotic ABC transporter ATP-binding protein		1.061	0.000	0.000	0.870	0.000	0.000
AS94_12710			membrane protein		1.386	0.000	0.001	1.031	0.006	0.015
AS94_12715			sodium ABC transporter ATP-binding protein		1.491	0.000	0.000	1.381	0.000	0.000
AS94_12720			GntR family transcriptional regulator		1.459	0.000	0.000	1.258	0.000	0.000
AS94_12755	*scn*		complement inhibitor	Y	0.994	0.000	0.000	1.320	0.000	0.000
AS94_12765			peptidoglycan hydrolase		0.700	0.000	0.000	0.537	0.001	0.004
AS94_12850			molecular chaperone GroES		0.653	0.000	0.000	0.925	0.000	0.000
AS94_12970	*ilvD*		dihydroxy-acid dehydratase	Y	1.657	0.000	0.000	1.727	0.000	0.000
AS94_12975	*ilvB*		acetolactate synthase	Y	1.006	0.000	0.000	1.070	0.000	0.000
AS94_12040			hypothetical protein		8.459	0.000	0.000	1.352	0.014	0.031
AS94_12055			autolysin		10.844	0.000	0.000	0.907	0.000	0.000
AS94_12070			tail protein		11.976	0.000	0.000	1.010	0.000	0.000
AS94_12075			cell wall hydrolase		12.788	0.000	0.000	1.057	0.000	0.000
AS94_12090			hypothetical protein		8.652	0.000	0.000	1.351	0.009	0.020
AS94_12095			hypothetical protein		12.193	0.000	0.000	0.901	0.000	0.000
AS94_12100			minor structural protein		12.567	0.000	0.000	0.806	0.000	0.000
AS94_12105		ϕSA169	peptidase		12.720	0.000	0.000	1.095	0.000	0.000
AS94_12110			phage tail protein		11.456	0.000	0.000	1.205	0.000	0.000
AS94_12115			membrane protein		13.916	0.000	0.000	0.843	0.000	0.000
AS94_12120			phi 11		9.902	0.000	0.000	1.235	0.001	0.002
AS94_12125			hypothetical protein		10.462	0.000	0.000	1.259	0.000	0.000
AS94_12130			tail protein		12.239	0.000	0.000	1.063	0.000	0.000
AS94_12135			phi 11		9.806	0.000	0.000	1.024	0.003	0.006
AS94_12140			hypothetical protein		9.238	0.000	0.000	1.349	0.002	0.004

AS94_12145		hypothetical protein	8.869	0.000	0.000	1.717	0.001	0.002
AS94_12150		phage head-tail adapter protein	10.259	0.000	0.000	1.569	0.000	0.000
AS94_12155		phi 11	8.348	0.000	0.000	1.495	0.010	0.022
AS94_12160		hypothetical protein	12.909	0.000	0.000	1.100	0.000	0.000
AS94_12165		phage capsid protein	13.409	0.000	0.000	1.440	0.000	0.000
AS94_12175		phage head morphogenesis protein	12.222	0.000	0.000	1.017	0.000	0.000
AS94_12180		phage portal protein	12.555	0.000	0.000	0.948	0.000	0.000
AS94_12185		hypothetical protein	12.014	0.000	0.000	1.121	0.000	0.000
AS94_12190		terminase	11.070	0.000	0.000	0.929	0.000	0.000
AS94_12345		BRO-like protein	12.337	0.000	0.000	0.458	0.006	0.015
AS94_12375		integrase	10.916	0.000	0.000	0.534	0.021	0.045
AS94_13265	mutual prophage	hypothetical protein	4.162	0.000	0.000	5.182	0.000	0.000
AS94_13290		hypothetical protein	8.740	0.000	0.000	8.643	0.000	0.000
AS94_13305		DNA replication protein DnaC	0.938	0.000	0.001	1.570	0.000	0.000
AS94_13310		replication protein	10.627	0.000	0.000	10.530	0.000	0.000
AS94_13315		hypothetical protein	10.070	0.000	0.000	9.973	0.000	0.000
AS94_13395		integrase	0.983	0.000	0.000	1.071	0.000	0.000
AS94_13490		peptidyl-prolyl cis-trans isomerase	2.346	0.000	0.000	2.758	0.000	0.000
AS94_13500		DNA double-strand break repair Rad50 ATPase	0.307	0.003	0.007	0.415	0.000	0.000
AS94_13575		amino acid ABC transporter ATP-binding protein	4.177	0.000	0.000	4.322	0.000	0.000
AS94_13580		glutamate ABC transporter permease	4.391	0.000	0.000	4.340	0.000	0.000
