# Supplementary material for: Transcriptome Analyses of Prophage in Mediating Persistent Methicillin-Resistant Staphylococcus aureus Endovascular Infection
Source: Genes (Basel). 2022 Aug 25;13(9):1527. doi: 10.3390/genes13091527 (PMC9498598; doi:10.3390/genes13091527)
Supplement: Supplementary file 1 [file genes-13-01527-s001.zip › Table S8.pdf]

Table S8. Down-regulated DEGs in both 300-169 vs. 301-188 and 300-169 vs. 301-188::ϕSA169

							**300-169 vs. 301-188**			**300-169 vs. 301-188::ϕSA169**		
**locus**	**gene**	**group**	**product**	***agr*-regulated (Y)**	***sar*A-regulated (Y)**	***sig*B-regulated (Y)**	**log_2(fold change)**	***p* value**	***p* adj**	**log_2(fold change)**	***p* value**	***p* adj**
AS94_00045			membrane protein				-0.688	0.001	0.002	-0.567	0.007	0.016
AS94_00055			dipeptidyl aminopeptidase				-0.628	0.000	0.000	-0.623	0.000	0.000
AS94_00195			multidrug MFS transporter				-0.319	0.021	0.044	-0.461	0.001	0.002
AS94_00205			hypothetical protein				-0.923	0.000	0.000	-0.894	0.000	0.000
AS94_00245			phosphotransferase				-0.858	0.000	0.000	-0.916	0.000	0.000
AS94_00250			tRNA (guanine-N(7)-)-methyltransferase				-0.494	0.000	0.000	-0.563	0.000	0.000
AS94_00255			hypothetical protein				-0.984	0.000	0.000	-0.895	0.000	0.000
AS94_00270			thioredoxin				-0.376	0.007	0.017	-0.406	0.004	0.010
AS94_00305			3-deoxy-7-phosphoheptulonate synthase				-1.464	0.000	0.000	-1.566	0.000	0.000
AS94_00370	*aroA*		3-phosphoglycerate dehydrogenase				-0.473	0.001	0.002	-0.480	0.001	0.002
AS94_00395	*serA*		hypothetical protein		Y		-0.414	0.017	0.035	-0.572	0.001	0.003
AS94_00435			universal stress protein UspA				-2.146	0.000	0.000	-1.876	0.000	0.000
AS94_00445			dipeptidase				-0.631	0.000	0.000	-0.632	0.000	0.000
AS94_00450			beta-lactamase				-1.246	0.000	0.000	-1.457	0.000	0.000
AS94_00460			universal stress protein UspA				-1.098	0.000	0.000	-1.219	0.000	0.000
AS94_00505	*pfkA*		6-phosphofructokinase				-0.997	0.000	0.000	-1.025	0.000	0.000
AS94_00510	*pyk*		pyruvate kinase				-0.667	0.000	0.000	-0.834	0.000	0.000
AS94_00525	*icd*		isocitrate dehydrogenase				-0.612	0.000	0.000	-0.533	0.000	0.000
AS94_00545	*polA*		DNA polymerase I				-0.519	0.000	0.000	-0.455	0.000	0.000
AS94_00550	*mutM*		formamidopyrimidine-DNA glycosylase				-0.486	0.000	0.000	-0.471	0.000	0.000
AS94_00555	*coaE*		dephospho-CoA kinase				-1.148	0.000	0.000	-1.236	0.000	0.000
AS94_00575			primosomal protein DnaI				-0.406	0.000	0.001	-0.388	0.001	0.002
AS94_00580	*thrS*		threonyl-tRNA synthase				-1.711	0.000	0.000	-1.991	0.000	0.000
AS94_00585			gamma-aminobutyrate permease				-1.052	0.000	0.000	-1.476	0.000	0.000
AS94_00645			uroporphyrinogen III synthase				-0.444	0.005	0.011	-0.371	0.019	0.041

AS94_00650	*hemB*	delta-aminolevulinic acid dehydratase		-0.273	0.022	0.044	-0.343	0.004	0.010
AS94_00655	*hemL*	glutamate-1-semialdehyde aminotransferase		-0.376	0.001	0.002	-0.492	0.000	0.000
AS94_00665		DNA-3-methyladenine glycosylase		-1.452	0.000	0.000	-1.350	0.000	0.000
AS94_00730	*rpmA*	50S ribosomal protein L27		-1.294	0.000	0.000	-1.359	0.000	0.000
AS94_00735		GTPase CgtA		-0.331	0.006	0.014	-0.346	0.004	0.011
AS94_00785	*relA*	GTP pyrophosphokinase	Y	-0.367	0.000	0.001	-0.241	0.022	0.047
AS94_00795		cell wall amidase		-0.459	0.000	0.001	-0.446	0.001	0.002
AS94_00800		hypothetical protein		-0.369	0.014	0.029	-0.383	0.011	0.024
AS94_00810	*aspS*	aspartyl-tRNA synthase		-0.666	0.000	0.000	-0.611	0.000	0.000
AS94_00840		hypothetical protein		-0.698	0.000	0.000	-0.828	0.000	0.000
AS94_00860		hypothetical protein		-0.429	0.000	0.001	-0.396	0.001	0.002
AS94_00870		hypothetical protein		-0.524	0.001	0.002	-0.541	0.001	0.002
AS94_00875		Holliday junction resolvase		-0.517	0.000	0.000	-0.446	0.000	0.001
AS94_00880		hypothetical protein		-0.394	0.003	0.006	-0.433	0.001	0.003
AS94_00905		transcription elongation factor GreA		-0.418	0.001	0.003	-0.305	0.017	0.036
AS94_01005	*comE*	deoxycytidylate deaminase		-0.636	0.000	0.001	-0.694	0.000	0.000
AS94_01185		superoxide dismutase		-0.792	0.000	0.000	-0.779	0.000	0.000
AS94_01260		shikimate kinase		-0.601	0.000	0.001	-0.493	0.003	0.008
AS94_01265	*gcvT*	glycine cleavage system protein T		-0.624	0.000	0.000	-0.518	0.000	0.001
AS94_01270		glycine dehydrogenase subunit 1		-0.769	0.000	0.000	-0.706	0.000	0.000
AS94_01275		glycine dehydrogenase subunit 2		-1.048	0.000	0.000	-1.029	0.000	0.000
AS94_01370		2-oxoglutarate dehydrogenase E2		-0.549	0.000	0.000	-0.639	0.000	0.000
AS94_01405		transcriptional regulator		-1.046	0.000	0.000	-0.834	0.000	0.000
AS94_01410	*malA*	trehalose-6-phosphate hydrolase		-1.817	0.000	0.000	-1.788	0.000	0.000
AS94_01430	*proC*	pyrroline-5-carboxylate reductase		-0.782	0.000	0.000	-0.677	0.000	0.000
AS94_01460		tyrosine recombinase XerD		-0.925	0.000	0.000	-0.800	0.000	0.000
AS94_01490	*srrB*	sensor histidine kinase		-0.390	0.000	0.001	-0.314	0.003	0.006
AS94_01560		glycerol-3-phosphate dehydrogenase		-0.516	0.000	0.000	-0.621	0.000	0.000

AS94_01565		DNA-binding protein	-0.800	0.000	0.000	-0.780	0.000	0.000
AS94_01630		hypothetical protein	-0.556	0.006	0.013	-0.463	0.022	0.046
AS94_01665	*nth*	endonuclease III	-0.449	0.013	0.028	-0.649	0.000	0.001
AS94_01700		hypothetical protein	-0.394	0.000	0.001	-0.461	0.000	0.000
AS94_01735	*ilvA*	threonine dehydratase	-0.607	0.000	0.000	-0.402	0.011	0.025
AS94_01745		quinolone resistance protein NorB	-0.667	0.000	0.000	-0.495	0.001	0.004
AS94_01750		matrix-binding protein	-0.438	0.000	0.000	-0.381	0.000	0.001
AS94_01780		hypothetical protein	-0.493	0.000	0.001	-0.648	0.000	0.000
AS94_01795		hypothetical protein	-0.462	0.000	0.000	-0.444	0.000	0.000
AS94_01800		methionine sulfoxide reductase A	-0.421	0.000	0.000	-0.371	0.001	0.002
AS94_01805		methionine sulfoxide reductase B	-0.355	0.005	0.012	-0.466	0.000	0.001
AS94_01810	*crr*	PTS glucose transporter subunit IIA	-0.295	0.012	0.025	-0.432	0.000	0.001
AS94_01920		hypothetical protein	-1.003	0.000	0.000	-0.943	0.000	0.000
AS94_01935		protease	-0.737	0.000	0.000	-0.909	0.000	0.000
AS94_02100		aminopeptidase	-0.884	0.000	0.000	-0.875	0.000	0.000
AS94_02105		prephenate dehydrogenase	-0.747	0.000	0.000	-0.810	0.000	0.000
AS94_02110		DNA repair protein MucB	-1.701	0.000	0.000	-1.518	0.000	0.000
AS94_02115		4-oxalocrotonate tautomerase	-1.777	0.000	0.000	-1.891	0.000	0.000
AS94_02125		methionine sulfoxide reductase A	-0.532	0.000	0.001	-0.396	0.007	0.017
AS94_02145		transcriptional regulator	-0.589	0.005	0.012	-0.709	0.001	0.002
AS94_02165	*plsY*	glycerol-3-phosphate acyltransferase	-0.456	0.000	0.001	-0.377	0.002	0.006
AS94_02230		XRE family transcriptional regulator	-1.344	0.000	0.000	-1.470	0.000	0.000
AS94_02255	*rpmG*	50S ribosomal protein L33	-0.526	0.002	0.004	-0.422	0.012	0.027
AS94_02270		hypothetical protein	-0.544	0.000	0.001	-0.378	0.009	0.022
AS94_02530	*miaA*	tRNA delta(2)-isopentenylpyrophosphate transferase	-0.440	0.013	0.028	-0.411	0.020	0.044
AS94_02535		lysophospholipase	-0.980	0.000	0.000	-0.821	0.000	0.000
AS94_02540		glycerol-3-phosphate dehydrogenase	-0.736	0.000	0.000	-0.747	0.000	0.000
AS94_02590		2-oxoacid ferredoxin oxidoreductase subunit beta	-0.782	0.000	0.000	-1.289	0.000	0.000

AS94_02595		2-oxoglutarate ferredoxin oxidoreductase subunitalpha			-0.948	0.000	0.000	-1.245	0.000	0.000
AS94_02615	*recA*	protein RecA			-0.281	0.006	0.013	-0.316	0.002	0.005
AS94_02660		cell division protein FtsK			-0.277	0.014	0.030	-0.289	0.010	0.024
AS94_02665	*rnjB*	ribonuclease J			-0.452	0.000	0.000	-0.474	0.000	0.000
AS94_02690		ribosome-binding factor A			-0.467	0.001	0.002	-0.560	0.000	0.000
AS94_02720	*polC*	DNA polymerase III subunit alpha			-0.333	0.002	0.004	-0.432	0.000	0.000
AS94_02865	*ffh*	signal recognition particle protein Srp54			-0.764	0.000	0.000	-0.786	0.000	0.000
AS94_02930	*rpmB*	50S ribosomal protein L28			-1.592	0.000	0.000	-1.954	0.000	0.000
AS94_02990	*priA*	primosome assembly protein PriA			-0.682	0.000	0.000	-0.645	0.000	0.000
AS94_02995	*coaBC*	phosphopantothenoylcysteine decarboxylase			-0.718	0.000	0.000	-0.662	0.000	0.000
AS94_03000	*rpoZ*	DNA-directed RNA polymerase subunit omega			-0.981	0.000	0.001	-1.050	0.000	0.001
AS94_03005	*gmk*	guanylate kinase			-0.391	0.002	0.005	-0.338	0.007	0.017
AS94_03075		glyoxalase			-1.094	0.000	0.000	-1.127	0.000	0.000
AS94_03080	*ileS*	isoleucyl-tRNA synthetase			-0.878	0.000	0.000	-0.993	0.000	0.000
AS94_03170		5'-nucleotidase			-0.503	0.000	0.000	-0.493	0.000	0.000
AS94_03205		hypothetical protein			-0.894	0.000	0.000	-0.951	0.000	0.000
AS94_03210	*arcC*	carbamate kinase	Y		-2.248	0.000	0.000	-2.256	0.000	0.000
AS94_03215	*argF*	ornithine carbamoyltransferase	Y	Y	-0.905	0.000	0.000	-0.686	0.000	0.000
AS94_03290		phosphoesterase			-1.105	0.000	0.000	-1.167	0.000	0.000
AS94_03295	*rdgB*	deoxyribonucleotide triphosphate pyrophosphatase			-1.087	0.000	0.000	-1.136	0.000	0.000
AS94_03300	*murI*	glutamate racemase			-1.035	0.000	0.000	-1.082	0.000	0.000
AS94_03325		thioredoxin			-0.716	0.000	0.000	-0.610	0.000	0.000
AS94_03355	*pheT*	phenylalanyl-tRNA synthase subunit beta			-0.647	0.000	0.000	-0.694	0.000	0.000
AS94_03425	*coaD*	phosphopantetheine adenylyltransferase			-0.633	0.000	0.001	-0.611	0.001	0.002
AS94_03505		inositol monophosphatase			-0.694	0.000	0.000	-0.780	0.000	0.000
AS94_03510		hypothetical protein			-0.787	0.000	0.000	-0.862	0.000	0.000
AS94_03515		manganese transporter			-0.467	0.000	0.000	-0.496	0.000	0.000

AS94_03590		hypothetical protein			-0.498	0.001	0.002	-0.339	0.020	0.044
AS94_03635		SAM-dependent methyltransferase			-1.007	0.000	0.000	-0.999	0.000	0.000
AS94_03650		thiamine ABC transporter permease			-0.662	0.002	0.006	-0.785	0.000	0.001
AS94_03655		ABC transporter ATP-binding protein			-0.672	0.000	0.000	-0.735	0.000	0.000
AS94_03660		cobalt ABC transporter permease			-1.017	0.000	0.000	-1.253	0.000	0.000
AS94_03735	*qoxA*	quinol oxidase subunit 2			-0.806	0.000	0.000	-0.799	0.000	0.000
AS94_03740	*qoxB*	quinol oxidase subunit 1			-1.580	0.000	0.000	-1.679	0.000	0.000
AS94_03745	*qoxC*	cytochrome O ubiquinol oxidase			-1.927	0.000	0.000	-2.104	0.000	0.000
AS94_03750	*qoxD*	quinol oxidase subunit 4			-2.152	0.000	0.000	-2.221	0.000	0.000
AS94_03760		hypothetical protein			-0.324	0.012	0.027	-0.488	0.000	0.001
AS94_03775		mannosyl-glycoprotein endo-beta-N-acetylglucosamidase			-0.453	0.000	0.000	-0.465	0.000	0.000
AS94_03780		MarR family transcriptional regulator			-0.911	0.000	0.001	-0.851	0.001	0.003
AS94_03795	*sspA*	glutamyl endopeptidase	Y	Y	-2.628	0.000	0.000	-1.963	0.000	0.000
AS94_03800		cysteine protease			-2.378	0.000	0.000	-1.563	0.000	0.000
AS94_03810	*menB*	dihydroxynaphthoic acid synthetase			-1.448	0.000	0.000	-1.479	0.000	0.000
AS94_03815	*menH*	2-succinyl-6-hydroxy-2_ 4-cyclohexadiene-1-carboxylate synthase			-1.214	0.000	0.000	-1.194	0.000	0.000
AS94_03820	*menD*	2-succinyl-5-enolpyruvyl-6-hydroxy-3-cyclohexene-1-carboxylate synthase			-0.629	0.000	0.000	-0.691	0.000	0.000
AS94_03835		acetyltransferase			-0.811	0.000	0.000	-0.783	0.000	0.000
AS94_03870		bacteriocin ABC transporter ATP-binding protein			-0.675	0.017	0.035	-1.139	0.000	0.000
AS94_03880		bacteriocin-associated integral membrane protein			-0.751	0.000	0.001	-0.742	0.000	0.001
AS94_03900		IDEAL domain protein			-0.938	0.000	0.000	-1.069	0.000	0.000
AS94_03940		peptide chain release factor 1			-0.321	0.003	0.008	-0.332	0.002	0.006
AS94_03995		magnesium transporter MgtE			-0.344	0.014	0.029	-0.459	0.001	0.003
AS94_04005		inorganic polyphosphate/ATP-NAD kinase			-0.574	0.000	0.000	-0.707	0.000	0.000
AS94_04010		GTP pyrophosphokinase			-0.890	0.000	0.000	-0.940	0.000	0.000
AS94_04045		competence negative regulator MecA			-0.638	0.000	0.000	-0.782	0.000	0.000
AS94_04130		MAP domain protein			-0.945	0.000	0.000	-0.687	0.000	0.000

AS94_04175		hypothetical protein	-0.811	0.000	0.000	-0.821	0.000	0.000
AS94_04180		ATP-dependent DNA helicase subunit A	-0.772	0.000	0.000	-0.776	0.000	0.000
AS94_04185		ATP-dependent DNA helicase subunit B	-0.815	0.000	0.000	-0.820	0.000	0.000
AS94_04215	*pgi*	glucose-6-phosphate isomerase	-0.374	0.002	0.006	-0.359	0.004	0.009
AS94_04230		glycerophosphoryl diester phosphodiesterase	-0.606	0.000	0.001	-0.461	0.006	0.014
AS94_04245		NADH-dependent flavin oxidoreductase	-0.844	0.000	0.000	-0.989	0.000	0.000
AS94_04285		cation:proton antiporter	-0.574	0.015	0.032	-0.918	0.000	0.000
AS94_04290		cation:proton antiporter	-2.171	0.000	0.000	-2.248	0.000	0.000
AS94_04295		cation:proton antiporter	-0.808	0.002	0.005	-1.343	0.000	0.000
AS94_04300		hypothetical protein	-1.823	0.000	0.000	-1.642	0.000	0.000
AS94_04305		thioesterase	-0.775	0.000	0.000	-0.828	0.000	0.000
AS94_04310		sodium:proton antiporter	-0.610	0.001	0.001	-0.666	0.000	0.001
AS94_04325		hypothetical protein	-0.429	0.004	0.010	-0.530	0.000	0.001
AS94_04345		nitrogen-fixing protein NifU	-0.560	0.001	0.002	-0.444	0.007	0.017
AS94_04375	*gyaR*	2-ketogluconate reductase	-0.394	0.009	0.021	-0.674	0.000	0.000
AS94_04505		arsenate reductase	-0.914	0.000	0.000	-0.880	0.000	0.000
AS94_04510		thioredoxin	-0.347	0.008	0.018	-0.428	0.001	0.003
AS94_04560		membrane protein	-2.763	0.000	0.000	-2.984	0.000	0.000
AS94_04580		cold-shock protein	-0.742	0.000	0.000	-0.527	0.000	0.000
AS94_04585		thermonuclease	-1.632	0.000	0.000	-1.211	0.000	0.000
AS94_04630	*entK*	enterotoxin I	-1.137	0.000	0.000	-0.795	0.000	0.000
AS94_04645		hypothetical protein	-0.575	0.000	0.000	-0.548	0.000	0.000
AS94_04660		transposase	-0.990	0.000	0.000	-0.890	0.000	0.000
AS94_04665		transposase	-0.790	0.001	0.002	-0.806	0.001	0.002
AS94_04700		deacetylase	-0.329	0.012	0.026	-0.546	0.000	0.000
AS94_04710	*nagB*	glucosamine-6-phosphate deaminase	-0.712	0.000	0.000	-0.606	0.000	0.001
AS94_04720	*hxlB*	6-phospho 3-hexuloisomerase	-0.333	0.008	0.017	-0.395	0.001	0.004
AS94_04760		pyridoxal kinase	-0.486	0.000	0.000	-0.711	0.000	0.000

AS94_04890	*adh*	acetaldehyde reductase	-0.374	0.016	0.034	-0.664	0.000	0.000
AS94_04970	*cysK*	cysteine synthase	-0.320	0.005	0.011	-0.317	0.005	0.012
AS94_04975		heat shock protein Hsp33	-0.609	0.000	0.000	-0.765	0.000	0.000
AS94_04995		hypothetical protein	-0.584	0.000	0.000	-0.507	0.000	0.000
AS94_05010		nucleotide pyrophosphohydrolase	-0.656	0.000	0.000	-0.789	0.000	0.000
AS94_05035	*prs*	ribose-phosphate pyrophosphokinase	-0.407	0.000	0.000	-0.428	0.000	0.000
AS94_05085	*metG*	methionyl-tRNA synthetase	-0.427	0.000	0.000	-0.471	0.000	0.000
AS94_05105		DNA replication protein YabA	-0.878	0.000	0.000	-0.852	0.000	0.000
AS94_05110		signal peptidase II	-0.474	0.000	0.001	-0.417	0.001	0.003
AS94_05120		hypothetical protein	-0.758	0.000	0.000	-0.742	0.000	0.000
AS94_05125	*tmk*	thymidylate kinase	-0.806	0.000	0.000	-0.973	0.000	0.000
AS94_05130		lysine decarboxylase	-0.873	0.000	0.000	-0.729	0.000	0.000
AS94_05155		hypothetical protein	-0.494	0.001	0.002	-0.915	0.000	0.000
AS94_05290		integrase	-0.360	0.019	0.039	-0.353	0.021	0.045
AS94_05410		transposase	-2.153	0.000	0.000	-2.258	0.000	0.000
AS94_05425		hypothetical protein	-0.668	0.002	0.006	-0.577	0.009	0.021
AS94_05440		NA	-0.518	0.015	0.032	-0.566	0.008	0.018
AS94_05445		chromosome partitioning protein ParA	-0.481	0.000	0.000	-0.550	0.000	0.000
AS94_05500		iron citrate ABC transporter substrate-binding protein	-1.581	0.000	0.000	-1.250	0.000	0.000
AS94_05555		alcohol dehydrogenase	-0.797	0.000	0.000	-0.802	0.000	0.000
AS94_05560		alcohol dehydrogenase	-0.755	0.000	0.000	-0.805	0.000	0.000
AS94_05570	*lacG*	6-phospho-beta-galactosidase	-2.586	0.000	0.000	-2.188	0.000	0.000
AS94_05575	*lacE*	PTS lactose transporter subunit IIBC	-2.334	0.000	0.000	-1.592	0.000	0.000
AS94_05580	*lacF*	PTS lactose transporter subunit IIA	-3.043	0.000	0.000	-1.933	0.000	0.000
AS94_05585	*lacD*	tagatose-bisphosphate aldolase	-2.569	0.000	0.000	-1.762	0.000	0.000
AS94_05590	*lacC*	tagatose-6-phosphate kinase	-2.374	0.000	0.000	-1.519	0.000	0.000
AS94_05595	*lacB*	galactose-6-phosphate isomerase	-2.300	0.000	0.000	-1.210	0.000	0.000
AS94_05600	*lacA*	galactose-6-phosphate isomerase	-2.599	0.000	0.000	-1.840	0.000	0.000

AS94_05605		DeoR family transcriptional regulator		-0.556	0.002	0.004	-0.556	0.002	0.004
AS94_05615		2_5-diketo-D-gluconic acid reductase		-0.809	0.000	0.000	-0.966	0.000	0.000
AS94_05655	*budA*	alpha-acetolactate decarboxylase	Y	-2.037	0.000	0.000	-2.048	0.000	0.000
AS94_05660	*alsS*	acetolactate synthase		-2.320	0.000	0.000	-2.238	0.000	0.000
AS94_05705		cobalt ABC transporter ATP-binding protein		-0.473	0.005	0.013	-0.542	0.001	0.004
AS94_05870		GNAT family acetyltransferase		-0.873	0.000	0.000	-0.834	0.000	0.000
AS94_05880		malonate transporter		-0.768	0.000	0.000	-0.922	0.000	0.000
AS94_05890		membrane protein		-0.495	0.001	0.003	-0.586	0.000	0.000
AS94_05920		MarR family transcriptional regulator		-0.444	0.013	0.028	-0.607	0.001	0.002
AS94_05930	*moaA*	molybdenum cofactor biosynthesis protein A		-0.594	0.000	0.000	-0.841	0.000	0.000
AS94_05935		molybdopterin-guanine dinucleotide biosynthesis protein A		-0.681	0.000	0.000	-0.839	0.000	0.000
AS94_05945	*moaE*	molybdopterin synthase subunit 2		-1.051	0.000	0.000	-1.042	0.000	0.000
AS94_05950		molybdopterin-guanine dinucleotide biosynthesis protein B		-0.667	0.001	0.003	-0.747	0.000	0.001
AS94_05955	*moeA*	molybdopterin molybdenumtransferase		-1.838	0.000	0.000	-1.877	0.000	0.000
AS94_05960	*moaC*	molybdenum cofactor biosynthesis protein C		-1.334	0.000	0.000	-1.289	0.000	0.000
AS94_05965	*moaB*	molybdenum cofactor biosynthesis protein B		-1.780	0.000	0.000	-1.775	0.000	0.000
AS94_05970	*moeZ/MoeB*	molybdopterin biosynthesis protein MoeB		-1.371	0.000	0.000	-1.200	0.000	0.000
AS94_05975		molybdenum ABC transporter ATP-binding protein		-1.366	0.000	0.000	-1.476	0.000	0.000
AS94_05980	*modB*	molybdenum ABC transporter permease		-1.152	0.000	0.000	-1.250	0.000	0.000
AS94_05985	*modA*	molybdenum ABC transporter substrate-binding protein		-0.692	0.000	0.000	-0.388	0.015	0.034
AS94_05990		formate dehydrogenase subunit D		-0.503	0.001	0.003	-0.884	0.000	0.000
AS94_06010		ferrichrome ABC transporter substrate-binding protein		-0.679	0.000	0.000	-0.714	0.000	0.000
AS94_06015		acyl-CoA dehydrogenase		-0.545	0.001	0.001	-0.473	0.003	0.007
AS94_06020		urea transporter		-1.505	0.000	0.000	-1.519	0.000	0.000
AS94_06025	*ureA*	urease subunit gamma	Y	-1.562	0.005	0.011	-1.313	0.018	0.039
AS94_06030	*ureB*	urease subunit beta	Y	-1.691	0.000	0.001	-1.604	0.001	0.001

AS94_06035	*ureC*	urease subunit alpha	Y	-1.091	0.000	0.000	-1.104	0.000	0.000
AS94_06040		urease accessory protein UreE		-1.128	0.000	0.000	-0.692	0.022	0.047
AS94_06045		urease accessory protein UreF		-1.047	0.000	0.000	-1.080	0.000	0.000
AS94_06050		urease accessory protein UreG		-1.179	0.000	0.000	-1.076	0.000	0.000
AS94_06055		urease accessory protein UreD		-1.412	0.000	0.000	-1.468	0.000	0.000
AS94_06060		MarR family transcriptional regulator		-0.661	0.000	0.000	-0.752	0.000	0.000
AS94_06065		hypothetical protein		-0.937	0.000	0.000	-0.700	0.000	0.000
AS94_06070		transcriptional regulator		-0.659	0.018	0.038	-0.654	0.020	0.042
AS94_06100		hypothetical protein		-0.665	0.000	0.000	-1.172	0.000	0.000
AS94_06110		2-hydroxyacid dehydrogenase		-1.462	0.000	0.000	-1.415	0.000	0.000
AS94_06115		hypothetical protein		-0.734	0.000	0.000	-0.777	0.000	0.000
AS94_06130		hypothetical protein		-0.935	0.000	0.000	-1.438	0.000	0.000
AS94_06135		oxidoreductase		-0.524	0.000	0.000	-0.751	0.000	0.000
AS94_06155		DeoR family transcriptional regulator		-0.879	0.000	0.000	-0.848	0.000	0.000
AS94_06195		HAD family hydrolase		-0.592	0.005	0.012	-0.655	0.002	0.005
AS94_06210		PTS alpha-glucoside transporter subunit IIBC		-2.524	0.000	0.000	-2.416	0.000	0.000
AS94_06225		sodium:proton antiporter		-0.460	0.003	0.008	-0.555	0.000	0.001
AS94_06235		oxidoreductase		-0.298	0.012	0.026	-0.582	0.000	0.000
AS94_06265		lysostaphin resistance protein A		-0.451	0.001	0.002	-0.943	0.000	0.000
AS94_06270	*rpiA*	ribose 5-phosphate isomerase		-0.857	0.000	0.000	-1.125	0.000	0.000
AS94_06275		molybdenum cofactor biosysynthesis protein		-0.550	0.002	0.006	-0.831	0.000	0.000
AS94_06280		aldose 1-epimerase		-0.555	0.000	0.001	-0.382	0.015	0.033
AS94_06285		membrane protein		-0.764	0.001	0.001	-0.525	0.018	0.040
AS94_06305		3-methyladenine DNA glycosylase		-1.074	0.000	0.000	-1.077	0.000	0.000
AS94_06315	*fni*	isopentenyl pyrophosphate isomerase		-0.523	0.000	0.000	-0.522	0.000	0.000
AS94_06380		hemin ABC transporter ATP-binding protein		-2.343	0.000	0.000	-2.303	0.000	0.000
AS94_06385		hemin ABC transporter permease		-2.331	0.000	0.000	-2.441	0.000	0.000
AS94_06390		heme transporter CcmC		-0.866	0.001	0.003	-0.871	0.001	0.003

AS94_06395		sensor histidine kinase		-1.154	0.000	0.000	-1.345	0.000	0.000
AS94_06420		antibiotic ABC transporter permease		-0.928	0.003	0.007	-1.124	0.000	0.001
AS94_06445		acetyltransferase		-0.846	0.001	0.002	-0.746	0.003	0.007
AS94_06460		GNAT family acetyltransferase		-0.981	0.000	0.000	-1.040	0.000	0.000
AS94_06465		ferredoxin--NADP reductase		-1.499	0.000	0.000	-1.362	0.000	0.000
AS94_06490		PTS sucrose transporter subunit IIBC		-2.511	0.000	0.000	-2.418	0.000	0.000
AS94_06550	*nreC*	LuxR family transcriptional regulator	Y	-0.772	0.000	0.000	-0.798	0.000	0.000
AS94_06555	*nreB*	sensor histidine kinase	Y	-0.758	0.000	0.000	-0.531	0.000	0.000
AS94_06560		nreA protein		-0.869	0.000	0.000	-0.653	0.000	0.000
AS94_06565	*narI*	nitrate reductase subunit gamma	Y	-0.879	0.000	0.000	-0.839	0.000	0.000
AS94_06570	*narJ*	nitrate reductase sununit delta	Y	-1.490	0.000	0.000	-1.421	0.000	0.000
AS94_06575	*narH*	nitrate reductase	Y	-0.998	0.000	0.000	-0.926	0.000	0.000
AS94_06580		nitrate reductase		-1.111	0.000	0.000	-0.997	0.000	0.000
AS94_06585	*cobA*	uroporphyrinogen III methyltransferase		-1.230	0.000	0.000	-1.445	0.000	0.000
AS94_06590	*nirD*	nitrite reductase NAD(P)H small subunit		-0.916	0.000	0.001	-1.350	0.000	0.000
AS94_06595	*nirB*	nitrite reductase		-1.216	0.000	0.000	-1.209	0.000	0.000
AS94_06600		cobalamin biosynthesis protein CbiX		-1.105	0.000	0.000	-0.901	0.000	0.000
AS94_06625	*yodA*	zinc ABC transporter substrate-binding protein		-0.421	0.007	0.016	-0.361	0.021	0.045
AS94_06630		protein-disulfide isomerase		-0.766	0.000	0.000	-0.496	0.011	0.025
AS94_06665		hypothetical protein		-1.160	0.001	0.002	-1.387	0.000	0.000
AS94_06745		hypothetical protein		-1.320	0.000	0.001	-1.690	0.000	0.000
AS94_06765		bicyclomycin transporter TcaB		-0.428	0.001	0.003	-0.481	0.000	0.001
AS94_06775		hypothetical protein		-0.705	0.000	0.000	-0.616	0.000	0.000
AS94_06795		amino acid permease		-1.054	0.000	0.000	-1.036	0.000	0.000
AS94_06805		epimerase		-0.584	0.000	0.000	-0.751	0.000	0.000
AS94_06810		2-dehydropantoate 2-reductase		-1.502	0.000	0.000	-1.571	0.000	0.000
AS94_06815		quinolone resistance protein NorB		-0.820	0.000	0.000	-1.012	0.000	0.000
AS94_06820		amino acid ABC transporter permease		-1.528	0.000	0.000	-1.689	0.000	0.000

AS94_06825	*opuCC*	glycine/betaine ABC transporter substrate-binding protein			-1.462	0.000	0.000	-1.528	0.000	0.000
AS94_06830		choline ABC transporter permease			-0.926	0.000	0.000	-0.835	0.000	0.000
AS94_06835	*proV*	glycine/betaine ABC transporter ATP-binding protein			-1.096	0.000	0.000	-0.801	0.000	0.000
AS94_06840		hypothetical protein			-0.818	0.000	0.000	-0.705	0.000	0.000
AS94_06845		amino acid:proton symporter			-0.696	0.000	0.000	-0.948	0.000	0.000
AS94_06860		membrane protein			-0.668	0.000	0.000	-0.989	0.000	0.000
AS94_06870		peptidase M28			-1.188	0.000	0.000	-1.211	0.000	0.000
AS94_06875		hypothetical protein			-0.995	0.000	0.000	-0.858	0.000	0.000
AS94_06985		hypothetical protein			-1.655	0.000	0.000	-1.486	0.000	0.000
AS94_06990		hypothetical protein			-0.980	0.000	0.000	-1.159	0.000	0.000
AS94_06995		hypothetical protein			-1.651	0.000	0.000	-1.505	0.000	0.000
AS94_07000		hypothetical protein			-0.944	0.000	0.000	-0.845	0.000	0.000
AS94_07010		hypothetical protein			-0.929	0.000	0.000	-0.764	0.000	0.000
AS94_07050	*galU*	UTP--glucose-1-phosphate uridylyltransferase			-0.864	0.000	0.000	-0.790	0.000	0.000
AS94_07055	*fnbA*	fibronectin-binding protein A	Y		-0.675	0.000	0.000	-0.561	0.000	0.001
AS94_07065		gluconate permease			-1.314	0.000	0.000	-1.153	0.000	0.000
AS94_07070	*gntK*	gluconokinase		Y	-0.704	0.000	0.000	-0.317	0.005	0.012
AS94_07080		MerR family transcriptional regulator			-1.248	0.000	0.000	-1.302	0.000	0.000
AS94_07120	*fbp*	fructose-1_6-bisphosphatase			-0.684	0.000	0.000	-0.619	0.000	0.000
AS94_07130		carboxylesterase			-1.216	0.000	0.000	-1.161	0.000	0.000
AS94_07135		glyoxalase			-0.813	0.000	0.000	-0.723	0.000	0.001
AS94_07140		MarR family transcriptional regulator			-1.298	0.000	0.000	-1.147	0.000	0.000
AS94_07145		acetyltransferase			-0.474	0.006	0.015	-0.394	0.024	0.050
AS94_07150		glyoxalase			-0.476	0.003	0.008	-0.546	0.001	0.002
AS94_07155		NAD(P)H nitroreductase			-0.572	0.000	0.000	-0.788	0.000	0.000
AS94_07165		HAD family hydrolase			-0.690	0.000	0.001	-0.712	0.000	0.001
AS94_07215		esterase			-1.153	0.000	0.000	-1.431	0.000	0.000
AS94_07225		acyl-CoA thioester hydrolase			-1.124	0.000	0.000	-1.027	0.000	0.000

AS94_07230	*glcB*	PTS glucose transporter subunit IIABC		-1.180	0.000	0.000	-1.206	0.000	0.000
AS94_07235		pyruvate oxidase		-0.789	0.000	0.000	-1.038	0.000	0.000
AS94_07240		holin		-0.434	0.000	0.001	-0.753	0.000	0.000
AS94_07245		holin		-2.360	0.000	0.000	-2.964	0.000	0.000
AS94_07265		hydroxymethylglutaryl-CoA reductase		-0.342	0.008	0.018	-0.320	0.013	0.029
AS94_07275		methylated DNA-protein cysteine S-methyltransferase		-0.767	0.000	0.000	-0.863	0.000	0.000
AS94_07395		TetR family transcriptional regulator		-0.747	0.001	0.002	-0.849	0.000	0.000
AS94_07475		fructosamine-3-kinase		-0.562	0.000	0.000	-0.860	0.000	0.000
AS94_07510	*panD*	aspartate decarboxylase		-0.527	0.001	0.003	-0.460	0.004	0.011
AS94_07515	*panC*	pantoate--beta-alanine ligase		-0.395	0.003	0.006	-0.400	0.002	0.006
AS94_07520	*panB*	3-methyl-2-oxobutanoate hydroxymethyltransferase		-0.444	0.005	0.012	-0.432	0.006	0.015
AS94_07530	*budA*	alpha-acetolactate decarboxylase	Y	-0.837	0.000	0.000	-0.857	0.000	0.000
AS94_07560	*mqo*	malate:quinone oxidoreductase		-0.437	0.000	0.000	-0.522	0.000	0.000
AS94_07570		acyl--CoA ligase		-0.676	0.000	0.000	-0.782	0.000	0.000
AS94_07590	*betB*	betaine-aldehyde dehydrogenase	Y	-0.445	0.003	0.008	-0.668	0.000	0.000
AS94_07595		hypothetical protein		-0.387	0.019	0.040	-0.542	0.001	0.003
AS94_07600		choline transporter BetT		-0.433	0.008	0.017	-0.655	0.000	0.000
AS94_07605		ribonucleoside-triphosphate reductase activatingprotein		-0.633	0.000	0.000	-0.894	0.000	0.000
AS94_07620		precorrin-2 dehydrogenase		-0.640	0.000	0.000	-0.828	0.000	0.000
AS94_07625		sulfite reductase subunit alpha		-0.583	0.000	0.000	-0.734	0.000	0.000
AS94_07635		peptide ABC transporter permease		-0.610	0.000	0.001	-0.667	0.000	0.000
AS94_07675		tributyrin esterase		-0.817	0.000	0.000	-0.758	0.000	0.000
AS94_07685		hypothetical protein		-0.997	0.001	0.002	-0.966	0.001	0.003
AS94_07690		type I restriction endonuclease subunit S		-0.999	0.000	0.000	-0.853	0.000	0.000
AS94_07700		RNA helicase		-0.925	0.000	0.000	-0.913	0.000	0.000
AS94_07710		hypothetical protein		-0.768	0.000	0.000	-0.729	0.000	0.000
AS94_07715		hypothetical protein		-1.180	0.000	0.000	-1.049	0.000	0.000
AS94_07720		transposase		-0.856	0.000	0.000	-0.737	0.000	0.000

AS94_07800		Vitamin B12 ABC transporter substrate-binding protein		-0.665	0.000	0.000	-0.516	0.003	0.007
AS94_07810		HAD family hydrolase		-0.757	0.000	0.001	-0.742	0.001	0.002
AS94_07815		alpha/beta hydrolase		-0.934	0.000	0.000	-1.002	0.000	0.000
AS94_07820		hypothetical protein		-1.127	0.000	0.000	-1.548	0.000	0.000
AS94_07925		N-acetylmannosaminyltransferase		-0.491	0.004	0.009	-0.587	0.001	0.002
AS94_07930	*tagH*	teichoic acid ABC transporter ATP-binding protein		-0.445	0.000	0.001	-0.565	0.000	0.000
AS94_07965		nucleoside permease		-0.894	0.000	0.000	-0.908	0.000	0.000
AS94_07975		iron-dicitrate ABC transporter ATP-binding protein		-1.143	0.000	0.000	-0.977	0.000	0.000
AS94_07980		ferrichrome ABC transporter permease		-1.020	0.000	0.000	-0.894	0.000	0.000
AS94_07985		iron ABC transporter permease		-1.089	0.000	0.000	-0.956	0.000	0.000
AS94_07995	*dhaL*	dihydroxyacetone kinase subunit L		-0.684	0.000	0.000	-0.536	0.000	0.001
AS94_08000	*dhaM*	PTS mannnose transporter subunit IIA		-1.387	0.000	0.000	-1.151	0.000	0.000
AS94_08005		hypothetical protein		-0.456	0.000	0.001	-0.573	0.000	0.000
AS94_08025		acetyltransferase		-0.866	0.000	0.000	-0.890	0.000	0.000
AS94_08030		3-beta hydroxysteroid dehydrogenase		-0.466	0.001	0.004	-0.508	0.001	0.002
AS94_08050		bacitracin ABC transporter permease		-0.704	0.000	0.000	-0.764	0.000	0.000
AS94_08065		peptidase M23B		-0.353	0.003	0.007	-0.580	0.000	0.000
AS94_08070		inhibitor of apoptosis-promoting Bax1		-0.713	0.000	0.000	-0.631	0.000	0.000
AS94_08075		AraC family transcriptional regulator		-0.876	0.000	0.000	-0.966	0.000	0.000
AS94_08080		MarR family transcriptional regulator		-3.088	0.000	0.000	-2.695	0.000	0.000
AS94_08095		hypothetical protein		-0.639	0.000	0.000	-0.547	0.000	0.000
AS94_08100		LysR family transcriptional regulator		-0.574	0.000	0.000	-0.561	0.000	0.000
AS94_08105		sugar MFS transporter		-0.567	0.000	0.000	-0.643	0.000	0.000
AS94_08170		MarR family transcriptional regulator		-0.265	0.008	0.019	-0.281	0.005	0.013
AS94_08195		deoxyribodipyrimidine photo-lyase		-0.283	0.011	0.024	-0.334	0.003	0.007
AS94_08220		hypothetical protein		-0.499	0.007	0.017	-0.999	0.000	0.000
AS94_08225		YbaK/EbsC protein		-1.377	0.000	0.000	-1.240	0.000	0.000
AS94_08240	*fruA*	PTS fructose transporter subunit IIC	Y	-0.562	0.000	0.000	-0.450	0.002	0.005

AS94_08335		heme ABC transporter ATP-binding protein		-0.953	0.000	0.000	-0.778	0.000	0.000
AS94_08340	*recQ*	ATP-dependent DNA helicase RecQ		-1.423	0.000	0.000	-1.288	0.000	0.000
AS94_08370		lipid kinase		-0.310	0.006	0.015	-0.758	0.000	0.000
AS94_08390		ribonucleotide reductase stimulatory protein		-1.282	0.000	0.000	-1.517	0.000	0.000
AS94_08395		ribonucleotide-diphosphate reductase subunit alpha		-0.892	0.000	0.000	-1.064	0.000	0.000
AS94_08405		iron ABC transporter permease		-1.854	0.000	0.000	-1.355	0.000	0.000
AS94_08410		iron ABC transporter permease		-1.730	0.000	0.000	-1.251	0.001	0.003
AS94_08485		hypothetical protein		-1.288	0.000	0.000	-1.285	0.000	0.000
AS94_08530	*uvrB*	excinuclease ABC subunit B		-0.825	0.000	0.000	-0.839	0.000	0.000
AS94_08565		hypothetical protein		-0.438	0.019	0.040	-0.585	0.002	0.005
AS94_08620	*gap*	glyceraldehyde-3-phosphate dehydrogenase		-0.363	0.002	0.004	-0.583	0.000	0.000
AS94_08635	*gpmI*	phosphoglyceromutase		-0.365	0.004	0.009	-0.415	0.001	0.003
AS94_08640	*eno*	enolase		-0.535	0.000	0.000	-0.637	0.000	0.000
AS94_08670		hypothetical protein		-0.495	0.000	0.000	-0.374	0.003	0.007
AS94_08715		virulence-associated protein E		-0.734	0.002	0.005	-0.790	0.001	0.003
AS94_08755		pathogenicity island protein		-0.649	0.000	0.000	-0.539	0.000	0.000
AS94_08870	*alr*	alanine racemase		-0.386	0.002	0.004	-0.404	0.001	0.003
AS94_08895	*kdpC*	potassium-transporting ATPase subunit C		-1.023	0.000	0.000	-1.320	0.000	0.000
AS94_08900	*kdpB*	potassium-transporting ATPase subunit B		-0.653	0.003	0.007	-0.834	0.000	0.000
AS94_08975	*thiE*	thiamine-phosphate pyrophosphorylase		-0.830	0.007	0.015	-0.807	0.008	0.019
AS94_08980	*thiM*	hydroxyethylthiazole kinase		-0.881	0.013	0.027	-0.824	0.020	0.043
AS94_09000	*ssb*	single-stranded DNA-binding protein		-1.486	0.000	0.000	-0.931	0.002	0.005
AS94_09140	*fba*	fructose-bisphosphate aldolase		-0.803	0.000	0.000	-1.039	0.000	0.000
AS94_09145		hypothetical protein		-0.534	0.004	0.009	-0.889	0.000	0.000
AS94_09170		hypothetical protein		-0.457	0.001	0.003	-0.341	0.015	0.034
AS94_09190		membrane protein		-1.187	0.000	0.000	-1.295	0.000	0.000
AS94_09195	*pdp*	pyrimidine-nucleoside phosphorylase	Y	-1.176	0.000	0.000	-1.117	0.000	0.000

AS94_09200	*deoC*	deoxyribose-phosphate aldolase	-0.624	0.000	0.000	-0.527	0.000	0.000
AS94_09210		general stress protein	-1.882	0.000	0.000	-1.297	0.000	0.000
AS94_09295		FmtB protein	-1.108	0.000	0.000	-1.238	0.000	0.000
AS94_09375		MarR family transcriptional regulator	-0.887	0.000	0.000	-0.683	0.000	0.000
AS94_09390		iron ABC transporter substrate-binding protein	-1.246	0.000	0.000	-1.066	0.001	0.002
AS94_09415		siderophore biosynthesis protein SbnE	-0.589	0.020	0.041	-0.589	0.020	0.044
AS94_09455		acetoin reductase	-1.696	0.000	0.000	-1.887	0.000	0.000
AS94_09485		superoxide dismutase	-2.825	0.000	0.000	-2.792	0.000	0.000
AS94_09515	*deoB*	phosphopentomutase	-0.774	0.000	0.000	-0.973	0.000	0.000
AS94_09545		2'_ 3'-cyclic nucleotide 2'-phosphodiesterase	-1.076	0.000	0.000	-1.224	0.000	0.000
AS94_09640	*isdI*	monooxygenase IsdI	-0.956	0.016	0.034	-1.073	0.007	0.016
AS94_09645		hypothetical protein	-1.375	0.000	0.000	-0.822	0.016	0.035
AS94_09700		4'-phosphopantetheinyl transferase	-0.897	0.000	0.000	-0.669	0.000	0.000
AS94_09705		hypothetical protein	-1.027	0.000	0.000	-0.977	0.000	0.000
AS94_09735		isochorismatase	-0.795	0.000	0.000	-0.661	0.000	0.000
AS94_09740		pyruvate decarboxylase	-0.706	0.000	0.000	-0.590	0.000	0.000
AS94_09750	*ptsG*	PTS glucose transporter subunit IIABC	-1.301	0.000	0.000	-1.286	0.000	0.000
AS94_09785	*nikE*	ABC transporter ATP-binding protein	-1.287	0.000	0.000	-1.172	0.000	0.000
AS94_09815		azoreductase	-1.128	0.000	0.000	-0.923	0.000	0.000
AS94_09825		sugar ABC transporter ATP-binding protein	-1.195	0.000	0.000	-0.998	0.000	0.001
AS94_09830		ABC transporter substrate-binding protein	-1.413	0.000	0.000	-1.004	0.000	0.000
AS94_09835		arabinogalactan ABC transporter permease	-1.200	0.000	0.000	-0.991	0.000	0.000
AS94_09840		arabinogalactan ABC transporter permease	-1.501	0.000	0.000	-1.504	0.000	0.000
AS94_09845		oxidoreductase	-2.229	0.000	0.000	-1.963	0.000	0.000
AS94_09850		NADH-dependent dehydrogenase	-2.559	0.000	0.000	-2.303	0.000	0.000
AS94_09855		xylose isomerase	-2.694	0.000	0.000	-2.370	0.000	0.000
AS94_09870		AraC family transcriptional regulator	-1.041	0.000	0.000	-1.307	0.000	0.000

AS94_09885	*pflB*	formate acetyltransferase		-2.478	0.000	0.000	-2.568	0.000	0.000
AS94_09890		pyruvate formate lyase-activating protein		-2.527	0.000	0.000	-2.617	0.000	0.000
AS94_09895		hypothetical protein		-1.630	0.000	0.000	-1.059	0.004	0.010
AS94_09900		glycerophosphoryl diester phosphodiesterase		-0.558	0.000	0.001	-0.478	0.003	0.006
AS94_09980		nitric oxide dioxygenase		-0.698	0.000	0.000	-0.419	0.004	0.011
AS94_09990		PTS glucose transporter subunit IIB		-1.095	0.000	0.000	-1.257	0.000	0.000
AS94_10030		iditol 2-dehydrogenase		-1.095	0.000	0.000	-0.994	0.000	0.000
AS94_10075		cell wall biosynthesis protein ScdA		-0.712	0.000	0.000	-0.491	0.000	0.000
AS94_10080		sensor histidine kinase		-0.941	0.000	0.000	-0.717	0.000	0.000
AS94_10085		LytR family transcriptional regulator		-0.828	0.000	0.001	-0.829	0.000	0.001
AS94_10100		GntR family transcriptional regulator		-0.750	0.000	0.000	-0.977	0.000	0.000
AS94_10105		phosphoenolpyruvate-dependent sugar PTS family porter_ EIIA 1		-2.185	0.000	0.000	-2.224	0.000	0.000
AS94_10110	*bglA*	aryl-phospho-beta-D-glucosidase		-1.718	0.000	0.000	-2.039	0.000	0.000
AS94_10115		hypothetical protein		-1.156	0.000	0.000	-1.335	0.000	0.000
AS94_10140		LacI family transcriptional regulator		-1.287	0.000	0.000	-1.483	0.000	0.000
AS94_10155		penicillin V acylase		-0.996	0.000	0.000	-0.848	0.000	0.000
AS94_10255		hypothetical protein		-0.598	0.000	0.001	-0.400	0.019	0.040
AS94_10320		formate/nitrite transporter		-0.783	0.000	0.000	-0.602	0.000	0.000
AS94_10355		pseudouridine-5'-phosphate glycosidase		-0.803	0.000	0.000	-0.616	0.002	0.005
AS94_10365		sialic acid transporter		-1.527	0.000	0.000	-0.743	0.000	0.000
AS94_10370	*nanA*	N-acetylneuraminate lyase	Y	-1.747	0.000	0.000	-0.910	0.000	0.000
AS94_10375		N-acetylmannosamine kinase		-0.868	0.000	0.000	-0.500	0.000	0.000
AS94_10380		RpiR family transcriptional regulator		-0.609	0.000	0.000	-0.540	0.000	0.000
AS94_10385		N-acetylmannosamine-6-phosphate 2-epimerase		-0.821	0.000	0.000	-0.813	0.000	0.000
AS94_10400		alpha/beta hydrolase		-0.831	0.000	0.000	-0.946	0.000	0.000
AS94_10475		glyoxalase		-0.559	0.009	0.020	-0.553	0.010	0.022
AS94_10480		luciferase		-1.058	0.000	0.000	-1.150	0.000	0.000
AS94_10485		FMN reductase		-0.591	0.015	0.032	-0.957	0.000	0.000

AS94_10490		membrane protein	-0.960	0.000	0.000	-1.031	0.000	0.000
AS94_10665		hypothetical protein	-1.408	0.000	0.000	-1.321	0.000	0.000
AS94_10675		phosphoglycerate mutase	-0.449	0.012	0.026	-0.416	0.020	0.044
AS94_10700		alkyl hydroperoxide reductase subunit C	-0.477	0.000	0.000	-0.366	0.001	0.002
AS94_10705	*nfrA*	NADPH-dependent oxidoreductase	-0.566	0.000	0.001	-0.712	0.000	0.000
AS94_10730	*xpt*	xanthine phosphoribosyltransferase	-1.458	0.000	0.000	-1.523	0.000	0.000
AS94_10735		xanthine permease	-1.145	0.000	0.000	-1.247	0.000	0.000
AS94_10740	*guaB*	inosine 5'-monophosphate dehydrogenase	-1.063	0.000	0.000	-1.206	0.000	0.000
AS94_10745	*guaA*	GMP synthase	-0.806	0.000	0.000	-1.191	0.000	0.000
AS94_10815		membrane protein	-1.762	0.000	0.000	-1.961	0.000	0.000
AS94_10820		3-beta hydroxysteroid dehydrogenase	-1.256	0.000	0.000	-1.140	0.000	0.000
AS94_10890		hypothetical protein	-0.978	0.000	0.000	-1.002	0.000	0.000
AS94_10895		hypothetical protein	-1.471	0.003	0.006	-1.503	0.002	0.005
AS94_10940		NADH dehydrogenase subunit 5	-0.803	0.000	0.000	-0.926	0.000	0.000
AS94_10945		hypothetical protein	-0.713	0.000	0.000	-0.629	0.000	0.000
AS94_10965		carboxylesterase	-0.830	0.000	0.000	-0.800	0.000	0.000
AS94_10970		sodium-dependent transporter	-0.471	0.008	0.017	-0.903	0.000	0.000
AS94_11000		N-acetylmuramoyl-L-alanine amidase	-0.384	0.001	0.002	-0.457	0.000	0.000
AS94_11020		hypothetical protein	-0.830	0.000	0.000	-1.007	0.000	0.000
AS94_11025		hypothetical protein	-0.701	0.000	0.000	-0.535	0.002	0.004
AS94_11075	*dnaX*	DNA polymerase III subunit gamma/tau	-0.323	0.006	0.014	-0.370	0.002	0.004
AS94_11080		hypothetical protein	-0.618	0.002	0.005	-0.499	0.013	0.029
AS94_11090		adhesin	-1.569	0.000	0.000	-1.330	0.000	0.000
AS94_11095		2-oxoglutarate translocator	-0.887	0.000	0.000	-0.947	0.000	0.000
AS94_11100		chloramphenicol-sensitive protein RarD	-0.445	0.009	0.020	-0.508	0.003	0.007
AS94_11110		nickel transporter NixA	-0.701	0.000	0.000	-0.893	0.000	0.000
AS94_11115		N-acetyltransferase	-0.617	0.000	0.000	-0.821	0.000	0.000
AS94_11145		cold-shock protein	-0.635	0.000	0.000	-0.592	0.000	0.000
AS94_11150		Cro/Cl family transcriptional regulator	-1.114	0.000	0.000	-1.032	0.000	0.000

AS94_11155		hypothetical protein	-1.053	0.000	0.000	-0.629	0.002	0.006
AS94_11160		hypothetical protein	-1.163	0.000	0.000	-0.695	0.001	0.003
AS94_11225		carbohydrate kinase	-1.327	0.000	0.000	-1.238	0.000	0.000
AS94_11265	*rplI*	50S ribosomal protein L9	-0.585	0.000	0.000	-0.666	0.000	0.000
AS94_11270	*dnaB*	replicative DNA helicase	-0.915	0.000	0.000	-1.089	0.000	0.000
AS94_11400		recombinase RecA	-0.609	0.002	0.005	-0.828	0.000	0.000
AS94_11465	*ilvE*	branched-chain amino acid aminotransferase	-0.377	0.000	0.001	-0.363	0.001	0.002
AS94_11480		molecular chaperone	-0.896	0.000	0.000	-1.156	0.000	0.000
AS94_11495		elongation factor Tu	-0.311	0.006	0.015	-0.374	0.001	0.003
AS94_11520	*rpoC*	DNA-directed RNA polymerase subunit beta'	-0.414	0.000	0.000	-0.472	0.000	0.000
AS94_11525	*rpoB*	DNA-directed RNA polymerase subunit beta	-0.292	0.009	0.019	-0.271	0.015	0.033
AS94_11600	*gltX*	glutamyl-tRNA synthase	-0.584	0.000	0.000	-0.585	0.000	0.000
AS94_11660		adhesin	-2.593	0.000	0.000	-2.529	0.000	0.000
AS94_11670		pyrrolidone-carboxylate peptidase	-1.024	0.000	0.000	-1.052	0.000	0.000
AS94_11690		polysaccharide deacetylase	-1.341	0.000	0.000	-1.007	0.002	0.006
AS94_11695		ATP phosphoribosyltransferase	-2.684	0.000	0.000	-2.797	0.000	0.000
AS94_11700	*hisG*	ATP phosphoribosyltransferase	-2.917	0.000	0.000	-2.726	0.000	0.000
AS94_11705	*hisD*	histidinol dehydrogenase	-2.248	0.000	0.000	-2.226	0.000	0.000
AS94_11710		histidinol-phosphate aminotransferase	-2.002	0.000	0.000	-1.979	0.000	0.000
AS94_11715		imidazoleglycerol-phosphate dehydratase	-1.603	0.009	0.020	-1.428	0.020	0.044
AS94_11720	*hisH*	imidazole glycerol phosphate synthase	-1.820	0.001	0.002	-1.895	0.001	0.002
AS94_11725	*hisA*	1-(5-phosphoribosyl)-5-[(5-phosphoribosylamino)methylideneamino] imidazole-4-carboxamide isomerase	-1.342	0.000	0.001	-1.252	0.001	0.002
AS94_11730	*hisF*	imidazole glycerol phosphate synthase	-0.959	0.004	0.010	-0.932	0.005	0.013
AS94_11735	*hisE*	phosphoribosyl-AMP cyclohydrolase	-0.876	0.000	0.000	-0.752	0.001	0.003
AS94_11760		N-glycosyltransferase	-1.459	0.000	0.000	-1.757	0.000	0.000
AS94_11830		adhesin	-0.661	0.000	0.000	-0.674	0.000	0.000
AS94_11835	*secY2*	preprotein translocase subunit SecY	-1.029	0.000	0.001	-1.093	0.000	0.000

AS94_11840		Accessory Sec system protein Asp1			-1.428	0.000	0.000	-1.249	0.000	0.000
AS94_11845		accessory secretory protein Asp2			-1.126	0.000	0.000	-1.085	0.000	0.000
AS94_11850		Accessory Sec system protein Asp3			-0.998	0.001	0.002	-0.775	0.009	0.022
AS94_11890	*manA*	mannose-6-phosphate isomerase			-2.854	0.000	0.000	-3.259	0.000	0.000
AS94_11895	*manP*	PTS mannose transporter subunit IIABC		Y	-2.655	0.000	0.000	-2.737	0.000	0.000
AS94_11905		hypothetical protein			-0.654	0.001	0.004	-0.564	0.006	0.015
AS94_11925	*arcA*	arginine deiminase	Y		-1.650	0.000	0.000	-1.290	0.000	0.000
AS94_11930	*argF*	ornithine carbamoyltransferase	Y	Y	-0.787	0.000	0.000	-0.449	0.001	0.004
AS94_11960		Fur family transcriptional regulator			-0.743	0.000	0.000	-0.644	0.000	0.000
AS94_11980		membrane protein			-0.302	0.021	0.042	-0.296	0.023	0.049
AS94_12005		membrane protein			-0.503	0.002	0.004	-0.661	0.000	0.000
AS94_12470		ferritin			-0.759	0.000	0.000	-0.897	0.000	0.000
AS94_12565		membrane protein			-0.577	0.000	0.001	-0.648	0.000	0.000
AS94_12570	*nadE*	NAD synthetase			-0.437	0.002	0.006	-0.443	0.002	0.005
AS94_12585		prephenate dehydratase			-0.689	0.000	0.000	-0.617	0.000	0.000
AS94_12605		pyrophosphatase			-0.684	0.000	0.000	-0.864	0.000	0.000
AS94_12610		aldehyde dehydrogenase			-0.768	0.000	0.000	-0.845	0.000	0.000
AS94_12785		hypothetical protein			-1.400	0.000	0.000	-1.746	0.000	0.000
AS94_12815		peptidase M23			-0.821	0.000	0.001	-0.945	0.000	0.000
AS94_12865		nitroreductase			-0.506	0.000	0.000	-0.400	0.002	0.006
AS94_12875	*hld*	delta-hemolysin	Y		-0.725	0.000	0.000	-0.296	0.004	0.010
AS94_12880	*agrB*	accessory gene regulator B	Y		-0.893	0.000	0.000	-0.784	0.000	0.000
AS94_12890	*agrC*	histidine kinase	Y		-0.607	0.000	0.000	-0.536	0.000	0.000
AS94_12895	*agrA*	histidine kinase	Y		-0.952	0.000	0.000	-0.901	0.000	0.000
AS94_12900	*pfkB*	fructokinase			-0.915	0.000	0.000	-1.091	0.000	0.000
AS94_12905		sucrose-6-phosphate hydrolase			-0.487	0.000	0.000	-0.423	0.000	0.000
AS94_12945		O-sialoglycoprotein endopeptidase			-0.317	0.011	0.024	-0.444	0.000	0.001
AS94_13015		hypothetical protein			-3.718	0.007	0.017	-3.340	0.017	0.036
AS94_13445	*hemG*	protoporphyrinogen oxidase			-0.334	0.021	0.043	-0.459	0.001	0.004

AS94_13475			cell-cycle regulation protein HIT	-1.061	0.000	0.000	-1.246	0.000	0.000
AS94_12245		ϕSA169	hypothetical protein	-2.279	0.000	0.001	-1.619	0.014	0.030
AS94_12265			hypothetical protein	-0.776	0.016	0.033	-1.225	0.000	0.000
AS94_12285			DNA replication protein DnaC	-0.520	0.008	0.018	-0.648	0.001	0.003
AS94_13030		mutual prophage	hypothetical protein	-1.362	0.000	0.000	-1.393	0.000	0.000
AS94_13035	*entG*		enterotoxin	-0.920	0.000	0.000	-0.712	0.001	0.003
AS94_13070			autolysin	-2.341	0.000	0.000	-0.873	0.000	0.000
AS94_13075			holin	-3.249	0.000	0.000	-1.556	0.000	0.000
AS94_13095			hypothetical protein	-2.348	0.000	0.000	-0.651	0.002	0.006
AS94_13100			minor structural protein	-2.067	0.000	0.000	-0.542	0.001	0.003
AS94_13110			peptidase	-2.176	0.000	0.000	-0.459	0.021	0.044
AS94_13120			tail protein	-2.112	0.000	0.000	-0.489	0.000	0.000
AS94_13135			tail protein	-2.071	0.000	0.000	-0.647	0.000	0.001
AS94_13195			transcriptional regulator	-1.929	0.000	0.000	-1.194	0.000	0.000
AS94_13200			helicase	-1.557	0.000	0.000	-0.662	0.002	0.006
AS94_13205			hypothetical protein	-1.435	0.000	0.000	-0.686	0.001	0.002
AS94_13220			hypothetical protein	-1.711	0.000	0.000	-1.089	0.000	0.000
AS94_13350			hypothetical protein	-1.164	0.007	0.015	-1.035	0.016	0.036
AS94_13360			hypothetical protein	-1.727	0.000	0.000	-1.755	0.000	0.000
AS94_13370			hypothetical protein	-0.962	0.000	0.000	-0.611	0.000	0.000
AS94_13375			XRE family transcriptional regulator	-1.445	0.000	0.000	-0.882	0.000	0.001
AS94_13390			hypothetical protein	-0.899	0.000	0.000	-0.551	0.000	0.000
