# Supplementary material for: Transcriptome Analyses of Prophage in Mediating Persistent Methicillin-Resistant Staphylococcus aureus Endovascular Infection
Source: Genes (Basel). 2022 Aug 25;13(9):1527. doi: 10.3390/genes13091527 (PMC9498598; doi:10.3390/genes13091527)
Supplement: Supplementary file 1 [file genes-13-01527-s001.zip › Table S9.pdf]

Table S9. DEGs up-regulated by both ϕSA169 and MRSA genetic backgrounds

locus	gene	group	product	301-188::ϕSA169 vs. 301-188			300-169 vs. 301-188			300-169 vs. 301-188::ϕSA169		
				log_2(fold change)	*p* value	*p* adj	log_2(fold change)	*p* value	*p* adj	log_2(fold change)	*p* value	*p* adj
AS94_04115	*fabF*	host genes	3-oxoacyl-ACP synthase	0.423	0.000	0.008	0.719	0.000	0.000	0.295	0.012	0.027
AS94_04120	*fabH*		3-oxoacyl-ACP synthase	0.577	0.000	0.005	1.095	0.000	0.000	0.517	0.001	0.002
AS94_04780			amino acid permease	0.507	0.000	0.001	1.289	0.000	0.000	0.782	0.000	0.000
AS94_05540			glycine/betaine ABC transporter permease	0.439	0.001	0.020	1.635	0.000	0.000	1.196	0.000	0.000
AS94_06080			hypothetical protein	0.661	0.000	0.000	1.519	0.000	0.000	0.859	0.000	0.000
AS94_06310			sodium:glutamate symporter	0.479	0.002	0.039	1.092	0.000	0.000	0.613	0.000	0.000
AS94_11275	*purA*		adenylosuccinate synthetase	0.471	0.003	0.047	2.591	0.000	0.000	2.120	0.000	0.000
AS94_11985			multidrug ABC transporter ATP-binding protein	0.417	0.001	0.011	0.925	0.000	0.000	0.508	0.000	0.000
AS94_12030			general stress protein	0.375	0.001	0.012	0.703	0.000	0.000	0.329	0.002	0.006
AS94_12410			ribonuclease BN	0.420	0.003	0.044	1.228	0.000	0.000	0.808	0.000	0.000
AS94_12040		ϕSA169	hypothetical protein	7.107	0.000	0.000	8.459	0.000	0.000	1.352	0.014	0.031
AS94_12055			autolysin	9.937	0.000	0.000	10.844	0.000	0.000	0.907	0.000	0.000
AS94_12070			tail protein	10.966	0.000	0.000	11.976	0.000	0.000	1.010	0.000	0.000
AS94_12075			cell wall hydrolase	11.731	0.000	0.000	12.788	0.000	0.000	1.057	0.000	0.000
AS94_12090			hypothetical protein	7.301	0.000	0.000	8.652	0.000	0.000	1.351	0.009	0.020
AS94_12095			hypothetical protein	11.292	0.000	0.000	12.193	0.000	0.000	0.901	0.000	0.000
AS94_12100			minor structural protein	11.761	0.000	0.000	12.567	0.000	0.000	0.806	0.000	0.000
AS94_12105			peptidase	11.625	0.000	0.000	12.720	0.000	0.000	1.095	0.000	0.000
AS94_12110			phage tail protein	10.251	0.000	0.000	11.456	0.000	0.000	1.205	0.000	0.000
AS94_12115			membrane protein	13.073	0.000	0.000	13.916	0.000	0.000	0.843	0.000	0.000
AS94_12120			phi 11	8.667	0.000	0.000	9.902	0.000	0.000	1.235	0.001	0.002
AS94_12125			hypothetical protein	9.203	0.000	0.000	10.462	0.000	0.000	1.259	0.000	0.000
AS94_12130			tail protein	11.175	0.000	0.000	12.239	0.000	0.000	1.063	0.000	0.000
AS94_12135			phi 11	8.782	0.000	0.000	9.806	0.000	0.000	1.024	0.003	0.006

AS94_12140	hypothetical protein	7.889	0.000	0.000	9.238	0.000	0.000	1.349	0.002	0.004
AS94_12145	hypothetical protein	7.152	0.000	0.000	8.869	0.000	0.000	1.717	0.001	0.002
AS94_12150	phage head-tail adapter protein	8.690	0.000	0.000	10.259	0.000	0.000	1.569	0.000	0.000
AS94_12155	phi 11	6.853	0.000	0.000	8.348	0.000	0.000	1.495	0.010	0.022
AS94_12160	hypothetical protein	11.809	0.000	0.000	12.909	0.000	0.000	1.100	0.000	0.000
AS94_12165	phage capsid protein	11.968	0.000	0.000	13.409	0.000	0.000	1.440	0.000	0.000
AS94_12175	phage head morphogenesis protein	11.205	0.000	0.000	12.222	0.000	0.000	1.017	0.000	0.000
AS94_12180	phage portal protein	11.607	0.000	0.000	12.555	0.000	0.000	0.948	0.000	0.000
AS94_12185	hypothetical protein	10.893	0.000	0.000	12.014	0.000	0.000	1.121	0.000	0.000
AS94_12190	terminase	10.141	0.000	0.000	11.070	0.000	0.000	0.929	0.000	0.000
AS94_12345	BRO-like protein	11.879	0.000	0.000	12.337	0.000	0.000	0.458	0.006	0.015
AS94_12375	integrase	10.382	0.000	0.000	10.916	0.000	0.000	0.534	0.021	0.045
