# Supplementary material for: Transcriptome Analyses of Prophage in Mediating Persistent Methicillin-Resistant Staphylococcus aureus Endovascular Infection
Source: Genes (Basel). 2022 Aug 25;13(9):1527. doi: 10.3390/genes13091527 (PMC9498598; doi:10.3390/genes13091527)
Supplement: Supplementary file 1 [file Figure S1.pdf › Figure S1.pdf]

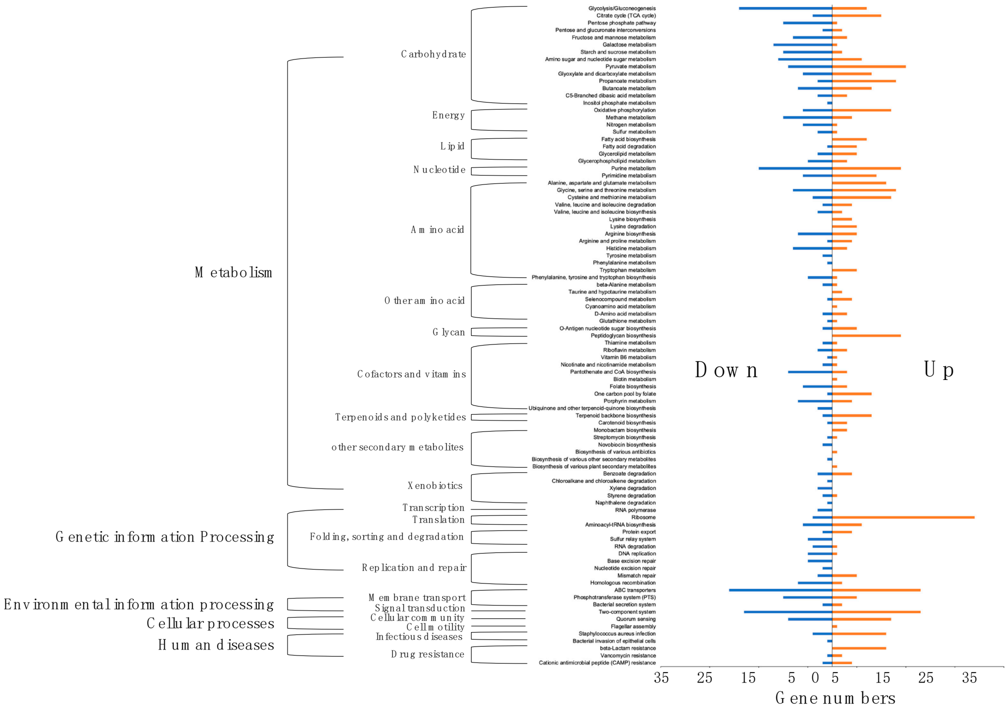

Figure S1. KEGG analysis of the DEGs impacted by the distinct genetic backgrounds of 300-169 and 301-188. Most of the DEGs were related to different types of metabolisms.
